# Multi-omics analysis identifies repurposing bortezomib in the treatment of kidney-, nervous system-, and hematological cancers

**DOI:** 10.1038/s41598-024-62339-x

**Published:** 2024-08-10

**Authors:** Peter Larsson, Maxim Olsson, Sithumini Sarathchandra, Anna Fäldt Beding, Eva Forssell-Aronsson, Anikó Kovács, Per Karlsson, Khalil Helou, Toshima Z. Parris

**Affiliations:** 1 Department of Oncology, Institute of Clinical Sciences, Sahlgrenska Academy, University of Gothenburg, Gothenburg, Sweden; 2 Sahlgrenska Center for Cancer Research, Sahlgrenska Academy, University of Gothenburg, Gothenburg, Sweden; 3 Department of Chemistry and Molecular Biology, University of Gothenburg, Gothenburg, Sweden; 4 Department of Oncology, Southern Älvsborg Hospital, Borås, Sweden; 5 Department of Medical Radiation Sciences, Institute of Clinical Sciences, Sahlgrenska Academy, University of Gothenburg, Gothenburg, Sweden; 6 Department of Medical Physics and Biomedical Engineering, Sahlgrenska University Hospital, Gothenburg, Sweden; 7 Department of Clinical Pathology, Sahlgrenska University Hospital, Gothenburg, Sweden; 8 Department of Oncology, Sahlgrenska University Hospital, Gothenburg, Sweden

**Keywords:** Drug repurposing, Proteasome inhibitor, Bortezomib, Gene expression profiling, Mutation profiling, Cancer, Pharmacology, Chemotherapy, Chemotherapy

## Abstract

Repurposing of FDA-approved drugs is a quick and cost-effective alternative to de novo drug development. Here, we identify genes involved in bortezomib sensitivity, predict cancer types that may benefit from treatment with bortezomib, and evaluate the mechanism-of-action of bortezomib in breast cancer (BT-474 and ZR-75–30), melanoma (A-375), and glioblastoma (A-172) cells in vitro. Cancer cell lines derived from cancers of the blood, kidney, nervous system, and skin were found to be significantly more sensitive to bortezomib than other organ systems. The in vitro studies confirmed that although bortezomib effectively inhibited the β5 catalytic site in all four cell lines, cell cycle arrest was only induced in G2/M phase and apoptosis in A-375 and A-172 after 24h. The genomic and transcriptomic analyses identified 33 genes (e.g. *ALDH18A1*, *ATAD2*) associated with bortezomib resistance. Taken together, we identified biomarkers predictive of bortezomib sensitivity and cancer types that might benefit from treatment with bortezomib.

## Introduction

The 26S proteasome is a large barrel-shaped protein complex, consisting of up to three subunits (one 20S and one or two 19S subunits) and three catalytic sites (β1 [caspase-like], β2 [trypsin-like], and β5 [chymotrypsin-like]), that is tasked with degrading up to 90% of temporary and misfolded, ubiquitinated proteins in the cell^1–4^. Proteolysis is necessary to prevent protein accumulation, provide the cell with new building blocks (amino acids) for new proteins, regulate certain cellular processes (e.g. cell cycle, immune response and apoptosis), and control protein quality^5,6^. Although abnormally high proteasome activity is found in neoplastic tissue, cancer stem cells or cancer cells with stem cell characteristics have been associated with decreased proteasome activity^7,8^. Elevated proteasome activity is caused by several factors, including mutations resulting in an accumulation of misfolded proteins in the cell^9^. Inhibition of proteasome catalytic activity can in turn lead to accumulation of proteins, thereby resulting in the disruption of key cellular processes, a reduced amount of free amino acids available for protein synthesis, cell cycle arrest, and ultimately cell death^10,11^.

Bortezomib (VELCADE®, formerly PS-341) was the first proteasome inhibitor to pass clinical trials and be approved by US Food and Drug Administration (FDA) and the European Medicines Agency (EMA) as first-line treatment for patients diagnosed with multiple myeloma^12^. Bortezomib and proteasome inhibitors in general primarily bind to the β5 catalytic site and secondarily to the β1 and β2 sites at higher concentrations^9,13^. A number of proteasome inhibitors have been developed from various chemical classes (e.g. boronate, epoxyketone), binding kinetics (reversible or irreversible binding), and routes of administration (intravenous, subcutaneous, and oral)^14^. Bortezomib is a dipeptide boronic acid analog with a half-life of 100 min that reversibly inhibits the chymotrypsin-like activity of the β5 catalytic site^15^. As bortezomib-associated side effects and treatment resistance have become an issue, several second-generation proteasome inhibitors such as carfilzomib, delanzomib, and epoxomicin were developed^16,17^.

Drug development is a time consuming and costly process with high risk of failure and unknown drug side effects. In contrast, drug repurposing (also called drug repositioning) is a relatively quick, cost effective, and low-risk process that can potentially identify new indications for FDA/EMA-approved drugs with previously known side effects (patient safety)^18^. Compared to drug repurposing, the conventional drug development process takes about 5–7 years longer from target discovery to clinical implementation^18,19^. A very common way of drug repurposing is to identify “positive” side effects of drug treatment that emerge during or shortly after clinical trials that could be used in the treatment of other diseases. Tamoxifen and aspirin are noteworthy examples of drug repurposing. Tamoxifen was originally intended to be used as a contraceptive that failed in clinical trials. Later studies revealed Tamoxifen to have antitumor effects; the drug is currently first-line treatment for hormone-positive breast cancer^20,21^. Besides its use as a painkiller, long-term daily aspirin use has been linked to reduced risk of colorectal cancer^22^. Here, we identify bortezomib as a multifaceted drug that significantly decreases cell viability in cancer cell lines derived from hematological malignancies, kidney cancer, as well as cancers of the nervous system. We also pinpoint important genomic and transcriptomic profiles associated with bortezomib sensitivity. It is therefore important to predict specific cancer types where patients could benefit from treatment with bortezomib in order to obtain prolonged survival and lower risk of recurrence.

## Results

### Cancers of the blood, kidney, nervous system and skin were most sensitive to bortezomib

**Figure 1:**
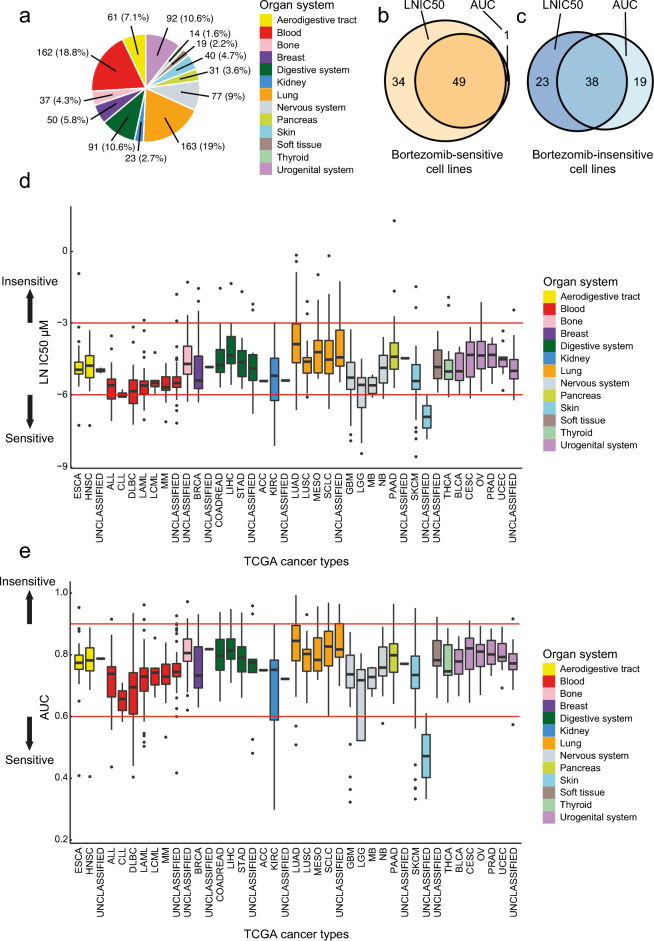
Drug response data from Genomics Drug Sensitivity in Cancer (GDSC) were compiled from the GDSC1 (n = 791) and GDSC2 (n = 756) datasets, and merged into a single dataset containing 860 cancer cell lines (duplicates found in both datasets were excluded). (**a**) Pie charts depicting the number (and percentage) of cell lines associated with each organ system. Venn diagram showing the number of cancer cell lines classified as (**b**) bortezomib-sensitive (n = 84) and (**c**) bortezomib-insensitive (n = 80) based on the logarithmic half-maximal inhibitory concentration (LNIC50: < -6 for sensitivity and > -3 for insensitivity) and/or area under the curve (AUC: < 0.6 for sensitivity and > 0.9 for insensitivity) values. Boxplots depicting sensitivity to bortezomib according to (d) LNIC50 and (e) AUC, with the red horizontal lines illustrating thresholds for bortezomib-sensitivity (LNIC50 < -6 and AUC < 0.6) and insensitivity (LNIC50 > -3 and AUC > 0.9). Using both LNIC50 and AUC values, 49 bortezomib-sensitive and 38 bortezomib-insensitive cell lines were identified for use in subsequent analyses.

To assess cancer cell line sensitivity to bortezomib (proteasome inhibitor), we used two datasets (Sanger and Massachusetts General Hospital) from the Genomics of Drug Sensitivity in Cancer (GDSC) database that together contained drug sensitivity data (IC50 and AUC) for 860 cancer cell lines originating from 13 organ systems (30 cancer types and 11 unclassified groups, Fig. 1a and Supplementary Table 1). In total, 49/860 (5.7%) cancer cell lines were classified as bortezomib-sensitive and 38 (4.4%) as bortezomib-insensitive (Fig. 1b,c). These data demonstrated that hematologic malignancies (ALL, CLL, DLBC, LAML, LCML, MM, and Unclassified) and cancers of the nervous system (GBM, LGG, MB, and NB), kidney (KIRC and Unclassified), and skin (SKCM and Unclassified) were shown to be sensitive to bortezomib, while cancer of the lung (LUAD, LUSC, MESO, SCLC, and Unclassified), urogenital (BLCA, CESC, OV, PRAD, UCEC, and Unclassified), and digestive organ systems (COADREAD, LIHC, STAD, and Unclassified) were bortezomib-insensitive (Fig. 1d,e). However, an analysis of the distribution of organ systems in the whole cohort versus the bortezomib-insensitive or bortezomib-sensitive groups identified lung cancer to be significantly (*P* = 4.6E-08) overrepresented in the bortezomib-insensitive group and blood (*P* = 0.0007), kidney (*P* = 0.0002), nervous system (*P* = 0.0080) and skin (*P* = 0.0004) to be significantly overrepresented in the bortezomib-sensitive group. Although bortezomib-sensitivity was heterogeneous within all organ systems, cell lines derived from e.g. lung cancer and hematologic malignancies were present in both bortezomib-sensitivity groups.

**Figure 2:**
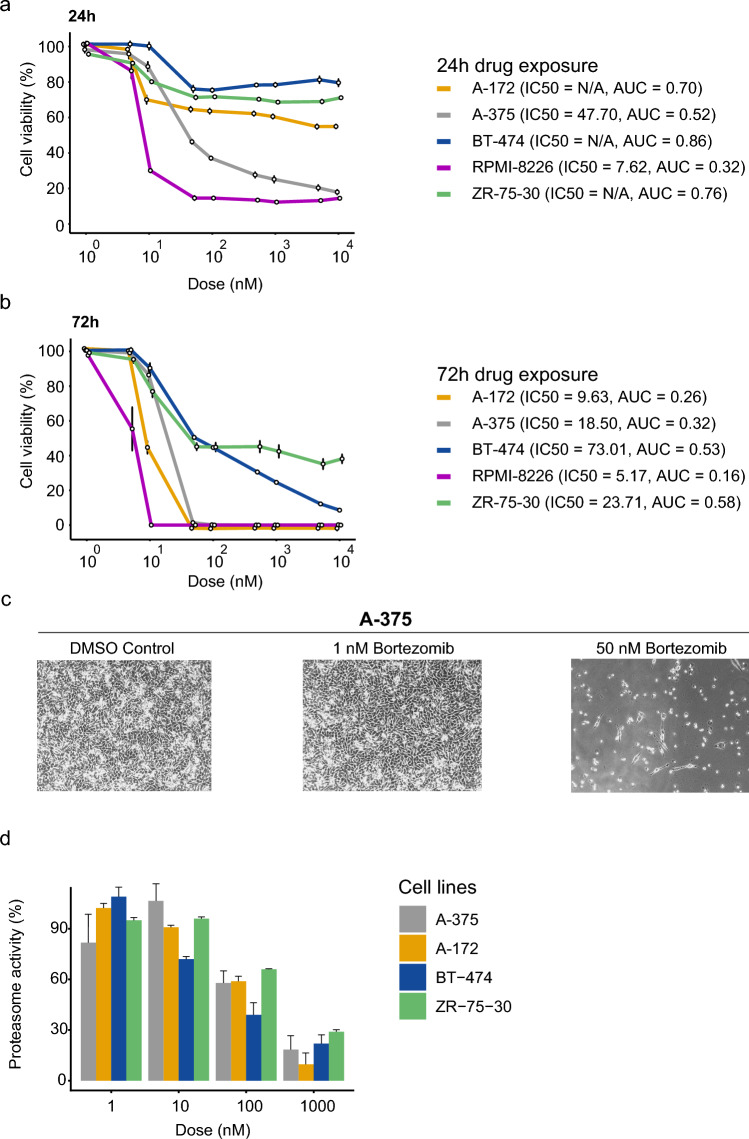
In vitro analysis confirms that A-375 melanoma, A-172 glioblastoma, and RPMI-8226 multiple myeloma cells are more sensitive to bortezomib than BT-474 and ZR-75–30 breast cancer cells, which is consistent with the GDSC1/GDSC2 dataset. Cell viability analysis depicting the effect of (**a**) 24h and (**b**) 72h bortezomib treatment on half-maximal inhibitory concentration (IC50) and area under the curve (AUC) metrics in five cancer cell lines (A-172, A-375, BT-474, RPMI-8226, and ZR-75–30). (**c**) Representative images showing the effect of different bortezomib concentrations on cell density for the A-375 cell line. (d) Bar chart showing inhibition of the β5 proteasome catalytic activity at different bortezomib concentrations (0–1000 nM) after 2h drug exposure.

**Figure 3:**
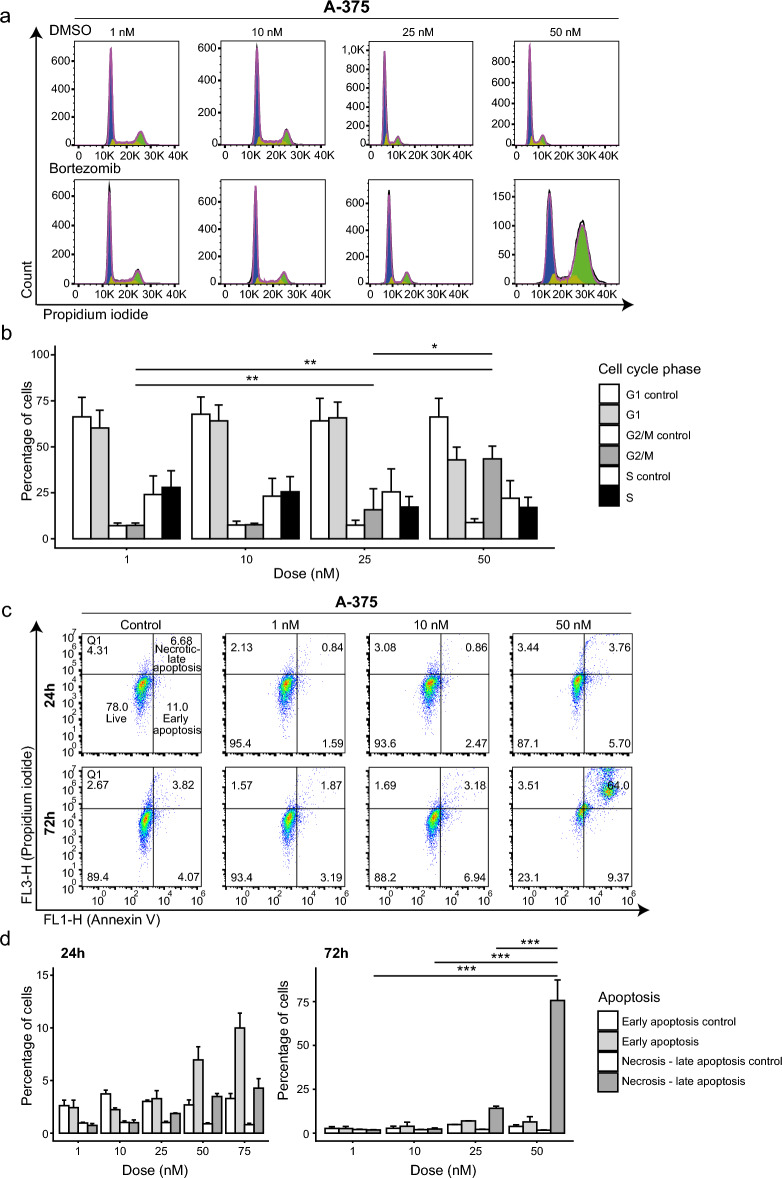
In vitro analysis of bortezomib-induced cell cycle arrest and apoptosis in A-375 melanoma cells. (**a**,**b**) Cell cycle distribution analysis showing G2/M cell cycle arrest in A-375 cells following 24h treatment with 50 nM bortezomib. The one-way ANOVA test was used to calculate statistically significant differences between the proportion of cells present in different cell cycle phases (G1, S, and G2/M phases). Not significant (*P* > 0.05); *P < 0.05; **P ≤ 0.01; ***P ≤ 0.001. Only statistically significant differences are shown in the figure. (**c**,**d**) Annexin V analysis showing a high proportion of early apoptotic A-375 melanoma cells after 24h bortezomib treatment, and necrotic – late apoptotic cells after 72h treatment with 50 nM bortezomib. The one-way ANOVA test was used to determine statistically significant differences between the proportion of necrotic-late apoptotic or early apoptotic cells at different bortezomib concentrations. Not significant (*P* > 0.05); *P < 0.05; **P ≤ 0.01; ***P ≤ 0.001. Only bars with significant differences between the same state and at different bortezomib concentrations were shown in the figure.

To examine the mechanism-of-action for bortezomib (induction of cell cycle arrest, apoptosis, proteasome inhibition) and validate bortezomib sensitivity in human cancer cells, three cell lines from the bortezomib-sensitive group (A-172, A-375, and RPMI-8226) and two from the bortezomib-insensitive group (BT-474 and ZR-75–30) were treated with bortezomib for 24h and 72h. As expected, A-172, A-375, and RPMI-8226 cells had lower cell viability (according to IC50 and AUC values) after treatment than BT-474 and ZR-75–30 cells (Fig. 2a,b). Massive cell death for the A-172 and A-375 cell lines was also observed at 50 nM (Fig. 2c). At 1000 nM bortezomib, about 80% inhibition of the proteasome β5 catalytic site was shown in all cell lines (Fig. 2d). At a concentration of 50 nM, cell cycle arrest in G2/M phase was observed in A-172 and A-375 cells but could not be determined in the other cell lines (Fig. 3a,b). Evaluation of the induction of early and late apoptosis after 24h or 72h treatment time revealed a significant increase in necrotic-late apoptotic and early apoptotic cells at 50 nM after 72h treatment in A-172 and A-375 cells (Fig. 3c,d). Notably, A-172 and A-375 cells did not survive the 72h treatment period with 100 nM bortezomib. Furthermore, no significant increase in necrotic-late apoptotic and early apoptotic cells could not be detected in BT-474 or ZR-75–30 cell lines. As expected, A-172 (24h treatment [IC50 = N/A; AUC = 0.74]; 72h treatment [IC50 = 10.3 nM; AUC = 0.28], A-375 (24h treatment [IC50 = 42 nM; AUC = 0.76]; 72h treatment [IC50 = 11.2 nM; AUC = 0.29], and RPMI-8226 (24h treatment [IC50 = 7.6 nM; AUC = 0.32]; 72h treatment [IC50 = 5.2 nM; AUC = 0.16]) were most sensitive to bortezomib. Although the β5 catalytic site was not fully inhibited, A-172 and A-375 cells still underwent apoptosis.

### Mutations in the *MUC12*, *PCDH15*, *RYR1* and *SPTA1* genes were most prevalent in bortezomib-insensitive cell lines

**Figure 4:**
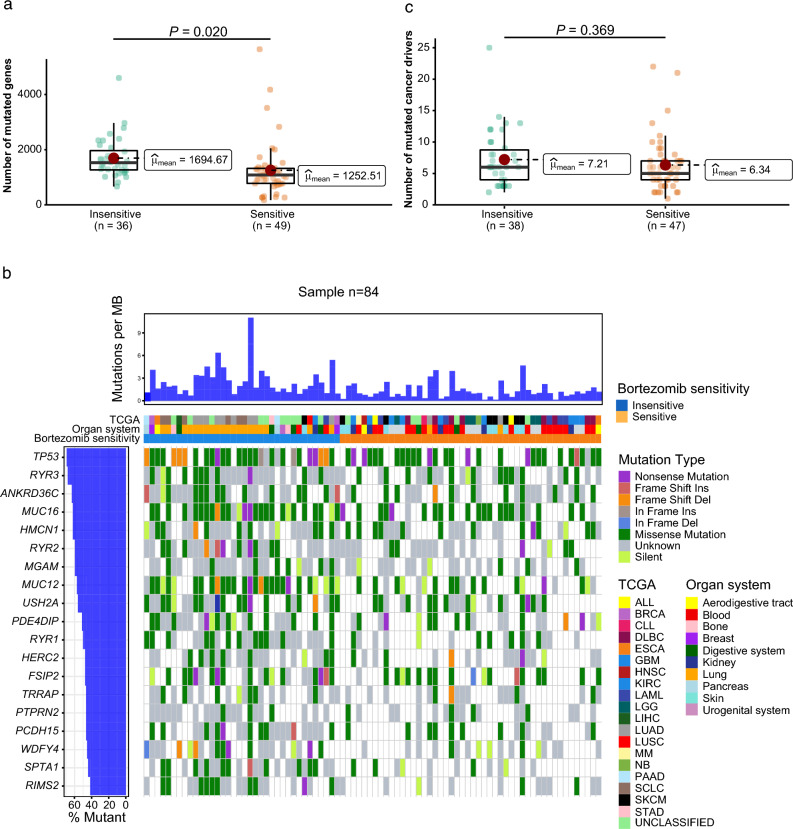
Mutation frequency and mutation type in the bortezomib-insensitive and sensitive groups. (a) Box plots depicting differences in mutation frequency between bortezomib-sensitive and bortezomib-insensitive cancer cell lines using the ggstatsplot R package with ggbetweenstats and Welch’s t-test. (**b**) Waterfall plot depicting the mutation frequency of genes in both sensitivity groups. T-test (*P* < 0.05) was used to stratify genes with significantly elevated mutation frequencies between the sensitivity groups. The most deleterious mutations are shown if several mutations affected the same gene. (**c**) Box plots depicting differences in mutation frequency for cancer drivers between bortezomib-sensitive and bortezomib-insensitive cancer cell lines using Welch’s t-test.

To determine an association between amino acid mutation frequency and bortezomib sensitivity, we then retrieved mutation data for the bortezomib-insensitive and bortezomib-sensitive cell lines from the Sanger COSMIC Cell Lines Project and Sanger Institute Cell Model Passports. Analysis of mutation frequency in both bortezomib-sensitivity groups (bortezomib-sensitive and bortezomib-insensitive) revealed a significantly higher mutation burden in bortezomib-insensitive cell lines (*P* = 0.02; Fig. 4a). We then determined the 50 most frequently mutated genes in each sensitivity group (Supplementary Fig. 1a-b). By comparing the mutation rate of these genes in the different sensitivity groups, we were able to identify 19 genes (*ANKRD36C*, *FSIP2*, *HERC2*, *HMCN1*, *MGAM*, *MUC12*, *MUC16*, *PCDH15*, *PDE4DIP*, *PTPRN2*, *RIMS2*, *RYR1-3*, *SPTA1*, *TP53*, *TRRAP*, *USH2A*, and *WDFY4*) that had significantly elevated mutation rates in the bortezomib-insensitive group (Fig. 4b and Supplementary Tables 2–3; *P* < 0.05). Gene Ontology analysis showed that these 19 genes play a vital role in DNA repair, gene transcription, and cell cycle. The mutation frequency of the 19 genes was then validated using the Cancer Cell Line Encyclopedia (CCLE) dataset, thereby showing that five of the 19 genes (*MUC12*, *MUC16*, *RIMS2*, *RYR2*, and *SPTA1*) were more frequently mutated in the bortezomib-insensitive cell lines (Supplementary Table 4). Nevertheless, there was no significant difference in the frequency of mutated cancer drivers between the two sensitivity groups (*P* = 0.369; Fig. 4c). *KRAS*, *LRP1B*, and *TP53* (bortezomib-insensitive cell lines) and *CREBBP*, *KMT2C*, *KMT2D*, and *TP53* (bortezomib-sensitive cell lines) were identified as commonly mutated cancer drivers in ≥ 5 cell lines (Supplementary Fig. 2a-b). Taken together, mutations were commonly occurred in all cell lines, but mutation rates for the *MUC12*, *PCDH15*, *RYR1* and *SPTA1* genes were significantly higher in the bortezomib-insensitive group.

### Gene expression analysis reveals 33 genes predicted to be involved in bortezomib insensitivity

**Figure 5:**
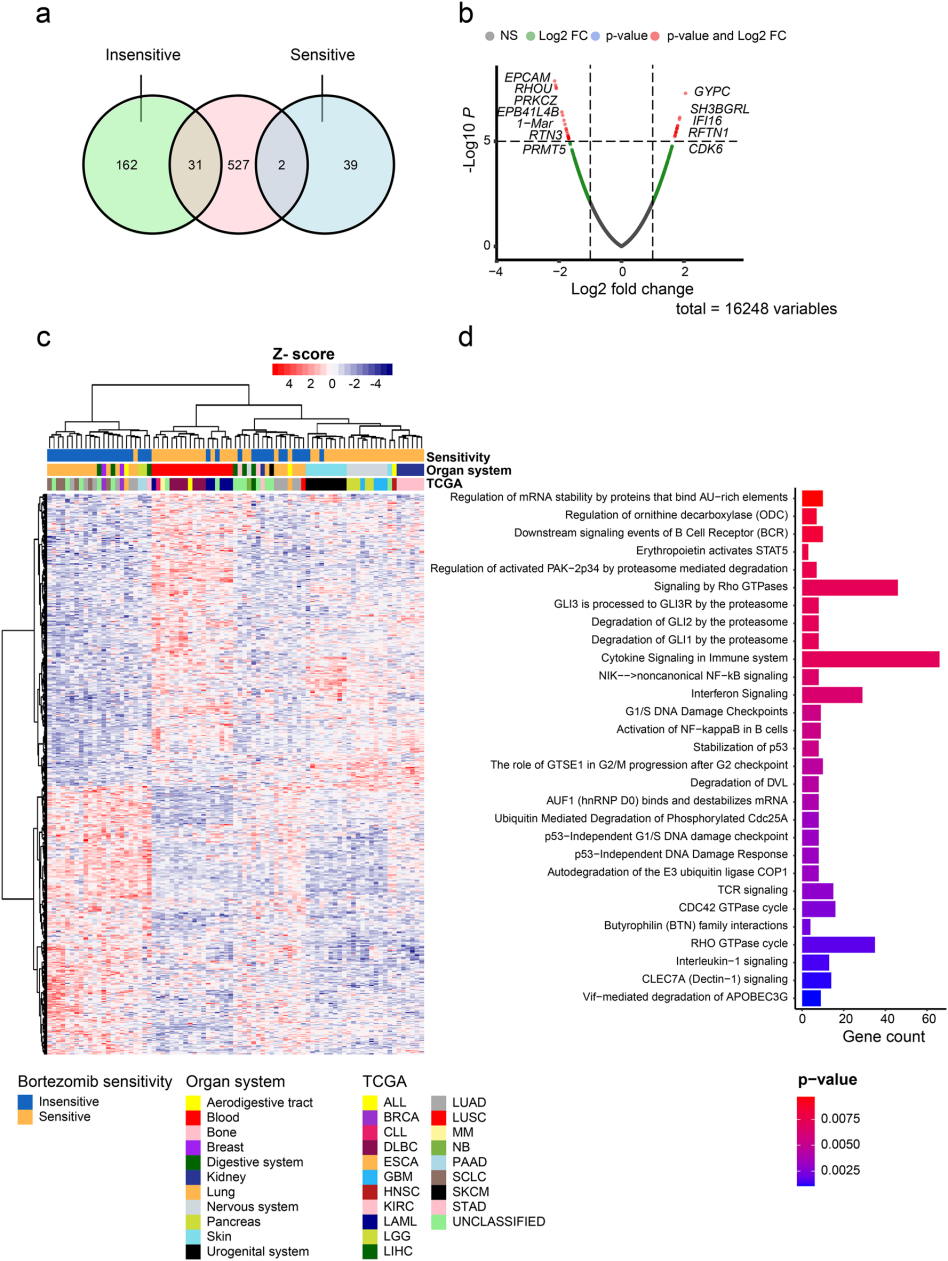
The effect of bortezomib treatment on gene dysregulation in the two sensitivity groups. (**a**) Venn diagram illustrating the number of dysregulated genes in > 20% of cell lines in each sensitivity group. The pink circle (middle) shows the number of dysregulated genes (n = 560) identified using “limma” R package, while the green (left) and blue (right) circles represent genes identified by Sanger COSMIC Cell Lines Project that were associated with bortezomib-insensitivity (n = 193) and sensitivity (n = 41), respectively. Of genes identified in the Sanger COSMIC Cell Lines Project, 31 genes out of 193 and 2 genes out of 41 were found among the 560 genes identified by limma. (**b**) Volcano plot showing the 560 most dysregulated (up or down regulated) genes identified using limma R package. (**c**) Heatmap illustrating hierarchical clustering for expression of the 560 genes identified using limma with Manhattan distance metric and Ward’s minimum variance method (Ward.D2). (**d**) Gene Ontology and Reactome enrichment analysis for the 560 dysregulated genes between the bortezomib sensitivity groups.

We then examined gene expression patterns in untreated bortezomib-sensitive (n = 46) and bortezomib-insensitive (n = 37) cell lines using transcriptomics data for 970 cell lines from the Sanger COSMIC Cell Lines Project. Consequently, 41 (27 over- and 14 underexpressed) and 193 (127 over- and 66 underexpressed) dysregulated genes were identified in > 20% of the cell lines in the bortezomib-sensitive and bortezomib-insensitive groups, respectively (Fig. 5a and Supplementary Fig. 3a,b). Genes associated with bortezomib-sensitivity were found to be involved in transmembrane transport of small molecules, metabolism, chromatin organization, while genes associated with bortezomib-insensitivity played a role in the metabolism of proteins, signal transduction, and the cell cycle. Using transcriptomic and survival data from UCSC Xena Browser, the influence of 220/234 genes (missing data for 14 genes) on survival was investigated in 589 bortezomib-treated multiple myeloma (MM) patients from the MMRF-COMMPASS study ^23^ (Supplementary Table 5). Principal component analysis (PCA) of the transcriptomic data revealed two distinct clusters containing a mix of MM stages in both groups (Supplementary Fig. 4). Furthermore, hierarchical clustering showed that the majority of genes were uniformly expressed in all patients, while 7/220 genes (*DDX3Y*, *EIF1AY*, *RPS4Y1*, *SMCY*, *USP9Y*, *UTY*, and *ZFY*) showed aberrant gene expression patterns in the two main clusters of MM patients (Supplementary Fig. 5). Kaplan–Meier analysis revealed significantly more unfavorable overall survival for MM patients with low expression of the seven genes (Supplementary Fig. 6).

Moreover, differential expression (DE) analysis using limma identified 560 DE genes between bortezomib-sensitive and bortezomib-insensitive cell lines, of which 292 were overexpressed and 268 were underexpressed (Fig. 5b and Supplementary Table 6). However, 33/560 genes were identified by both Sanger and limma (*ALDH18A1*, *ATAD2*, *ATP9A*, *ATXN7*, *BRMS1L*, *CCNH*, *CDC42EP2*, *CENPX_ENST00000580435*, *FBXO21*, *FKBP3*, *HAUS6*, *IGFBPL1*, *LIFR*, *LRTOMT*, *MBIP*, *NDUFB9*, *NINL*, *OXSR1*, *P2RY2*, *PARP2*, *POFUT2*, *PRKG2*, *PUF60*, *SCRIB*, *SHQ1*, *SLC2A4RG*, *SLC45A4*, *TMEM45B*, *TOX4*, *UGDH*, *USP4*, *WDR36*, *WDYHV1*; Fig. 5a). Hierarchical clustering of the expression patterns for the 560 genes demonstrated relatively good clustering depending on bortezomib sensitivity, tissue type, and TCGA cancer type (Fig. 5c). More specifically, bortezomib-sensitive organ systems (blood, skin, nervous system, and kidney) frequently clustered together, while cell lines in the bortezomib-insensitive organ systems (digestive, lung and urogenital) clustered differently. Furthermore, the 560 genes were found to play a central role in the immune system and cell cycle (Fig. 5d). Using the TIMER2.0 database, we then assessed the relationship between immune infiltrates (B cells, CD4 + T cells, CD8 + T cells, myeloid dendritic cells, macrophages, and neutrophils) and the expression of the four genes (*BTN3A1*, *MAP3K3*, *PSMB11*, and *UBA7*) involved in immune response (Supplementary Fig. 7). We were thereby able to show a positive correlation between *BTN3A1*, *MAP3K3*, and *UBA7* expression and the majority of immune infiltrates (B cells, CD4 + T cells, CD8 + T cells, macrophages, and neutrophils) in most cancer types, and a negative correlation with myeloid dendritic cells. However, *PSMB11* expression was only weakly correlated with a few cancer types.

Using two different approaches, we were therefore able to identify 33 concordant genes associated with bortezomib sensitivity. We investigated the gene expression patterns for 32/33 concordant genes (one gene missing data) and their influence on overall survival in the Pan-cancer cohort (21 cancer types) using KM plotter. *ATAD2*, *BRMS1L*, *PUF60*, *SHQ1*, and *USP4* were found to influence overall survival in 15, 9, 13, 13, and 12 of the 21 cancer types, respectively (Supplementary Fig. 8).

## Discussion

In the current study, we evaluate bortezomib sensitivity in 860 cancer cell lines representing 30 cancer types and identify key genomic and transcriptomic features of bortezomib-sensitive and bortezomib-insensitive cells. Mutational events have previously been correlated with therapy resistance^24^. Although MM and mantel cell lymphoma are routinely treated with bortezomib, this study reveals that other hematological cancers (ALL, CLL, DLBC, LAML, LCML), as well as cancers of the kidney, nervous system, and skin may also benefit from treatment with bortezomib^25^. Heterogeneity in response to treatment within organ systems (e.g. lung) was also observed, where several cell lines from the same organ system were classified as bortezomib-sensitive while others were classified as bortezomib-insensitive. When setting up a drug screen, this diversity in sensitivity to treatment must be considered and a wide range of bortezomib concentrations must be used to be able to determine the IC50 and AUC values ​​for all tested samples. The manufacturer Cell Signaling Technology recommends using 1–1000 nM of bortezomib in in vitro studies. However, in our analysis we could not determine the IC50 after 24 h of bortezomib exposure in e.g. breast cancer cell lines (BT-474 and ZR-75–30) even when using up to 10,000 nM. When exposing the cells for 72h with bortezomib, we were able to determine the IC50 values for these cell lines. Therefore, the dose range that should be used when setting up a drug screen for bortezomib will depend on the cell type that will be examined.

Nevertheless, lung cancers were primarily insensitive to treatment with bortezomib. Our in vitro studies confirmed that although bortezomib inhibited the proteasome in all four cell lines (A-172, A-375, BT-474, and ZR-75–30), treatment-induced apoptotic cell death and G2/M cell cycle arrest were primarily shown in A-172 glioblastoma and A-375 melanoma cells. A significant increase in the proportion of necrotic-late apoptotic cells was observed after 72h with 50 nM bortezomib in both A-172 and A-375 cell lines. The breast cancer cell lines (BT-474 and ZR-75–30) were tolerant to up to 100 nM bortezomib with no G2/M arrest or apoptosis observed following 24h or 72h drug exposure. Although 24h treatment with bortezomib is more biologically relevant since its terminal half-life in humans is approximately 15h, we also included the 72h time point in the study in order to evaluate the long-term effect of treatment with bortezomib^26,27^.

The impact of treatment with bortezomib was revealed when about 40% of the β5 catalytic site was inhibited at a concentration of 100 nM, leading to apoptosis in A-172 and A-375 cells. These findings not only indicate that these cells were sensitive to proteasome inhibition, but that a functional proteasome is important for their survival. Previously, other researchers have also found that the A-375 cell line is sensitive to bortezomib by affecting proliferation and cell survival^28^. We also determined that bortezomib induced G2/M phase arrest before the cells underwent apoptosis. G2/M arrest induced by bortezomib is a common event, which has also been described in glioblastoma cancer cells^29^.

Among the 19 genes (*ANKRD36C*, *FSIP2*, *HERC2*, *HMCN1*, *MGAM*, *MUC12*, *MUC16*, *PCDH15*, *PDE4DIP*, *PTPRN2*, *RIMS2*, *RYR1-3*, *SPTA1*, *TP53*, *TRRAP*, *USH2A*, and *WDFY4*) determined to be significantly more mutated in bortezomib-insensitive cell lines, only *TP53* has previously been described to be involved in treatment resistance^30^. Furthermore, we identified 33 dysregulated genes (e.g. *ALDH18A1*, *ATAD2*, *FBXO21*, *LIFR*) potentially involved in therapy resistance, as shown in other studies^31–33^. We demonstrated that bortezomib-insensitive cell lines have significantly higher mutation frequencies than bortezomib-sensitive cells, indicating that the increased mutation frequency confers a reduction in sensitivity to bortezomib treatment. However, mutations in the 19 genes were more recurrent in bortezomib-insensitive cells, thereby suggesting a link with bortezomib resistance. *TP53* mutations have previously been correlated with chemo/radio-resistance^30^. Using the CCLE mutation dataset containing 27/38 bortezomib-insensitive and 22/49 bortezomib-sensitive cancer cell lines, we were able to show consistently elevated mutation frequencies for 18/19 genes (not *PDE4DIP*) in bortezomib-insensitive cell lines compared to bortezomib-sensitive cell lines. However, only 5/19 genes (*MUC12*, *MUC16*, *RIMS2*, *RYR2*, and *SPTA1*) showed significantly higher mutation rates in bortezomib-insensitive cell lines. The fact that we could only validate 5 of the 19 genes in the CCLE dataset could possibly be due to the relatively high number of cell lines from the GDSC dataset that were missing in the CCLE dataset. However, the clinical significance of these mutated genes needs to be investigated further to determine their potential contribution to bortezomib resistance. Interestingly, mutation frequency in cancer drivers was similar in both sensitivity groups.

Using Sanger expression data, we identified a total of 234 genes involved in bortezomib sensitivity across cancer types. Interestingly, external validation showed that low expression of seven genes (*DDX3Y*, *EIF1AY*, *RPS4Y1*, *SMCY*, *USP9Y*, *UTY*, and *ZFY*) had an adverse effect on survival for MM patients and three genes (*BTN3A1*, *MAP3K3*, and *UBA7*) were correlated with immune infiltrates in multiple cancer types. However, by performing the transcriptomics analysis in two steps, we were able to identify 33 differentially expressed genes (e.g. *ALDH18A1*, *ATAD2*, *FBXO21*, *LIFR*) between the bortezomib-sensitivity groups. Among these genes, overexpressed *LIFR* has previously been reported as an important cancer-related gene involved in tumor growth, metastasis, and therapy resistance^31^. The vast majority of these genes (31/33) were also found in at least 20% of the bortezomib-insensitive cell lines using Sanger gene expression data. Survival analysis showed that several of the dysregulated genes (e.g. *ATAD2*, *BRMS1L*, *PUF60*, *SHQ1*, and *USP4*) have an impact on prognosis in multiple cancer types, thereby further highlighting the clinical significance of aberrant gene expression patterns on patient survival. These putative biomarkers need further evaluation to determine their role in treatment sensitivity and clinical utility during cancer treatment decision-making. Furthermore, hierarchical clustering demonstrated that cell lines derived from bortezomib-sensitive organ systems (e.g. blood, kidney, lung, nervous system, and skin) clustered together due to similar transcriptomic profiles even if some of the cancer types were bortezomib-insensitive. However, bortezomib-insensitive organ systems also displayed transcriptomic heterogeneity as some cell lines from the same organ system clustered together while others did not. These findings may be due to inherent differences within specific cancer types, e.g. breast cancer can be stratified into several subtypes (e.g. Luminal, HER2 + , and triple-negative) with varying clinical and biological features. According to the LNIC50 thresholds set in the current study, all nine bortezomib-sensitive breast cancer cell lines were estrogen receptor-negative (5/9 triple-negative), whereas all four bortezomib-insensitive cell lines were estrogen receptor-positive. In previous work, we showed that triple-negative breast cancer cell lines were more sensitive to treatment with bortezomib than estrogen receptor-positive cells^34^.

Although the current study investigated up to 1,000 cancer cell lines representing various cancer types and organ systems, some cancer types were still underrepresented, e.g. lung cancer was represented by 163 cell lines while pancreas cancer was only represented by 14 cell lines. Moreover, the GDSC datasets interpreted treatment sensitivity using cell viability metrics like IC50 and AUC values. IC50 is known to be influenced by cellular proliferation rates that can differ significantly between laboratories, but these factors could be remedied using e.g. growth rate metrics (e.g. GR50) which normalizes growth rate inhibition^34^. To circumvent this problem, we used data for both IC50 and AUC values to set “sensitivity” thresholds that could stratify the 860 cancer cell lines into two sensitivity groups (bortezomib-sensitive and bortezomib-insensitive).

In conclusion, our in silico drug repositioning approach revealed a number of cancer types, besides multiple myeloma and mantel cell lymphoma, which may be suitable for treatment with bortezomib. We also showed that bortezomib is a multifaceted and potent drug for several solid tumors from multiple organ systems. By integrating genomic and transcriptomic profiles with corresponding sensitivity data for bortezomib, we were able to identify mutated (e.g. *MUC12*, *PCDH15*, *RYR1* and *SPTA1*) and dysregulated genes (e.g. *ALDH18A1*, *ATAD2*, *FBXO21*, *LIFR*), thereby warranting further evaluation for their role in resistance to bortezomib. Due to their consistent expression patterns in bortezomib-treated samples, these genes may be suitable putative predictive biomarkers to ensure treatment precision for bortezomib and other proteasome inhibitors. However, future studies showing the effect of treatment with bortezomib using animal models and patient-derived samples are warranted.

## Methods

### Drug sensitivity data

**Figure 6:**
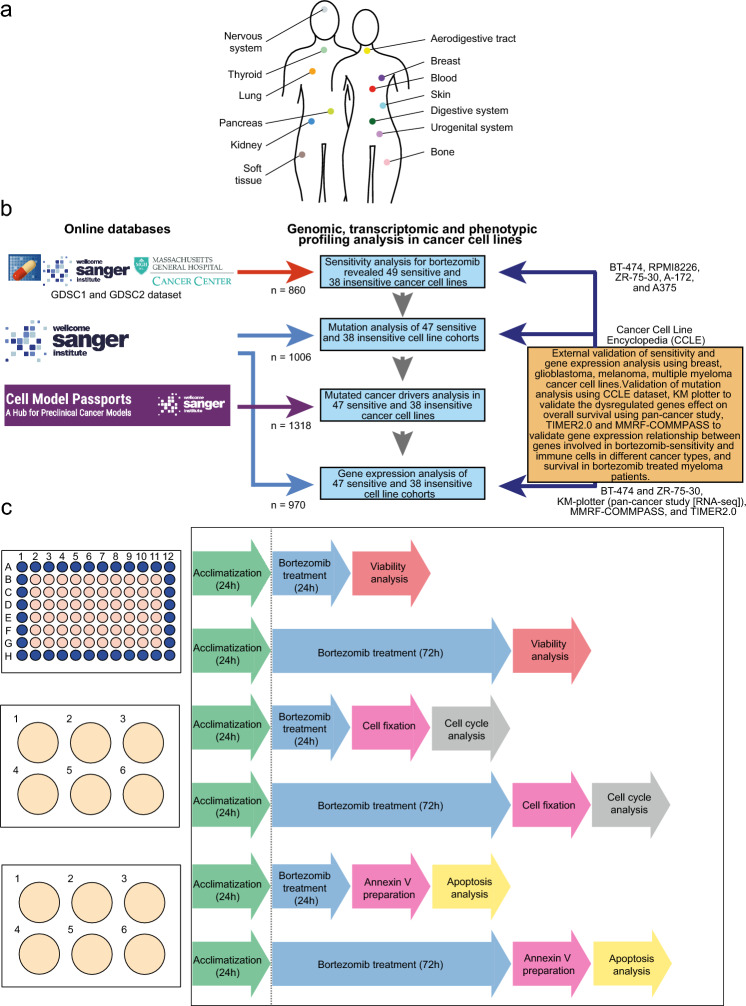
Flowchart describing the workflow and study design. (**a**) Schematic image illustrating the 13 organ systems for the cancer cell lines included in the study. (**b**) Data sources and cell line availability in the different analyses. External validation was performed using cancer cell lines derived from breast, glioblastoma, melanoma, and multiple myeloma cancer, as well as CCLE mutation data to confirm the mutation frequency of 19 genes. KM-plotter was used to explore the influence of 32 of the 33 dysregulated genes on survival in patient samples representing 21 cancer types (pan-cancer). TIMER2.0 was used to validate gene expression relationship between genes involved in bortezomib-sensitivity and immune cells in different cancer types and genes involved in bortezomib-sensitivity and outcome in myeloma patients using MMRF-COMMPASS study. (**c**) Flowchart depicting the study design for the drug sensitivity screen, cell cycle distribution analysis, and apoptosis analysis using Annexin V.

To explore cancer cell line sensitivity to proteasome inhibitors, two publicly available drug sensitivity datasets containing half-maximum inhibitory concentration (IC50) and area under the curve (AUC) values for bortezomib were retrieved from the Genomics of Drug Sensitivity in Cancer (GDSC) database^35–37^. The two datasets consisted of 791 (GDSC1; Sanger) and 756 (GDSC2; Massachusetts General Hospital, MGH) cancer cell lines originating from 13 organ systems (aerodigestive tract, blood, bone, breast, digestive system, kidney, lung, nervous system, pancreas, skin, soft tissue, thyroid, and urogenital system; Fig. 6a), 30 cancer types, and 11 unclassified groups. The datasets were then merged into a single dataset and cell lines existing in both datasets were removed from the GDSC2 dataset. In total, 860 cell lines were included in the study (Supplementary Table 1). Then, logarithmic IC50 (LNIC50) and AUC values were used to stratify the data into two bortezomib sensitivity groups (bortezomib-sensitive and bortezomib-insensitive). The sensitivity groups were classified as follows: a) bortezomib-sensitive for LNIC50 < -6 and AUC < 0.6 and b) bortezomib-insensitive for LNIC50 > -3 and AUC > 0.9 (Table 1 and Supplementary Fig. 7a–f). To be classified in a specific sensitivity group, each cell line needed to be classified as “bortezomib-sensitive” or “bortezomib-insensitive” with both LNIC50 and AUC values. The study design and workflow are shown in Fig. 6b.

**Table 1:** Bortezomib-insensitive and bortezomib-sensitive cancer cell lines (GDSC1 and GDSC2 datasets).

Cell line	TCGA abbreviation	TCGA	Organ system	Organ subtype	Sensitivity group	IC50 (µM)	AUC	Dataset	LNIC50	Sensitivity dataset (GDSC1/GDSC2)	Mutation dataset (Sanger COSMIC)	Gene expression dataset (Sanger COSMIC)	Cancer driver dataset (cell model passports/Sanger institute)
BE-13	ALL	-	blood	lymphoblastic leukemia	Bortezomib sensitive	0.000957	0.55649	GDSC2	-6.951707167	yes	yes	yes	yes
MY-M12	ALL	-	blood	leukemia	Bortezomib sensitive	0.000829	0.43641	GDSC1	-7.095290403	yes	yes	no	yes
BT-474	BRCA	Breast invasive carcinoma	breast	breast	Bortezomib insensitive	0.08264	0.90835	GDSC2	-2.493261454	yes	yes	yes	yes
ZR-75–30	BRCA	Breast invasive carcinoma	breast	breast	Bortezomib insensitive	0.209801	0.93195	GDSC2	-1.561595817	yes	yes	yes	yes
EHEB	CLL	-	blood	lymphoid neoplasm other	Bortezomib sensitive	0.002302	0.58256	GDSC1	-6.073976969	yes	yes	no	no
DB	DLBC	Lymphoid Neoplasm Diffuse Large B-cell Lymphoma	blood	B cell lymphoma	Bortezomib sensitive	0.001278	0.59362	GDSC2	-6.662458923	yes	yes	yes	yes
Farage	DLBC	Lymphoid Neoplasm Diffuse Large B-cell Lymphoma	blood	B cell lymphoma	Bortezomib sensitive	0.00088	0.43951	GDSC1	-7.03558865	yes	yes	yes	yes
HT	DLBC	Lymphoid Neoplasm Diffuse Large B-cell Lymphoma	blood	B cell lymphoma	Bortezomib sensitive	0.002115	0.5853	GDSC1	-6.158700466	yes	yes	no	yes
JM1	DLBC	Lymphoid Neoplasm Diffuse Large B-cell Lymphoma	blood	B cell lymphoma	Bortezomib sensitive	0.000713	0.40372	GDSC1	-7.246029138	yes	yes	yes	yes
NU-DUL-1	DLBC	Lymphoid Neoplasm Diffuse Large B-cell Lymphoma	blood	B cell lymphoma	Bortezomib sensitive	0.001581	0.54082	GDSC1	-6.449697721	yes	yes	yes	yes
OCI-LY-19	DLBC	Lymphoid Neoplasm Diffuse Large B-cell Lymphoma	blood	B cell lymphoma	Bortezomib sensitive	0.001389	0.51763	GDSC1	-6.579171215	yes	yes	yes	yes
SU-DHL-16	DLBC	Lymphoid Neoplasm Diffuse Large B-cell Lymphoma	blood	B cell lymphoma	Bortezomib sensitive	0.000897	0.44052	GDSC1	-7.016454696	yes	yes	yes	yes
SU-DHL-4	DLBC	Lymphoid Neoplasm Diffuse Large B-cell Lymphoma	blood	B cell lymphoma	Bortezomib sensitive	0.000903	0.44133	GDSC1	-7.009788005	yes	yes	yes	yes
COLO-680N	ESCA	Esophageal carcinoma	aero digestive tract	oesophagus	Bortezomib insensitive	0.390167	0.95302	GDSC2	-0.941180426	yes	yes	yes	yes
TE-10	ESCA	Esophageal carcinoma	aero digestive tract	oesophagus	Bortezomib sensitive	0.000693	0.40846	GDSC1	-7.274480559	yes	yes	yes	yes
D-247MG	GBM	Glioblastoma multiforme	nervous system	glioma	Bortezomib sensitive	0.000469	0.36343	GDSC1	-7.66490779	yes	yes	yes	yes
D-542MG	GBM	Glioblastoma multiforme	nervous system	glioma	Bortezomib sensitive	0.000363	0.32261	GDSC1	-7.921107724	yes	yes	yes	yes
SF126	GBM	Glioblastoma multiforme	nervous system	glioma	Bortezomib sensitive	0.000673	0.40408	GDSC1	-7.303765228	yes	yes	yes	yes
SNB75	GBM	Glioblastoma multiforme	nervous system	glioma	Bortezomib sensitive	0.001342	0.5098	GDSC1	-6.61359424	yes	yes	yes	yes
BB30-HNC	HNSC	Head and Neck squamous cell carcinoma	aero digestive tract	head and neck	Bortezomib sensitive	0.000687	0.40531	GDSC1	-7.283176266	yes	yes	yes	yes
SN12C	KIRC	Kidney renal clear cell carcinoma	kidney	kidney	Bortezomib insensitive	0.051792	0.90162	GDSC2	-2.960519582	yes	yes	yes	yes
A704	KIRC	Kidney renal clear cell carcinoma	kidney	kidney	Bortezomib sensitive	0.001266	0.5884	GDSC2	-6.671892955	yes	yes	yes	yes
BB65-RCC	KIRC	Kidney renal clear cell carcinoma	kidney	kidney	Bortezomib sensitive	0.000294	0.29811	GDSC1	-8.131930791	yes	yes	yes	yes
HA7-RCC	KIRC	Kidney renal clear cell carcinoma	kidney	kidney	Bortezomib sensitive	0.001159	0.48867	GDSC1	-6.760197715	yes	yes	yes	yes
LB1047-RCC	KIRC	Kidney renal clear cell carcinoma	kidney	kidney	Bortezomib sensitive	0.002151	0.58133	GDSC1	-6.141822429	yes	yes	yes	yes
LB2241-RCC	KIRC	Kidney renal clear cell carcinoma	kidney	kidney	Bortezomib sensitive	0.000969	0.46139	GDSC1	-6.939245946	yes	yes	yes	yes
RXF393	KIRC	Kidney renal clear cell carcinoma	kidney	kidney	Bortezomib sensitive	0.000451	0.36731	GDSC1	-7.704043218	yes	yes	yes	yes
THP-1	LAML	Acute Myeloid Leukemia	blood	acute myeloid leukaemia	Bortezomib insensitive	0.055733	0.96162	GDSC1	-2.887182848	yes	yes	yes	yes
CMK	LAML	Acute Myeloid Leukemia	blood	acute myeloid leukaemia	Bortezomib sensitive	0.001275	0.50338	GDSC1	-6.6648091	yes	yes	yes	yes
HEL	LAML	Acute Myeloid Leukemia	blood	acute myeloid leukaemia	Bortezomib sensitive	0.001552	0.53731	GDSC1	-6.468210857	yes	yes	yes	yes
OCI-AML5	LAML	Acute Myeloid Leukemia	blood	acute myeloid leukaemia	Bortezomib sensitive	0.001382	0.51672	GDSC1	-6.584223554	yes	yes	yes	yes
OCI-M1	LAML	Acute Myeloid Leukemia	blood	acute myeloid leukaemia	Bortezomib sensitive	0.000874	0.54506	GDSC2	-7.042430182	yes	yes	yes	yes
QIMR-WIL	LAML	Acute Myeloid Leukemia	blood	acute myeloid leukaemia	Bortezomib sensitive	0.002122	0.58368	GDSC1	-6.155396239	yes	yes	no	no
D-336MG	LGG	Brain Lower Grade Glioma	nervous system	glioma	Bortezomib sensitive	0.000595	0.39036	GDSC1	-7.426949152	yes	yes	yes	yes
KNS-42	LGG	Brain Lower Grade Glioma	nervous system	glioma	Bortezomib sensitive	0.000329	0.30692	GDSC1	-8.019452807	yes	yes	yes	yes
KNS-81-FD	LGG	Brain Lower Grade Glioma	nervous system	glioma	Bortezomib sensitive	0.001079	0.4805	GDSC1	-6.831720593	yes	yes	yes	yes
no-10	LGG	Brain Lower Grade Glioma	nervous system	glioma	Bortezomib sensitive	0.000213	0.25458	GDSC1	-8.454218392	yes	yes	yes	yes
no-11	LGG	Brain Lower Grade Glioma	nervous system	glioma	Bortezomib sensitive	0.00161	0.53575	GDSC1	-6.4315211	yes	yes	yes	yes
JHH-6	LIHC	Liver hepatocellular carcinoma	digestive system	liver	Bortezomib insensitive	0.257712	0.94834	GDSC2	-1.355912597	yes	yes	yes	yes
NCI-H1435	LUAD	Lung adenocarcinoma	lung	lung NSCLC adenocarcinoma	Bortezomib insensitive	0.335284	0.99334	GDSC2	-1.092777345	yes	yes	yes	yes
NCI-H1563	LUAD	Lung adenocarcinoma	lung	lung NSCLC adenocarcinoma	Bortezomib insensitive	0.080912	0.92536	GDSC2	-2.514393135	yes	yes	yes	yes
NCI-H1651	LUAD	Lung adenocarcinoma	lung	lung NSCLC adenocarcinoma	Bortezomib insensitive	0.06317	0.94193	GDSC2	-2.761925774	yes	yes	yes	yes
NCI-H1793	LUAD	Lung adenocarcinoma	lung	lung NSCLC adenocarcinoma	Bortezomib insensitive	0.64715	0.93931	GDSC2	-0.435177172	yes	yes	yes	yes
NCI-H1838	LUAD	Lung adenocarcinoma	lung	lung NSCLC adenocarcinoma	Bortezomib insensitive	0.416641	0.93798	GDSC2	-0.875530339	yes	yes	yes	yes
NCI-H1944	LUAD	Lung adenocarcinoma	lung	lung NSCLC adenocarcinoma	Bortezomib insensitive	0.121007	0.92134	GDSC2	-2.111906884	yes	yes	yes	yes
NCI-H2342	LUAD	Lung adenocarcinoma	lung	lung NSCLC adenocarcinoma	Bortezomib insensitive	0.072552	0.92135	GDSC2	-2.623451733	yes	yes	yes	yes
NCI-H441	LUAD	Lung adenocarcinoma	lung	lung NSCLC adenocarcinoma	Bortezomib insensitive	0.842319	0.91685	GDSC2	-0.171596477	yes	yes	yes	yes
VMRC-LCD	LUAD	Lung adenocarcinoma	lung	lung NSCLC adenocarcinoma	Bortezomib insensitive	0.260655	0.96898	GDSC2	-1.344557585	yes	yes	yes	yes
EKVX	LUAD	Lung adenocarcinoma	lung	lung NSCLC adenocarcinoma	Bortezomib sensitive	0.002275	0.56889	GDSC1	-6.085775227	yes	yes	yes	yes
LC-2-ad	LUAD	Lung adenocarcinoma	lung	lung NSCLC adenocarcinoma	Bortezomib sensitive	0.001316	0.50819	GDSC1	-6.633158446	yes	yes	yes	yes
SK-MES-1	LUSC	Lung squamous cell carcinoma	lung	lung NSCLC squamous cell carcinoma	Bortezomib insensitive	0.072725	0.9298	GDSC2	-2.621070075	yes	yes	yes	yes
NCI-H2595	MESO	Mesothelioma	Lung	Lung	Bortezomib insensitive	0.372020619	0.95654	GDSC2	-0.988806	yes	no	no	yes
AMO-1	MM	-	blood	haematopoietic neoplasm other	Bortezomib sensitive	0.000804	0.53315	GDSC2	-7.125911289	yes	yes	yes	yes
LAN-6	NB	-	nervous system	neuroblastoma	Bortezomib sensitive	0.002104	0.57741	GDSC1	-6.163914984	yes	yes	yes	yes
AsPC-1	PAAD	Pancreatic adenocarcinoma	pancreas	pancreas	Bortezomib insensitive	3.530379	0.96507	GDSC2	1.261405231	yes	yes	yes	yes
PANC-03–27	PAAD	Pancreatic adenocarcinoma	pancreas	pancreas	Bortezomib insensitive	0.06161	0.90168	GDSC2	-2.786931084	yes	yes	yes	yes
COR-L88	SCLC	Small cell lung cancer	lung	lung small cell carcinoma	Bortezomib insensitive	0.119125	0.95221	GDSC2	-2.127581917	yes	yes	yes	yes
DMS-79	SCLC	Small cell lung cancer	lung	lung small cell carcinoma	Bortezomib insensitive	0.067533	0.92992	GDSC1	-2.695138912	yes	yes	yes	yes
LU-165	SCLC	Small cell lung cancer	lung	lung small cell carcinoma	Bortezomib insensitive	0.166904	0.94816	GDSC2	-1.790336482	yes	yes	yes	yes
NCI-H187	SCLC	Small cell lung cancer	lung	lung small cell carcinoma	Bortezomib insensitive	0.056995	0.93544	GDSC1	-2.864791734	yes	yes	yes	yes
NCI-H2066	SCLC	Small cell lung cancer	lung	lung small cell carcinoma	Bortezomib insensitive	0.818439	0.96884	GDSC2	-0.200356412	yes	yes	yes	yes
NCI-H211	SCLC	Small cell lung cancer	lung	lung small cell carcinoma	Bortezomib insensitive	0.059485	0.94412	GDSC2	-2.822031099	yes	yes	yes	yes
SK-MEL-1	SKCM	Skin Cutaneous Melanoma	skin	melanoma	Bortezomib insensitive	0.225859	0.95107	GDSC2	-1.487844368	yes	no	yes	yes
SK-MEL-24	SKCM	Skin Cutaneous Melanoma	skin	melanoma	Bortezomib insensitive	0.094184	0.93528	GDSC2	-2.362504963	yes	yes	yes	yes
A101D	SKCM	Skin Cutaneous Melanoma	skin	melanoma	Bortezomib sensitive	0.001033	0.56127	GDSC2	-6.875288089	yes	yes	yes	yes
CP66-MEL	SKCM	Skin Cutaneous Melanoma	skin	melanoma	Bortezomib sensitive	0.000627	0.39546	GDSC1	-7.374564017	yes	yes	yes	yes
LB2518-MEL	SKCM	Skin Cutaneous Melanoma	skin	melanoma	Bortezomib sensitive	0.000526	0.36593	GDSC1	-7.550209345	yes	yes	yes	yes
LB373-MEL-D	SKCM	Skin Cutaneous Melanoma	skin	melanoma	Bortezomib sensitive	0.000529	0.36846	GDSC1	-7.544522126	yes	yes	yes	yes
MMAc-SF	SKCM	Skin Cutaneous Melanoma	skin	melanoma	Bortezomib sensitive	0.000187	0.22905	GDSC1	-8.584401941	yes	yes	no	yes
MZ2-MEL	SKCM	Skin Cutaneous Melanoma	skin	melanoma	Bortezomib sensitive	0.000395	0.33271	GDSC1	-7.836624793	yes	yes	yes	yes
MZ7-mel	SKCM	Skin Cutaneous Melanoma	skin	melanoma	Bortezomib sensitive	0.000867	0.44333	GDSC1	-7.050471581	yes	yes	yes	yes
OCUM-1	STAD	Stomach adenocarcinoma	digestive system	stomach	Bortezomib insensitive	0.183433	0.93649	GDSC2	-1.695905801	yes	yes	yes	yes
SKM-1	UNCLASSIFIED	–	blood	haematopoietic neoplasm other	Bortezomib insensitive	0.054058	0.92609	GDSC1	-2.917697735	yes	yes	yes	yes
EW-13	UNCLASSIFIED	–	bone	ewings sarcoma	Bortezomib insensitive	0.052193	0.91812	GDSC2	-2.952806893	yes	yes	yes	yes
Saos-2	UNCLASSIFIED	–	bone	osteosarcoma	Bortezomib insensitive	0.27205	0.97193	GDSC2	-1.301769406	yes	yes	yes	yes
SCH	UNCLASSIFIED	–	digestive system	stomach	Bortezomib insensitive	0.085445	0.95839	GDSC1	-2.459882385	yes	yes	yes	yes
TGBC24TKB	UNCLASSIFIED	–	digestive system	biliary tract	Bortezomib insensitive	0.10926	0.93223	GDSC2	-2.214024916	yes	yes	yes	yes
LU-99A	UNCLASSIFIED	–	lung	lung NSCLC large cell	Bortezomib insensitive	0.28162	0.9293	GDSC2	-1.267196634	yes	yes	yes	yes
NCI-H1770	UNCLASSIFIED	–	lung	lung NSCLC not specified	Bortezomib insensitive	0.085353	0.96508	GDSC1	-2.460959681	yes	yes	yes	yes
NCI-H727	UNCLASSIFIED	–	lung	lung NSCLC carcinoid	Bortezomib insensitive	0.063839	0.90719	GDSC2	-2.75139099	yes	yes	yes	yes
NCI-H835	UNCLASSIFIED	–	lung	lung NSCLC carcinoid	Bortezomib insensitive	0.060368	0.94937	GDSC2	-2.807296116	yes	yes	yes	yes
RKN	UNCLASSIFIED	–	urogenital system	ovary	Bortezomib insensitive	0.08518	0.91618	GDSC2	-2.462988614	yes	yes	yes	yes
A388	UNCLASSIFIED	–	skin	skin other	Bortezomib sensitive	0.000387	0.33243	GDSC1	-7.857085865	yes	yes	yes	yes
DEL	UNCLASSIFIED	–	blood	lymphoid neoplasm other	Bortezomib sensitive	0.001282	0.59982	GDSC2	-6.65933392	yes	yes	yes	yes
ECC12	UNCLASSIFIED	–	digestive system	stomach	Bortezomib sensitive	0.001495	0.52573	GDSC1	-6.505629072	yes	yes	yes	yes
HuTu-80	UNCLASSIFIED	–	digestive system	digestive system other	Bortezomib sensitive	0.001119	0.48084	GDSC1	-6.79531985	yes	yes	no	yes
NK-92MI	UNCLASSIFIED	–	blood	lymphoid neoplasm other	Bortezomib sensitive	0.000761	0.41714	GDSC1	-7.1808772	yes	yes	no	yes

## Genomics and transcriptomics profiling data

Genomics and transcriptomics data were retrieved from the Sanger COSMIC Cell Line Project (mutation signatures and gene expression profiles for 970 and 1,007 cell lines, respectively)^38^ and Cell Model Passport (cancer driver mutation frequency data for 1,318 cell lines)^39^ to evaluate mutations on the protein level and gene expression patterns. First, we explored the frequency of mutated genes (missing data for NCI-H2595 and SK-MEL-1 cell lines) and cancer-driver genes in the bortezomib-sensitive and bortezomib-insensitive groups (missing data for the EHEB and QIMP-WIL cell lines). Second, a total of 16,248 genes were evaluated for up- or downregulation in the bortezomib-sensitive and bortezomib-insensitive groups by stratifying gene expression levels based on Z-scores (downregulation < -2, upregulation > 2, and normal expression between ≥ -2 and ≤ 2). Only genes over- or underexpressed in > 20% of the cell lines in each sensitivity group were included. The HT, MY-M12, NCI-H2595, and NK-92MI cell lines were removed from the analysis due to missing data. Genes of interest were further investigated for pathway involvement using the Reactome interactive database^40^.

### Cell culture

To validate our findings from the publicly available datasets, we used the human glioblastoma (A-172), melanoma (A-375) and breast cancer (MCF-7) cell lines provided by Martin Johansson, Jonas Nilsson, Julie Grantham (University of Gothenburg), as well as breast cancer (BT-474 and ZR-75–30) and multiple myeloma (RPMI-8226) cell lines that were purchased from The American Type Culture Collection (Fig. 6c). The cell lines were cultured in RPMI 1640 (RPMI-8226 and ZR-75–30 cells) supplemented with 2 mM L-glutamine, 2 g/L D-glucose, and 10% fetal bovine serum (FBS; ThermoFisher Scientific), or Dulbecco Modified Eagle´s Medium (DMEM; A-172, A-375, BT-474, and MCF-7 cells) supplemented with 2 mM L-glutamine, 4 g/L D-glucose, and 10% FBS (ThermoFisher Scientific) and maintained at 37°C in a humidified 5% CO_2_ environment. Cell authentication was performed using the Eurofins Genomics Human Cell Line Authentication service. First, A-172, A-375, BT-474, RPMI-8226, and ZR-75–30 cells were screened for sensitivity to bortezomib, as described elsewhere^34^. In brief, the cells were seeded on 96-well plates at a density ranging between 3.0 × 10^3^ and 7.5 × 10^3^ cells/well depending on proliferation rates and incubated for 24h. The cells were then exposed to bortezomib (Selleckchem; nine tenfold concentrations between 1 and 10,000 nM) and matched DMSO concentration vehicle controls for 24h and 72h. Cell viability was determined using the resazurin cell viability assay and growth rate metrics assessed (IC50 and AUC) with the GRmetrics (version 1.16.0) package^41^ in R/Bioconductor (version 4.0.3) as previously described^34^, and ggplot2 (version 3.3.5) for data visualization^42^. To determine the proteasome activity of the β5 chymotrypsin-like active site following 2h bortezomib exposure, we used Suc-Leu-Leu-Val-Tyr-AMC (Enzo; Cat. BML-P802) at a concentration of 20 µM per well in 96-well plates for A-172, A-375, BT-474, and ZR-75–30 cells and the fluorescence measured (excitation 355 and emission 460) using a Wallac 1420 VICTOR2 microplate reader (Perkin Elmer).

### Annexin V early apoptosis assay and cell cycle analysis

To evaluate apoptotic cell death and cell cycle distribution in bortezomib-treated cell lines (A-172, A-375, BT-474, and ZR-75–30), the cells were first seeded at a density of 5 × 10^5^ (apoptosis) and 1 × 10^6^ (cell cycle) cells/well on 6-well plates and incubated for 24h at 37°C in a humidified 5% CO_2_ environment. After 24h, the medium was discarded and new medium containing bortezomib (1, 10, and 100 nM) was added to treatment wells and corresponding DMSO (drug solvent) to control wells. Additional bortezomib doses (25, 50 and 75 nM) were evaluated for A-172 and A-375 cells due to extremely low cell viability at 100 nM. Following treatment with bortezomib for 24h or 72h, the cells were collected and washed twice with PBS. In total, 1 × 10^5^ cells were further processed using Annexin V-FITC Early Apoptosis Detection Kit (Cat. 6592, Cell Signaling Technology, Danvers, Massachusetts, USA) according to the manufacturer’s instructions and analyzed 10,000 events using a BD Accuri™ C6 personal flow cytometer (BD Biosciences, Franklin Lakes, New Jersey, USA) with the FL1 (Annexin V-FITC) and FL3 channels (propidium iodide, PI; Fig. 6c). Data analysis was performed using FlowJo™ v10.8.1 Software (BD Life Sciences). For cell cycle analysis, the cells were fixed in pre-chilled 70% ethanol and stored at -20°C for at least three days. The cells were then centrifuged at 220xg, washed twice with PBS, and stained using PI/RNase Staining Solution (Cat. 4087, Cell Signaling Technology, Danvers, Massachusetts, USA). Data analysis was performed for 10,000 events using a BD LSRFortessa™ cell analyzer (BD Biosciences, Franklin Lakes, New Jersey, USA) and FlowJo™ (BD Life Sciences; Fig. 6c).

### Quantitative real-time PCR

Total RNA was extracted from untreated BT-474, MCF-7, and ZR-75–30 cells using the RNeasy Lipid Tissue Mini Kit (Qiagen), followed by evaluation of RNA concentration and integrity using Qubit (ThermoFisher Scientific) and TapeStation (Agilent), respectively. Complementary DNA was synthesized with the Superscript III First-Strand Synthesis for qRT-PCR kit (ThermoFisher Scientific). Quantitative real-time PCR (qPCR) was performed using predesigned TaqMan Gene Expression Assays (ThermoFisher Scientific) for *SHARPIN* (HS00229642_m1) and *VPS28* (HS01598026_m1) with a 7500 Fast Real-Time PCR System (Applied Biosystems Inc). The relative gene expression patterns were determined using the ΔCt method after normalizing the data using the geometric mean of three endogenous controls (*HPRT1* [Hs02800695_m1], *PPIA* [Hs99999904_m1], and *PUM1* [HS00472881_m1]).

### External validation

The 19 genes with significantly elevated mutation rates in the bortezomib-insensitive group were validated using the CCLE mutation dataset downloaded from cBioPortal (https://www.cbioportal.org/). Only 27/38 bortezomib-insensitive and 22/49 bortezomib-sensitive cancer cell lines were available in the CCLE mutation dataset. The clinical significance of aberrant gene expression patterns (high or low expression) for 32 of the 33 dysregulated genes (CENPX_ENST00000580435 gene was excluded due to missing data) were evaluated using the KM plotter interactive website to assess the effect of expression on overall survival in the pan-cancer cohort containing 7,389 cancer samples (RNA-seq) derived from 21 cancer types. The default settings were used for KM plotter. Analysis of gene expression patterns for 220 genes (193 genes identified in bortezomib-insensitive cell lines and 41 genes identified in bortezomib-sensitive cell lines; data for 14 genes were missing) linked to survival for 589 MM patients treated with bortezomib from the MMRF-COMMPASS study was performed using clinical data from the UCSC Xena Browser and KM plotter (overall survival). Gene expression was analyzed using DESeq2 (version 1.0.2), followed by hierarchical clustering using pheatmap (version 1.0.12) with Manhattan distance metric and Ward’s minimum variance method (Ward.D2), ggplot2 (version 3.4.4) for data visualization, and summarization of the dataset with tableone (version 0.13.2). TIMER2.0 was used to assess the relationship between immune infiltrates (B cells, CD4 + T cells, CD8 + T cells, myeloid dendritic cells, macrophages, and neutrophils) and the expression of genes associated with immune response.

### Statistical analysis

Statistical analyses were performed in R/Bioconductor (version 4.0.3) and Microsoft Excel 2016/2019. *P* < 0.05 was considered to be statistically significant. Hierarchical clustering of gene expression patterns was performed using the pheatmap R package (version 1.0.12)^43^ and the Manhattan distance metric and Ward’s minimum variance method (Ward.D2). Differentially expressed genes between the sensitivity groups were identified using Limma R package (version 3.50.0)^44^. The mutation frequency between the sensitivity groups was performed using the ggstatsplot R package (version 0.9.1) with ggbetweenstats^45^. Waterfall plots were used to stratify the 50 most frequently mutated genes in each sensitivity group using maftools package (version 2.10.0) and GenVisR package^46,47^. If multiple mutations were detected in the same gene, the most deleterious (mutations that affect the protein to become less functional or non-functional) was considered^48^. A descriptive analysis using one-way ANOVA with the dplyr R package (version 1.0.8) was used to identify significant changes in cell cycle arrest and apoptosis induced by bortezomib^49^.

### Supplementary Information

Supplementary Figure 1.

Supplementary Table 1.

Supplementary Table 2.

Supplementary Table 3.

Supplementary Table 4.

Supplementary Table 5.

Supplementary Table 6.

## Data Availability

All data used in this study are included or referred to within this work.
