# Supplementary material for: Multi-omics analysis identifies repurposing bortezomib in the treatment of kidney-, nervous system-, and hematological cancers
Source: Sci Rep. 2024 Aug 10;14:18576. doi: 10.1038/s41598-024-62339-x (PMC11316778; doi:10.1038/s41598-024-62339-x)
Supplement: Supplementary file 1 — Supplementary Figure 1. [file 41598_2024_62339_MOESM1_ESM.pdf]

Supplementary Figures

Supplementary Fig. 1

a

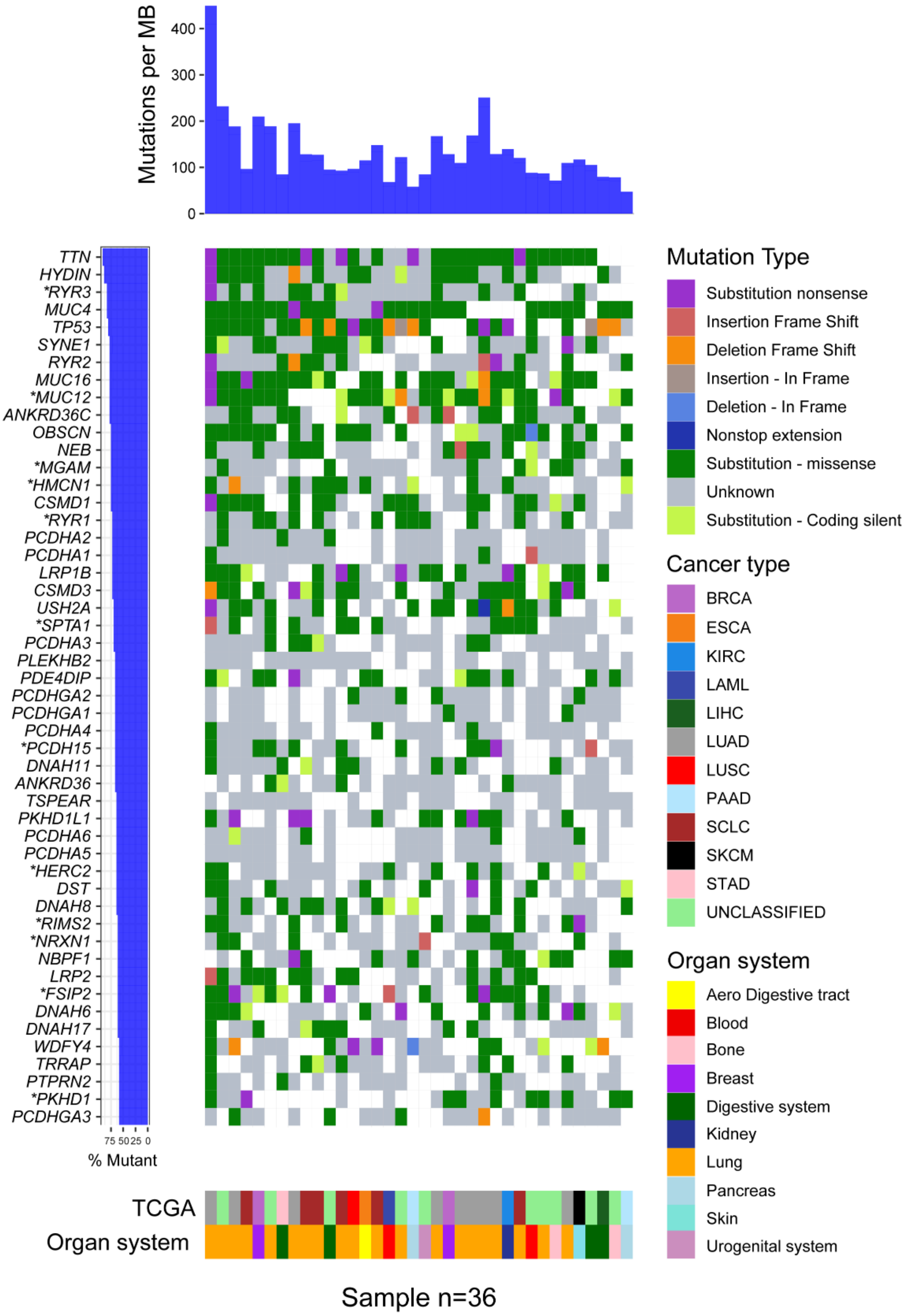

b

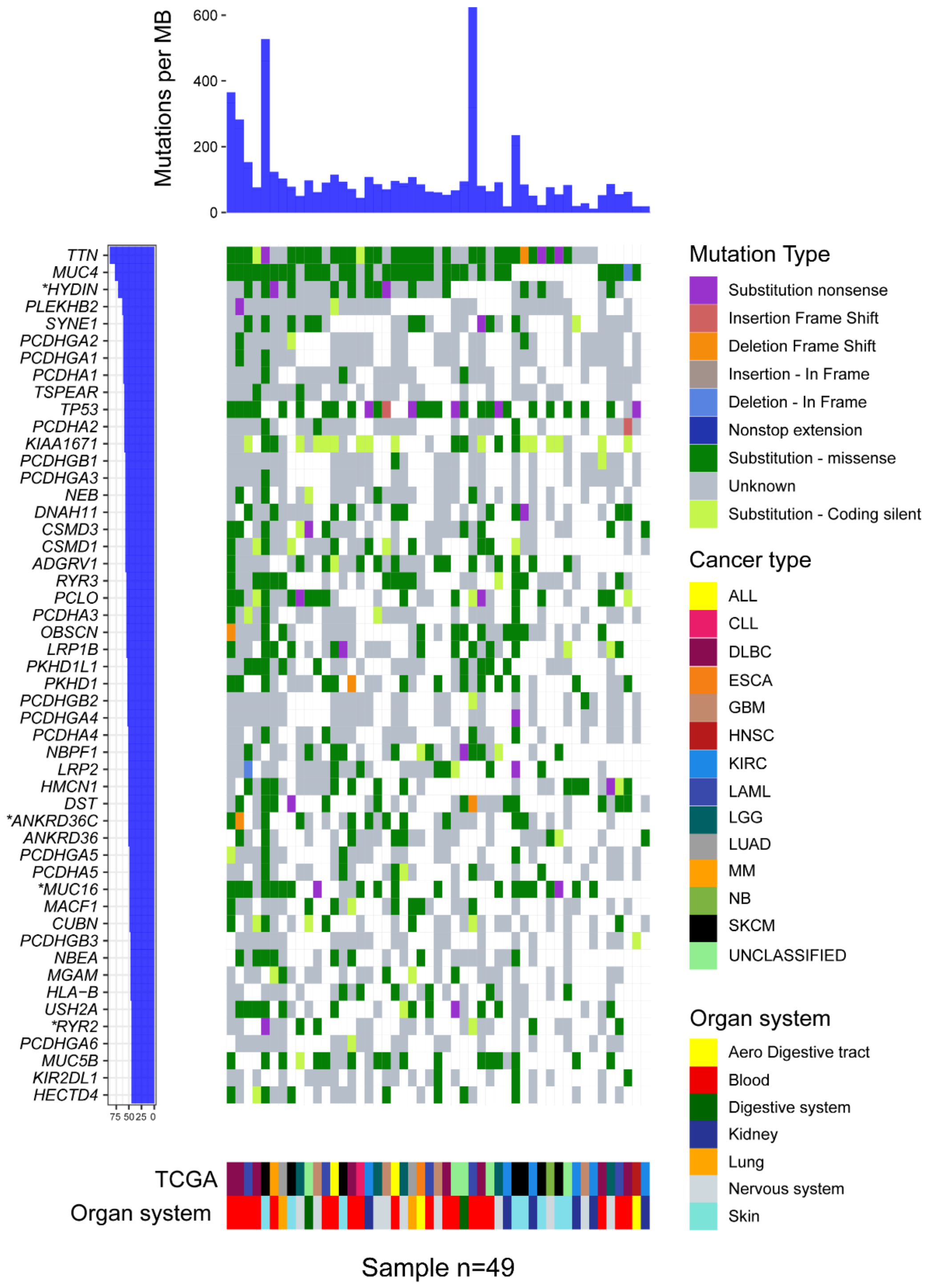

Supplementary Fig. 1. Waterfall plots depicting the 50 most mutated genes in (**a**) bortezomib-insensitive and (**b**) sensitive cancer cell lines.

Supplementary Fig. 2

a

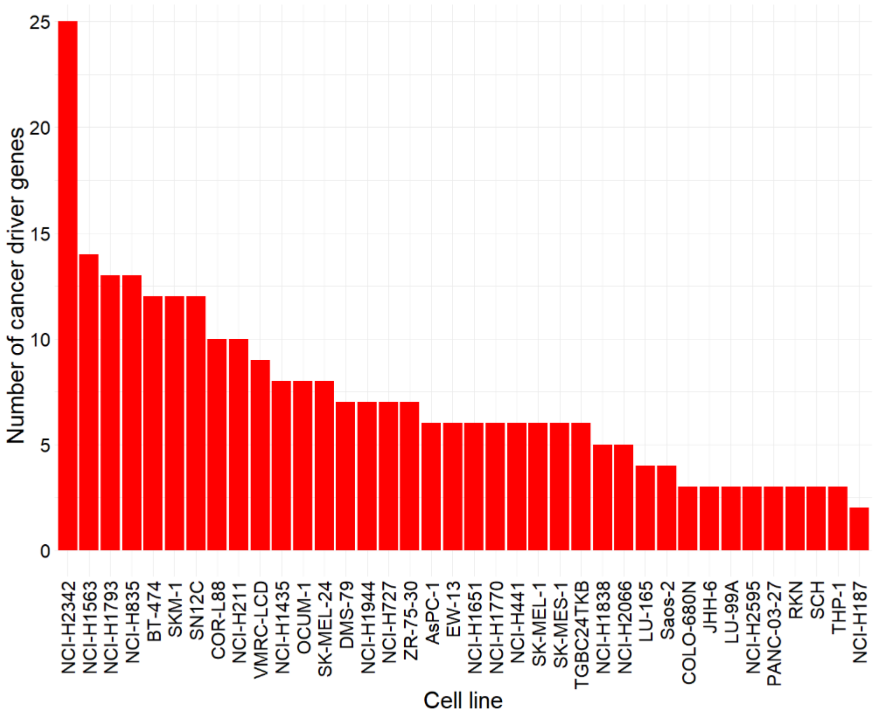

b

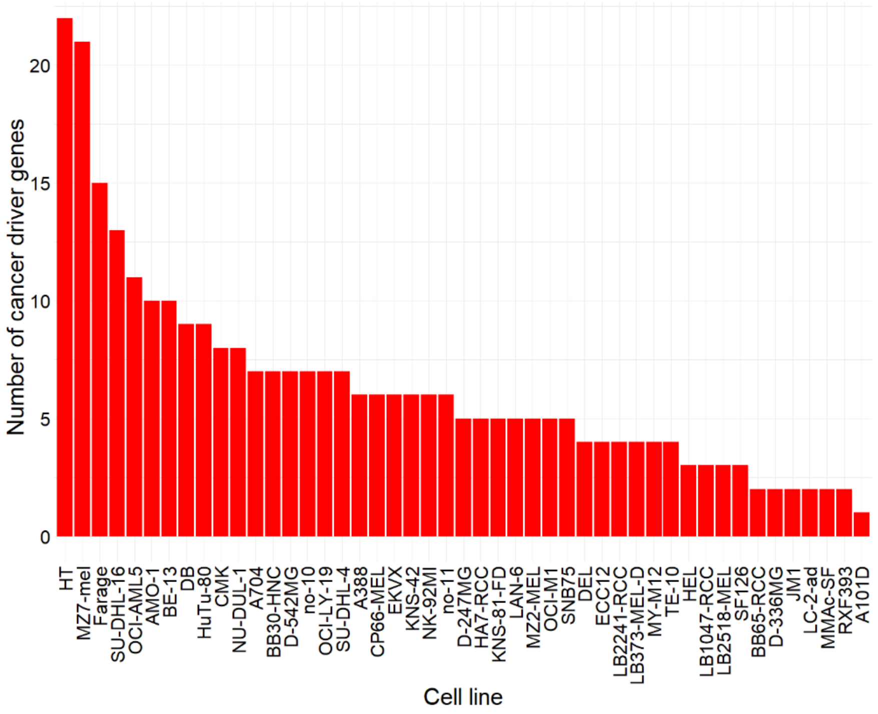

Supplementary Fig. 2. Bar charts showing the number of mutated cancer driver genes in (**a**) bortezomib-insensitive and (**b**) sensitive cancer cell lines.

Supplementary Fig. 3

a

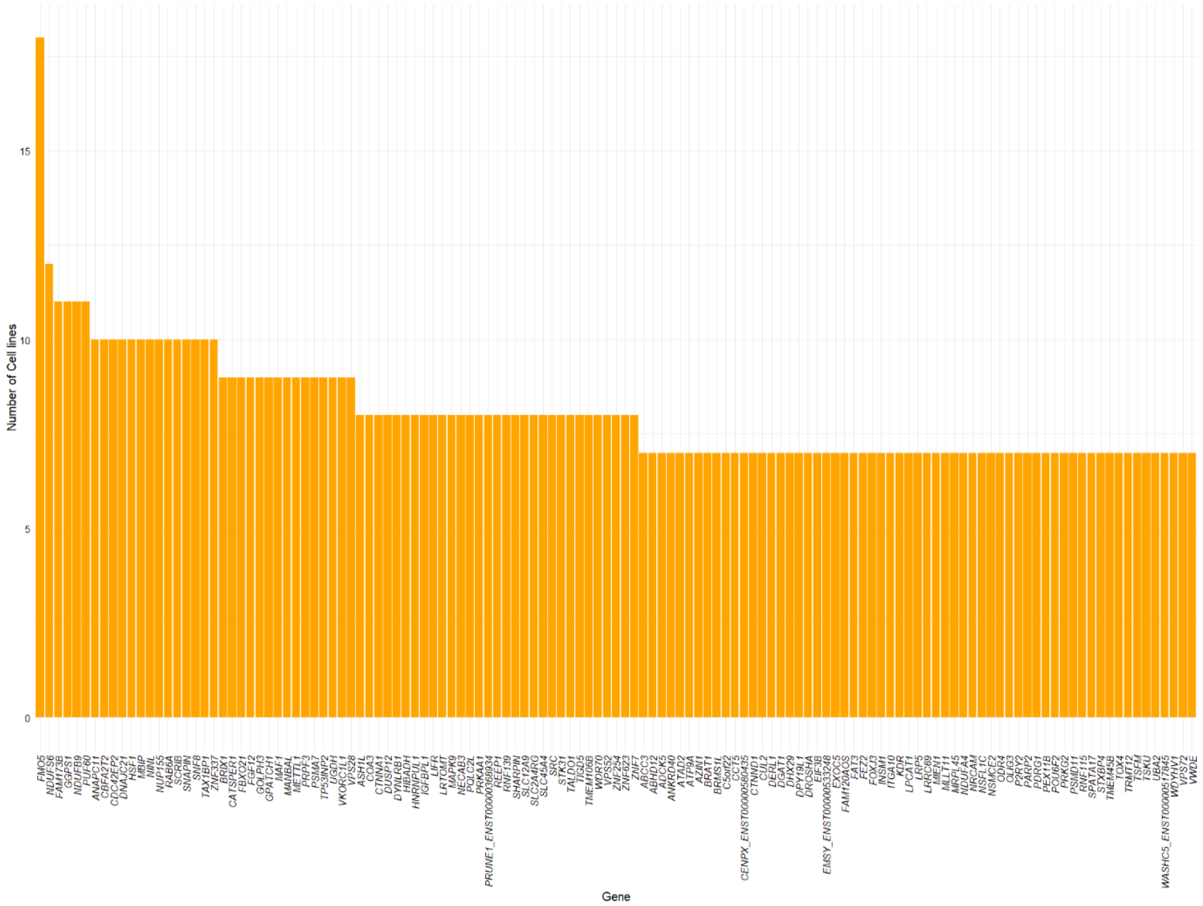

b

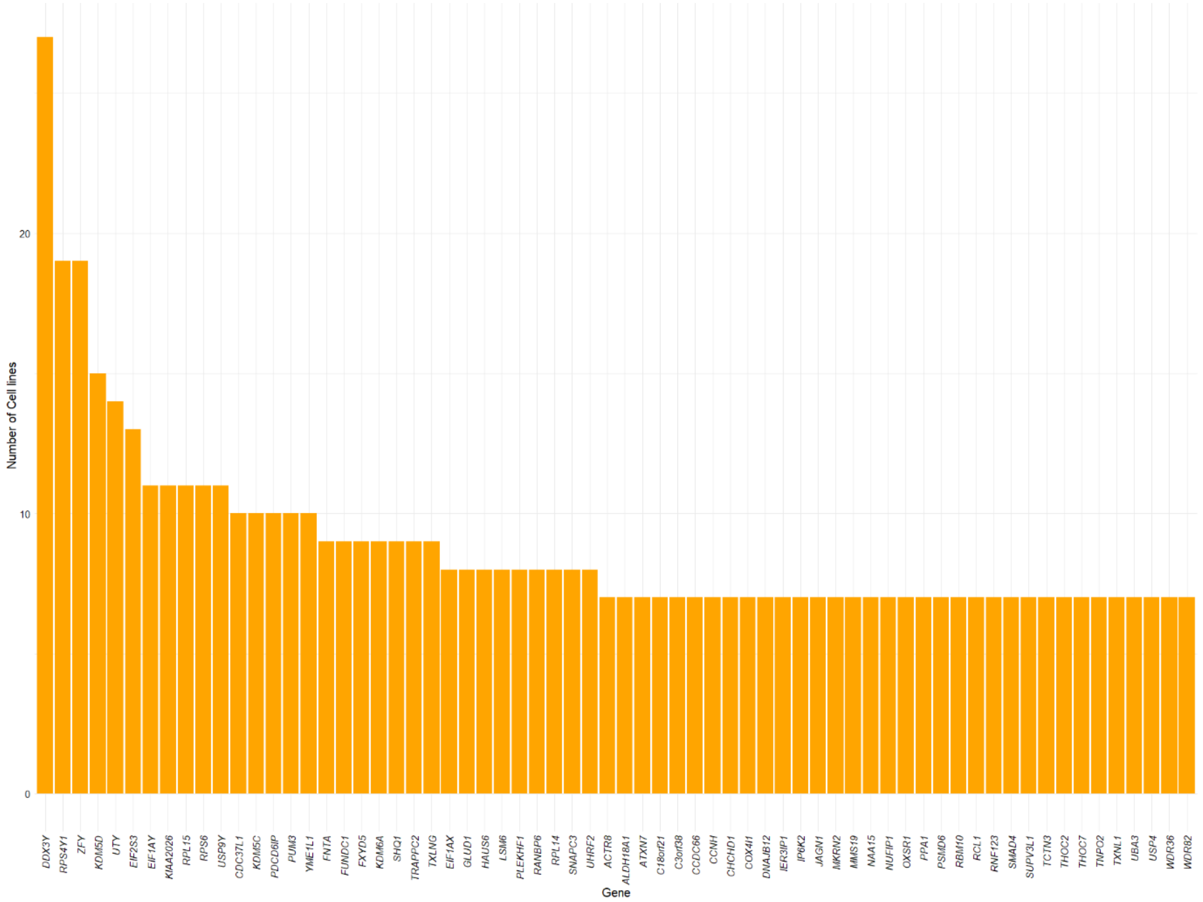

Supplementary Fig. 3. The number of (**a**) overexpressed and (**b**) underexpressed genes in bortezomib-insensitive cancer cell lines.

Supplementary Fig. 4

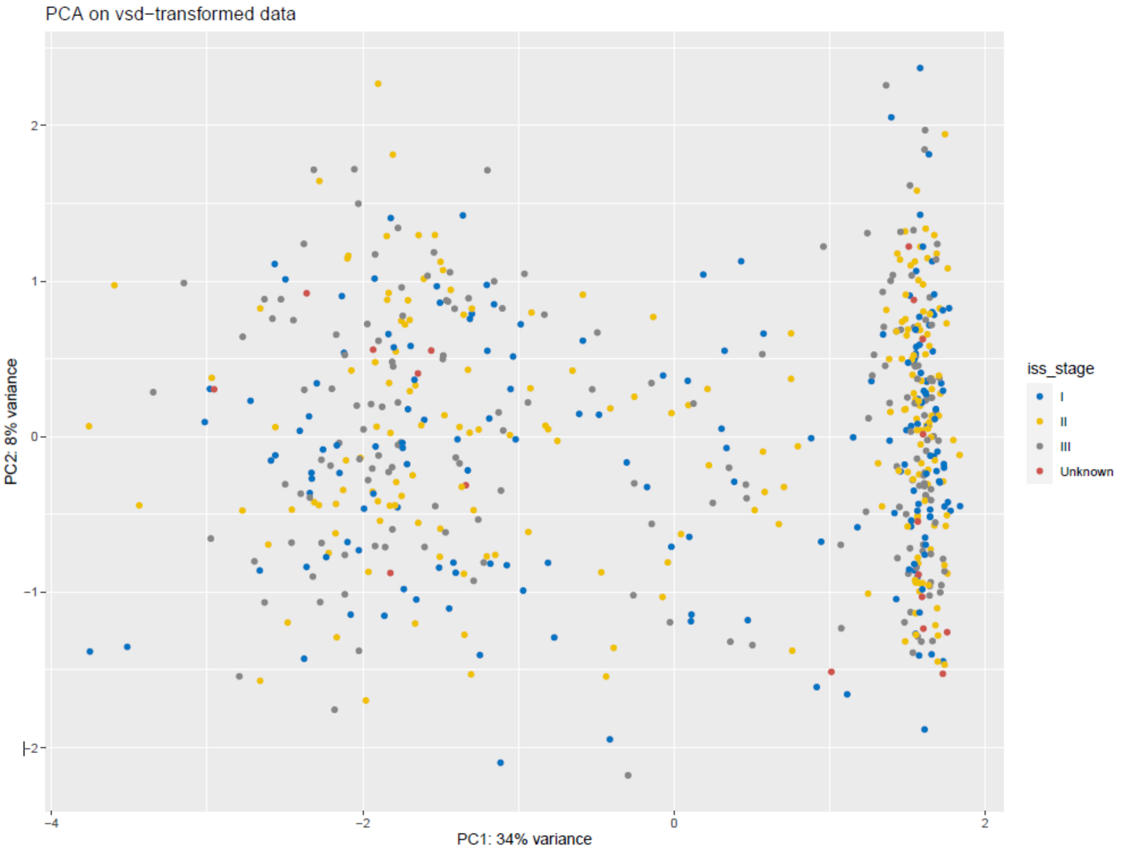

Supplementary Fig. 4. Principal component analysis using the gene expression patterns of 220 genes associated with bortezomib sensitivity stratified 589 patients (treated with bortezomib; classified by the International Staging System [ISS stage]) from the MMRF-COMMPASS into two clusters.

Supplementary Fig. 5

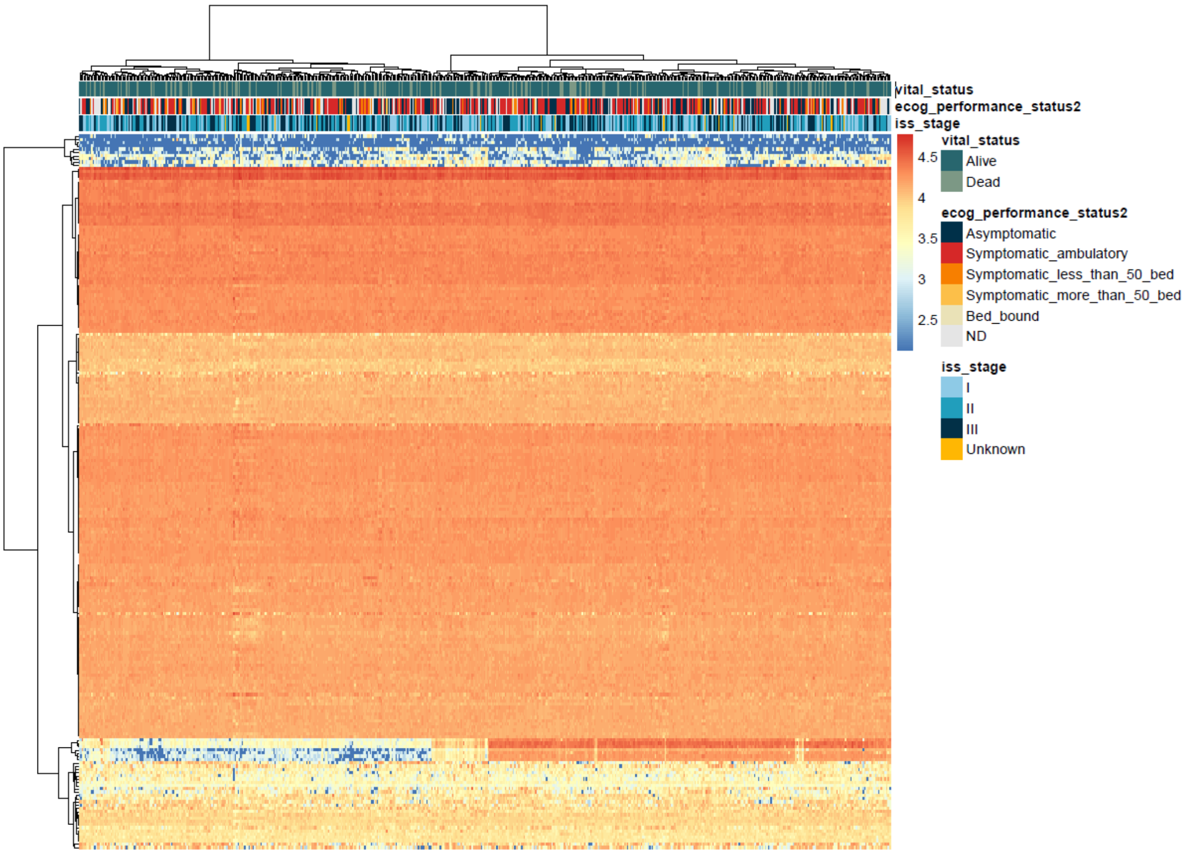

Supplementary Fig. 5. Hierarchical clustering of gene expression patterns for 220/234 genes associated with response to bortezomib treatment and their association with vital status, ECOG performance status, and cancer stage in 589 multiple myeloma patients. Aberrant expression for seven genes was found for the two patient clusters.

Supplementary Fig. 6

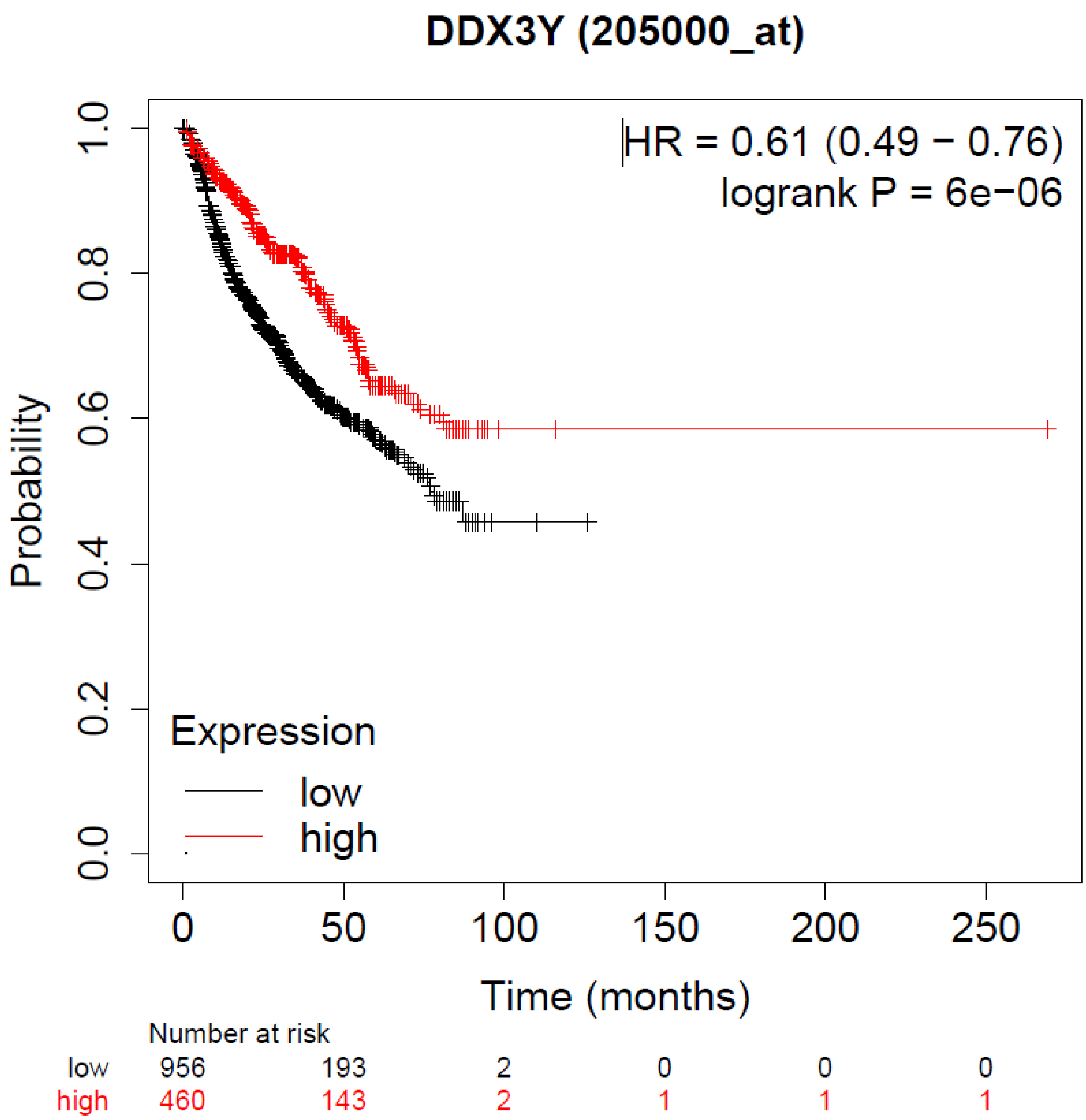

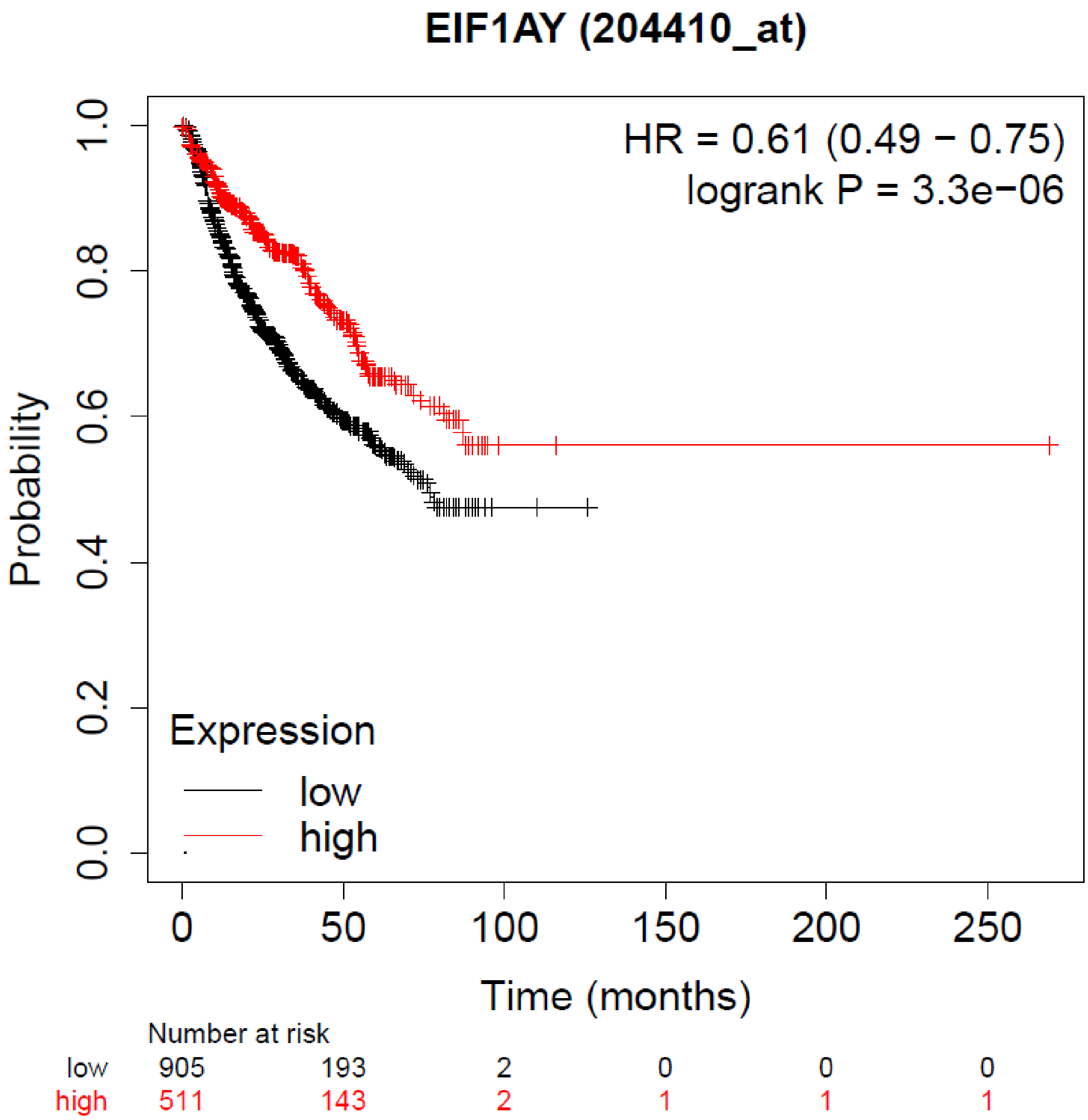
EIF1AY (204410_at)
HR = 0.61 (0.49 − 0.75)
logrank P = 3.3e−06
Probability
0.0
0.2
0.4
0.6
0.8
1.0
Expression
low
high
0
50
100
150
200
250
Time (months)
Number at risk
low 905 193 2 0 0 0
high 511 143 2 1 1 1

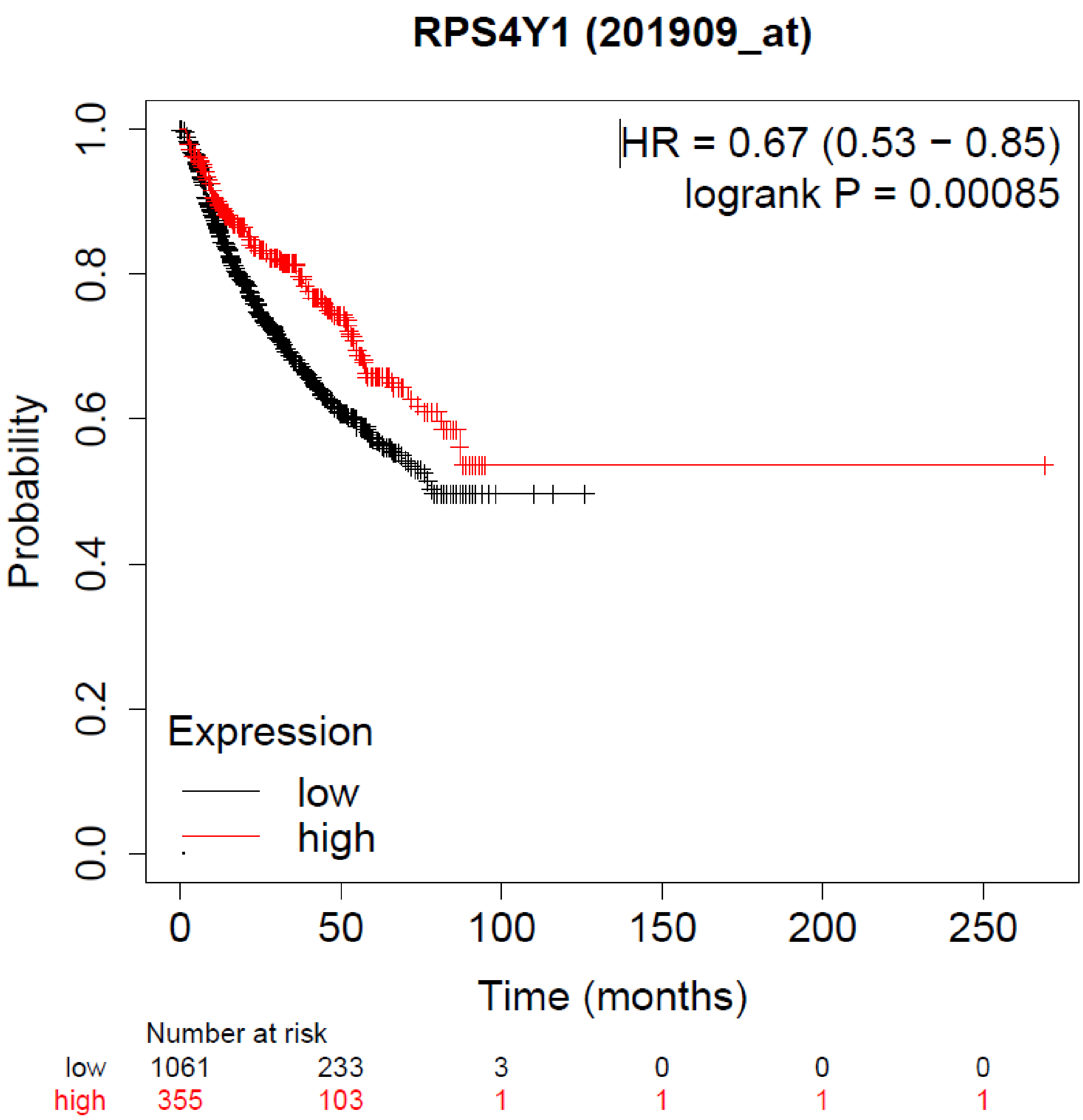
RPS4Y1 (201909_at)
HR = 0.67 (0.53 − 0.85)
logrank P = 0.00085
Probability
0.0
0.2
0.4
0.6
0.8
1.0
Expression
low
high
0
50
100
150
200
250
Time (months)
Number at risk
low 1061 233 3 0 0 0
high 355 103 1 1 1 1

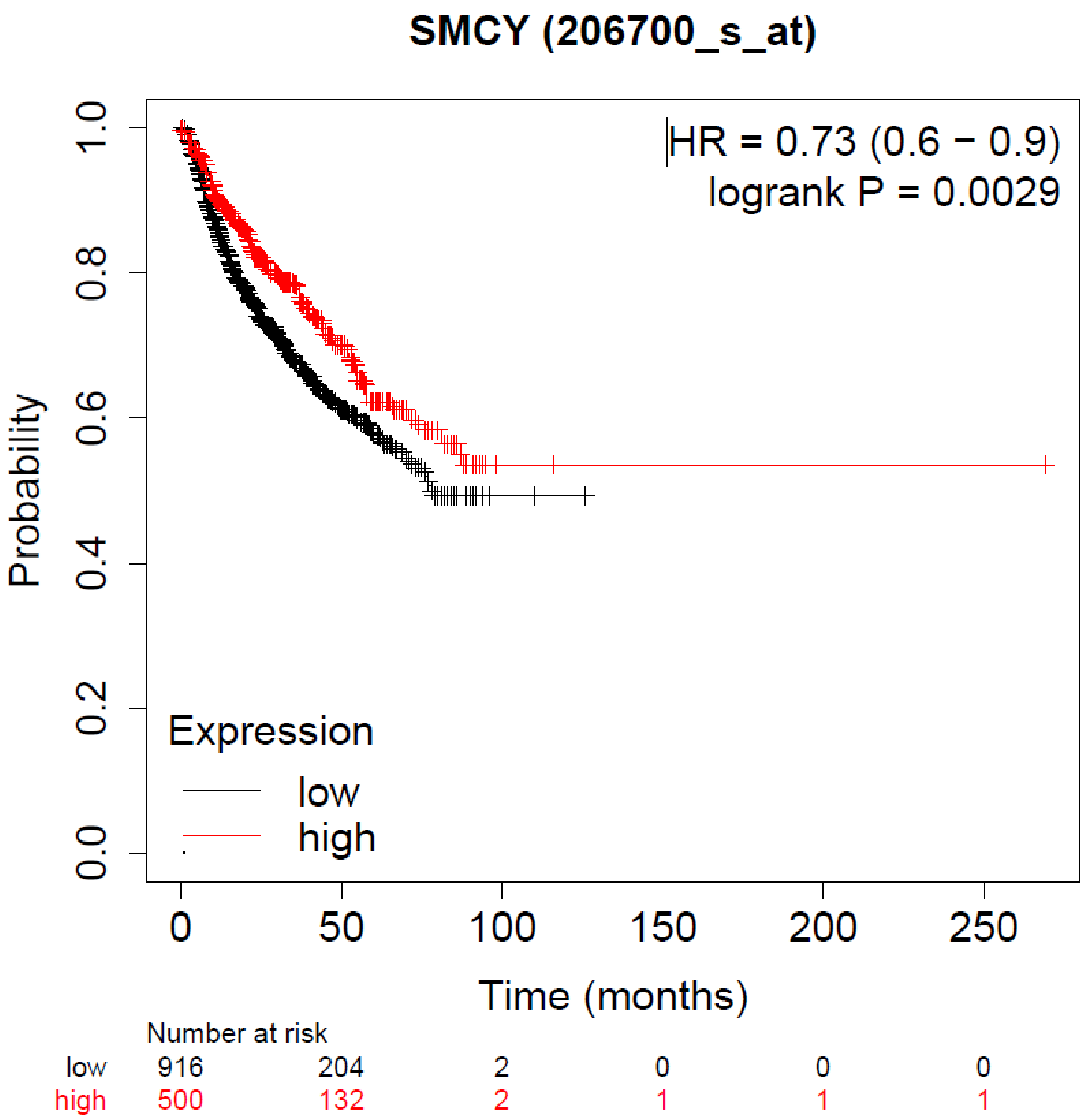
SMCY (206700_s_at)
HR = 0.73 (0.6 − 0.9)
logrank P = 0.0029
Probability
0.0
0.2
0.4
0.6
0.8
1.0
Expression
low
high
0
50
100
150
200
250
Time (months)
Number at risk
low 916 204 2 0 0 0
high 500 132 2 1 1 1

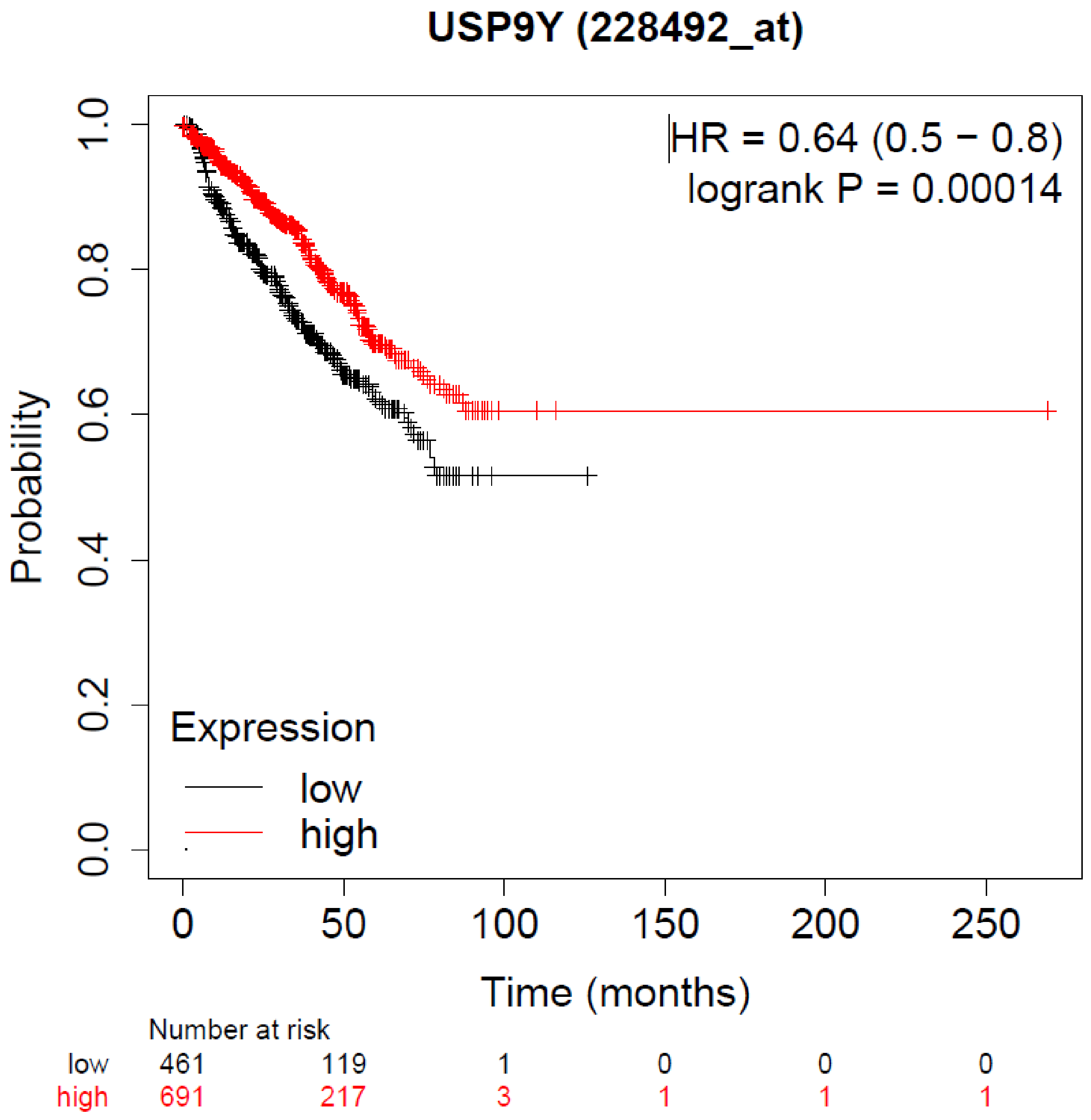
USP9Y (228492_at)
HR = 0.64 (0.5 − 0.8)
logrank P = 0.00014
Probability
0.0
0.2
0.4
0.6
0.8
1.0
Expression
low
high
0
50
100
150
200
250
Time (months)
Number at risk
low 461 119 1 0 0 0
high 691 217 3 1 1 1

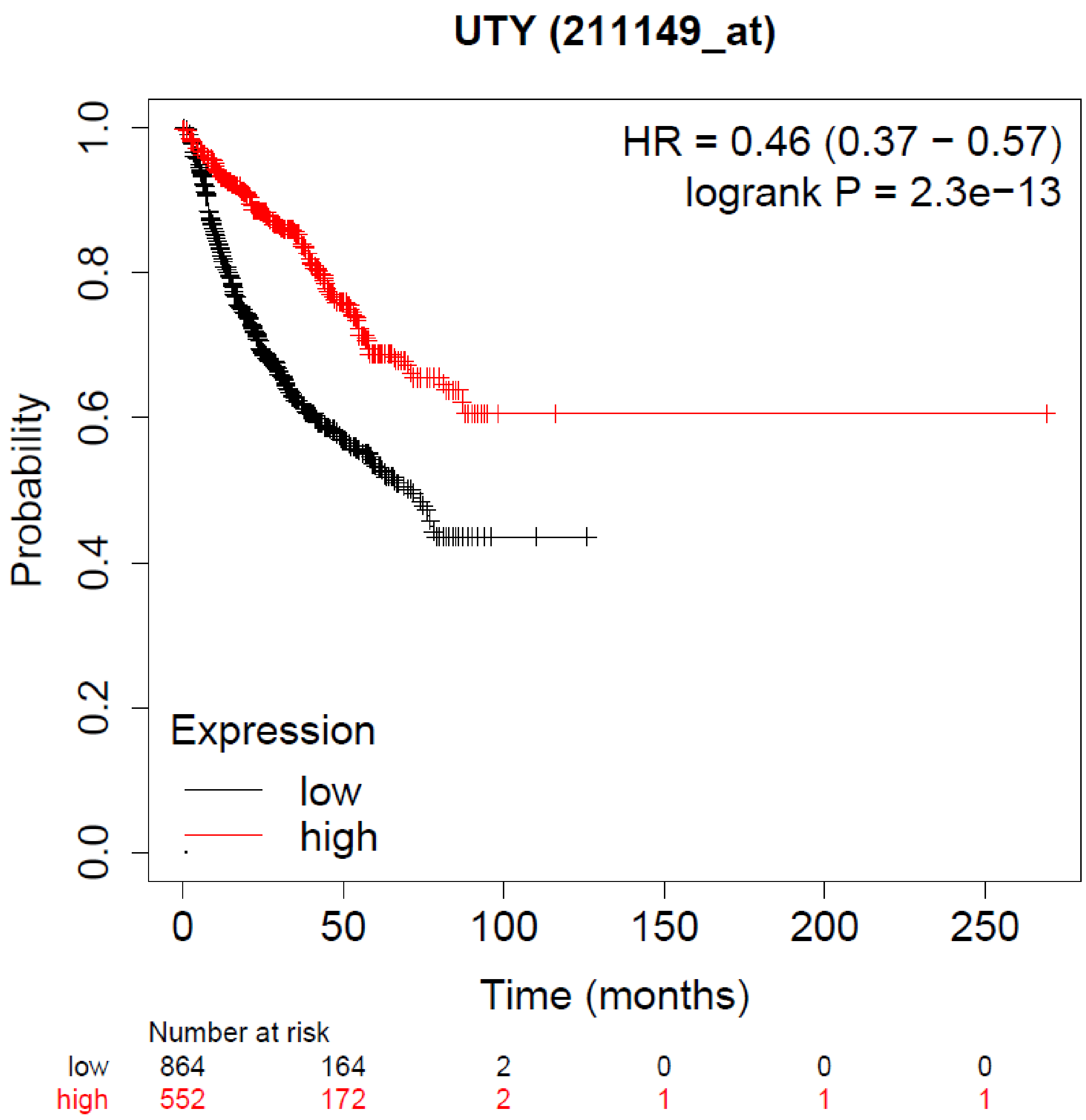
UTY (211149_at)
HR = 0.46 (0.37 − 0.57)
logrank P = 2.3e−13
Probability
0.0
0.2
0.4
0.6
0.8
1.0
Expression
low
high
0
50
100
150
200
250
Time (months)
Number at risk
low 864 164 2 0 0 0
high 552 172 2 1 1 1

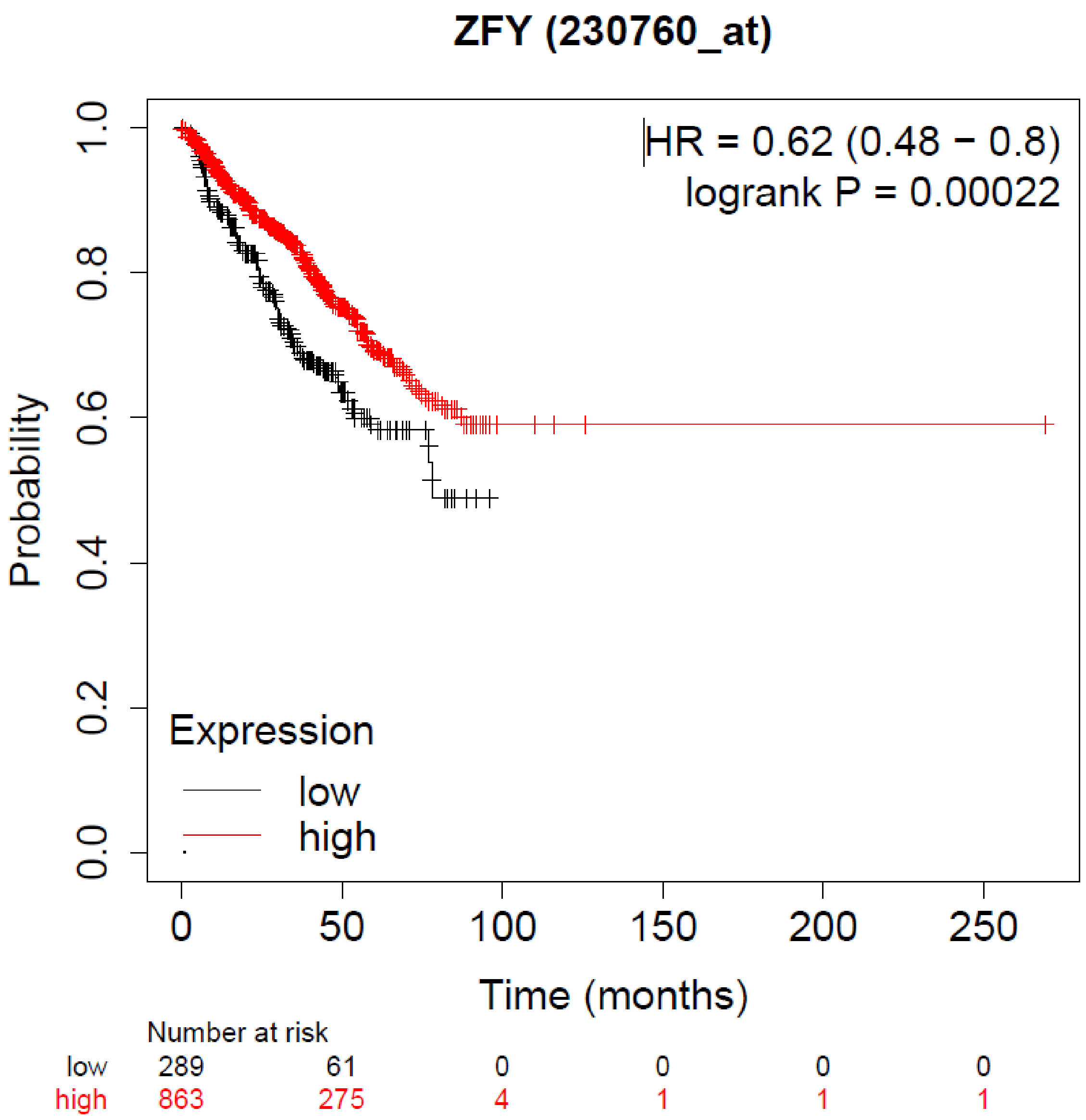

Supplementary Fig. 6. Analysis of overall survival with KM plotter depicting the clinical significance of 7 of 220 (*DDX3Y*, *EIF1AY*, *RPS4Y1*, *SMCY*, *USP9Y*, *UTY*, and *ZFY*; missing data for 14 genes) genes associated with immune response. Low expression of the seven genes was associated with significantly decreased overall survival in multiple myeloma patients.

Supplementary Fig. 7

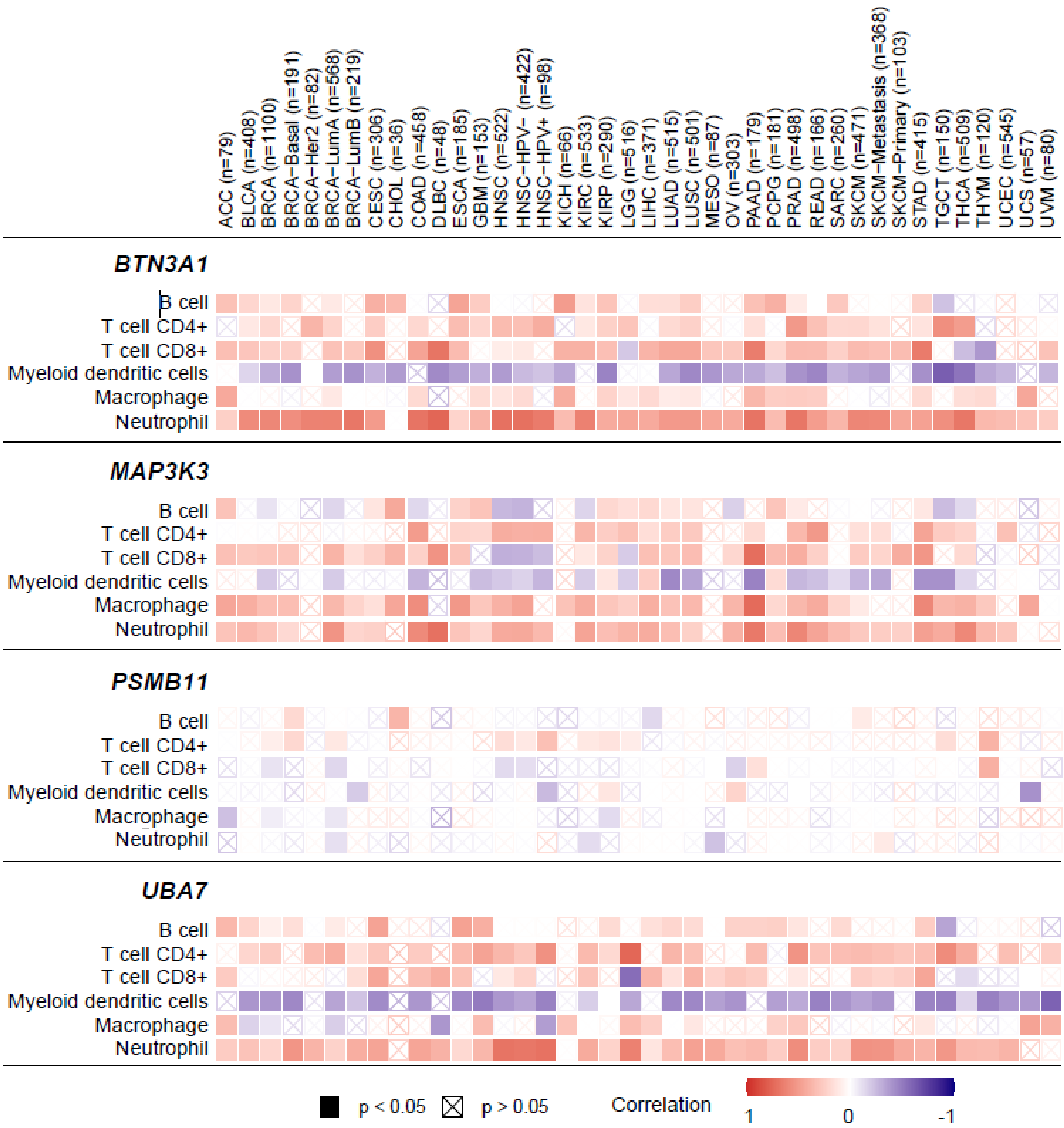

Supplementary Fig. 7. Analyses of gene expression relationship between genes involved in bortezomib-sensitivity and immune cells in different cancer types using TIMER2.0. Genes *BTN3A1*, *MAP3K3*, and *UBA7* had positive expression relationships with the majority of immune infiltrates (B cells, CD4+ T cells, CD8+ T cells, macrophages, and neutrophils) in most cancer types.

Supplementary Fig. 8

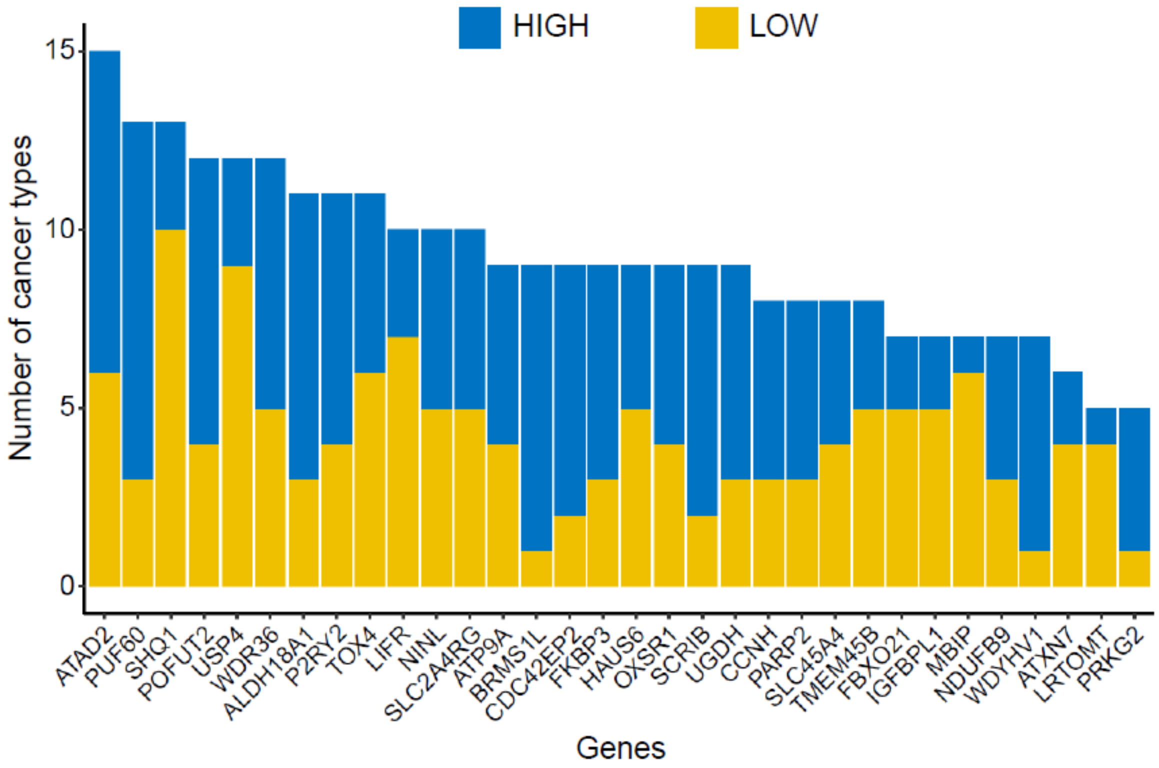

Supplementary Fig. 8. Bar chart depicting the clinical significance of 32 of 33 dysregulated genes on overall survival in 21 different cancer types (pan-cancer study; KM-plotter)

Supplementary Fig. 9

a

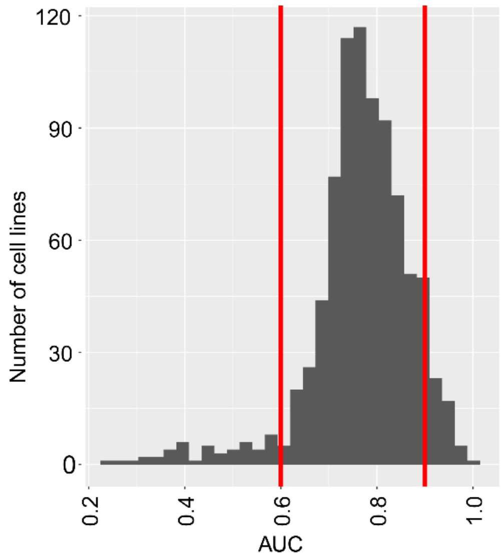

b

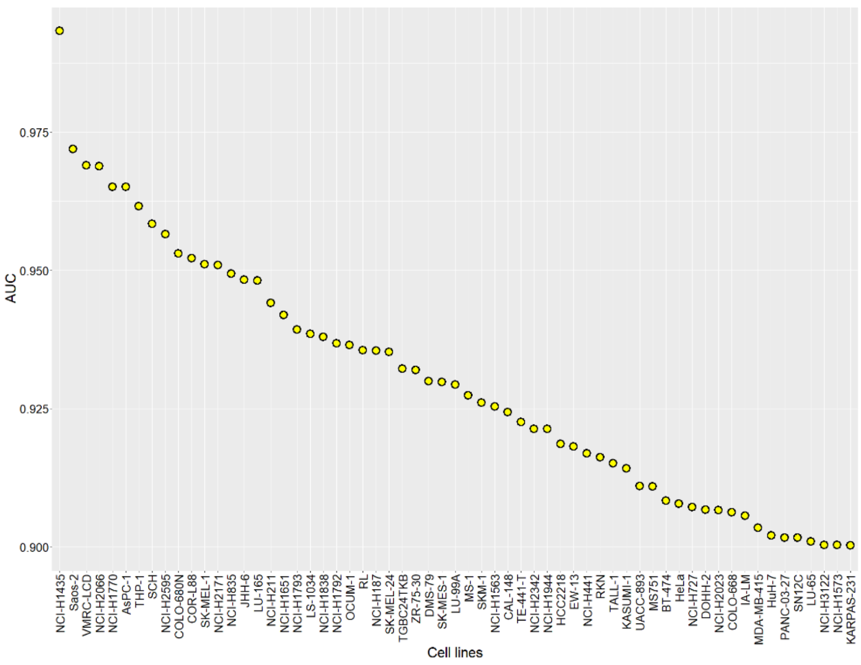

c

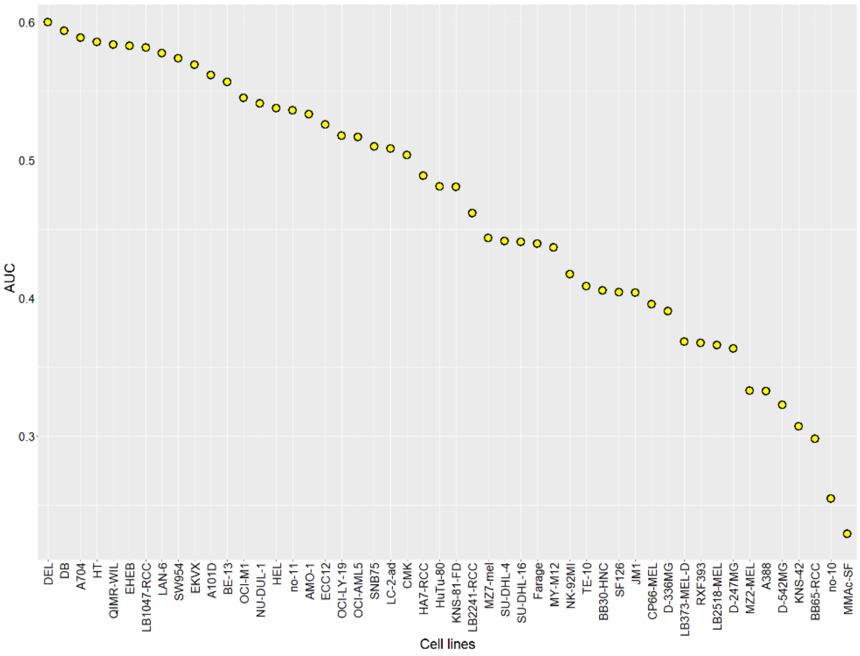
AUC
0.6
0.5
0.4
0.3
DEL
DB
A704
HT
QIMR-WIL
EHEB
LB1047-RCC
LAN-6
SW954
EKVX
A101D
BE-13
OCI-M1
NU-DUL-1
HEL
no-11
AMO-1
ECC12
OCI-LY-19
OCI-AML5
SNB75
LC-2-ad
CMK
HA7-RCC
HuTu-80
KNS-81-FD
LB2241-RCC
MZ7-mel
SU-DHL-4
SU-DHL-16
Farage
MY-M12
NK-92MI
TE-10
BB30-HNC
SF126
JM1
CP66-MEL
D-336MG
LB373-MEL-D
RXF393
LB2518-MEL
D-247MG
MZ2-MEL
A388
D-542MG
KNS-42
BB65-RCC
no-10
MMAc-SF
Cell lines

d

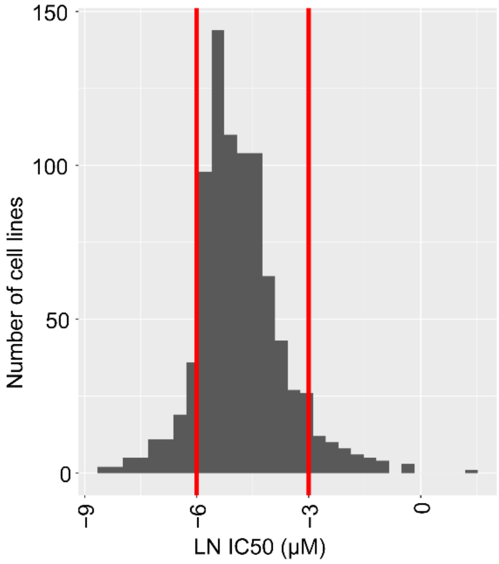
Number of cell lines
150
100
50
0
−9
−6
−3
0
LN IC50 (μM)

e

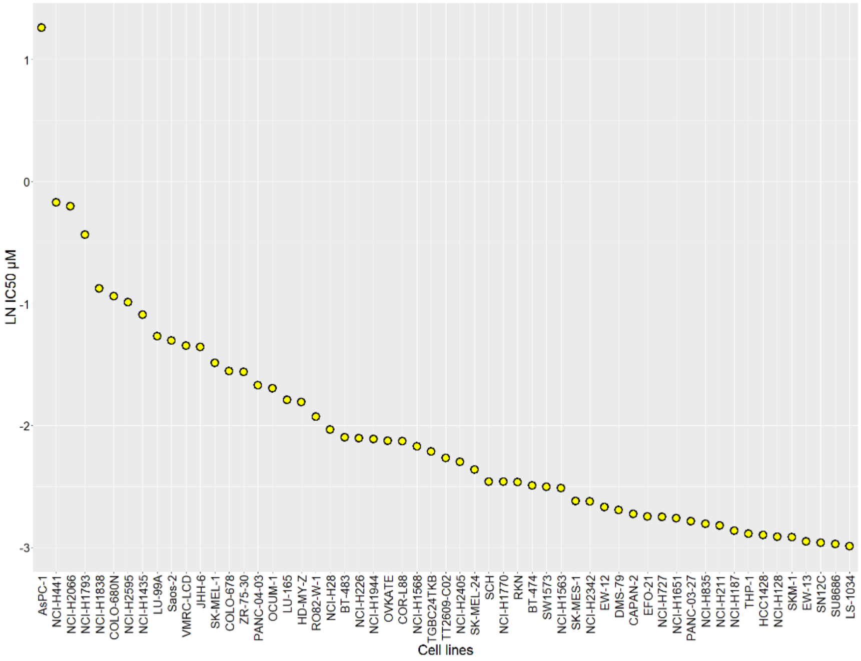

f

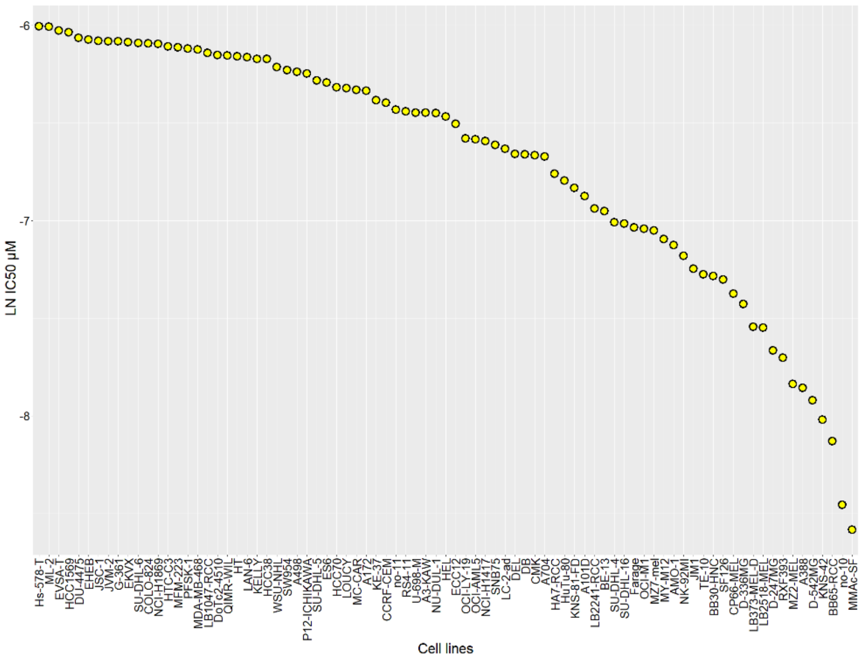

Supplementary Fig. 9. Stratification of cancer cell lines according to bortezomib sensitivity using (**a-c**) area under the curve (AUC) and (**d-f**) natural logarithmic IC50 (LNIC50) values.
