# Supplementary material for: Multi-omics analysis identifies repurposing bortezomib in the treatment of kidney-, nervous system-, and hematological cancers
Source: Sci Rep. 2024 Aug 10;14:18576. doi: 10.1038/s41598-024-62339-x (PMC11316778; doi:10.1038/s41598-024-62339-x)
Supplement: Supplementary file 2 — Supplementary Table 1. [file 41598_2024_62339_MOESM2_ESM.pdf]

**Supplementary Table 1. Merged GDSC1 and GDSC2 datasets (860 cell lines; duplicates were excluded from GDSC2 datasets)**

| Sample name | TCGA | TCGA organ system | Organ system | Organ system subtype | IC50 | AUC | Dataset | LN_IC50 |
|---|---|---|---|---|---|---|---|---|
| SW13 | ACC | Adrenocortical carcinoma | kidney | adrenal gland | 0.004396 | 0.750009 | GDSC2 | -5.42706024244076 |
| BE-13 | ALL | | blood | lymphoblastic leukemia | 0.000957 | 0.55649 | GDSC2 | -6.95170716651132 |
| RS4-11 | ALL | | blood | leukemia | 0.001596 | 0.628903 | GDSC2 | -6.44025477995452 |
| CCRF-CEM | ALL | | blood | lymphoblastic leukemia | 0.001668 | 0.638471 | GDSC2 | -6.39612997504558 |
| KE-37 | ALL | | blood | lymphoblastic leukemia | 0.001688 | 0.63916 | GDSC2 | -6.38421088280837 |
| LOUCY | ALL | | blood | lymphoblastic T cell leukaemia | 0.001798 | 0.645244 | GDSC2 | -6.32108034293271 |
| P12-ICHIKAWA | ALL | | blood | lymphoblastic T cell leukaemia | 0.001936 | 0.653963 | GDSC2 | -6.24713129012775 |
| MOLT-16 | ALL | | blood | lymphoblastic T cell leukaemia | 0.002582 | 0.702028 | GDSC2 | -5.95919098655769 |
| PF-382 | ALL | | blood | lymphoblastic T cell leukaemia | 0.002722 | 0.691608 | GDSC2 | -5.90638837475286 |
| ATN-1 | ALL | | blood | lymphoblastic T cell leukaemia | 0.002872 | 0.712176 | GDSC2 | -5.85274662779616 |
| SUP-B15 | ALL | | blood | lymphoblastic leukemia | 0.002884 | 0.707568 | GDSC2 | -5.84857705955943 |
| DND-41 | ALL | | blood | T cell leukemia | 0.003061 | 0.720152 | GDSC2 | -5.78901361901886 |
| MOLT-13 | ALL | | blood | lymphoblastic T cell leukaemia | 0.003685 | 0.738674 | GDSC2 | -5.60348475334084 |
| LC4-1 | ALL | | blood | lymphoblastic leukemia | 0.003698 | 0.738751 | GDSC2 | -5.5999631460172 |
| ALL-SIL | ALL | | blood | T cell leukemia | 0.003698 | 0.738561 | GDSC2 | -5.5999631460172 |
| RPMI-8402 | ALL | | blood | lymphoblastic T cell leukaemia | 0.003783 | 0.744732 | GDSC2 | -5.57723793333124 |
| MOLT-4 | ALL | | blood | lymphoblastic leukemia | 0.003917 | 0.748356 | GDSC2 | -5.54242922429844 |
| YT | ALL | | blood | lymphoid neoplasm other | 0.004155 | 0.759322 | GDSC2 | -5.48344285067473 |
| Jurkat | ALL | | blood | T cell leukemia | 0.004317 | 0.761854 | GDSC2 | -5.4451945624088 |
| KARPAS-45 | ALL | | blood | lymphoblastic leukemia | 0.004924 | 0.780024 | GDSC2 | -5.31363407065993 |
| GR-ST | ALL | | blood | lymphoblastic leukemia | 0.005176 | 0.742107 | GDSC2 | -5.26372272178354 |
| ALL-PO | ALL | | blood | lymphoblastic leukemia | 0.006435 | 0.804863 | GDSC2 | -5.04600343793405 |
| HAL-01 | ALL | | blood | lymphoblastic leukemia | 0.006949 | 0.81998 | GDSC2 | -4.96915751464995 |
| P30-OHK | ALL | | blood | lymphoblastic leukemia | 0.009578 | 0.856569 | GDSC2 | -4.64828647706172 |
| SUP-T1 | ALL | | blood | lymphoblastic T cell leukaemia | 0.016878 | 0.864406 | GDSC2 | -4.08174428024636 |
| MY-M12 | ALL | | blood | leukemia | 0.000829 | 0.436414 | GDSC1 | -7.09529040282898 |
| TALL-1 | ALL | | blood | lymphoblastic T cell leukaemia | 0.028962 | 0.915064 | GDSC1 | -3.54177065307573 |
| DSH1 | BLCA | Bladder Urothelial Carcinoma | urogenital_system | bladder | 0.002499 | 0.665312 | GDSC2 | -5.99186462712932 |
| BFTC-905 | BLCA | Bladder Urothelial Carcinoma | urogenital_system | bladder | 0.003081 | 0.707875 | GDSC2 | -5.78250105936761 |
| SW1710 | BLCA | Bladder Urothelial Carcinoma | urogenital_system | bladder | 0.003842 | 0.714053 | GDSC2 | -5.56176221463577 |
| RT-112 | BLCA | Bladder Urothelial Carcinoma | urogenital_system | bladder | 0.00408 | 0.727563 | GDSC2 | -5.50165829056607 |
| 647-V | BLCA | Bladder Urothelial Carcinoma | urogenital_system | bladder | 0.004234 | 0.731285 | GDSC2 | -5.46460810626946 |
| 639-V | BLCA | Bladder Urothelial Carcinoma | urogenital_system | bladder | 0.005423 | 0.766111 | GDSC2 | -5.21710611112321 |
| LB831-BLC | BLCA | Bladder Urothelial Carcinoma | urogenital_system | bladder | 0.005698 | 0.772185 | GDSC2 | -5.16764004290642 |
| CAL-29 | BLCA | Bladder Urothelial Carcinoma | urogenital_system | bladder | 0.006126 | 0.762964 | GDSC2 | -5.09521327057155 |
| 5637 | BLCA | Bladder Urothelial Carcinoma | urogenital_system | bladder | 0.00647 | 0.756405 | GDSC2 | -5.04057917046933 |
| VM-CUB-1 | BLCA | Bladder Urothelial Carcinoma | urogenital_system | bladder | 0.006789 | 0.784535 | GDSC2 | -4.99245162366263 |
| KU-19-19 | BLCA | Bladder Urothelial Carcinoma | urogenital_system | bladder | 0.007974 | 0.815 | GDSC2 | -4.83156903002297 |
| HT-1197 | BLCA | Bladder Urothelial Carcinoma | urogenital_system | bladder | 0.011334 | 0.79668 | GDSC2 | -4.47994822123471 |
| RT4 | BLCA | Bladder Urothelial Carcinoma | urogenital_system | bladder | 0.013787 | 0.852583 | GDSC2 | -4.28402915979265 |
| TCCSUP | BLCA | Bladder Urothelial Carcinoma | urogenital_system | bladder | 0.014242 | 0.813175 | GDSC2 | -4.2515599334222 |
| UM-UC-3 | BLCA | Bladder Urothelial Carcinoma | urogenital_system | bladder | 0.014611 | 0.859588 | GDSC2 | -4.22598060929219 |
| SW780 | BLCA | Bladder Urothelial Carcinoma | urogenital_system | bladder | 0.016744 | 0.83823 | GDSC2 | -4.08971529383844 |
| J82 | BLCA | Bladder Urothelial Carcinoma | urogenital_system | bladder | 0.017485 | 0.817975 | GDSC2 | -4.0464119084668 |
| HT-1376 | BLCA | Bladder Urothelial Carcinoma | urogenital_system | bladder | 0.018407 | 0.817148 | GDSC2 | -3.99502425193155 |
| HCC70 | BRCA | Breast invasive carcinoma | breast | breast | 0.001804 | 0.642577 | GDSC2 | -6.31774885734171 |
| HCC38 | BRCA | Breast invasive carcinoma | breast | breast | 0.002086 | 0.639714 | GDSC2 | -6.17250692240356 |
| MDA-MB-468 | BRCA | Breast invasive carcinoma | breast | breast | 0.002189 | 0.662593 | GDSC2 | -6.12431046044141 |
| MFM-223 | BRCA | Breast invasive carcinoma | breast | breast | 0.002215 | 0.629481 | GDSC2 | -6.11250287548504 |
| COLO-824 | BRCA | Breast invasive carcinoma | breast | breast | 0.002257 | 0.655274 | GDSC2 | -6.09371878114674 |
| DU-4475 | BRCA | Breast invasive carcinoma | breast | breast | 0.002324 | 0.684732 | GDSC2 | -6.06446543999247 |
| HCC1569 | BRCA | Breast invasive carcinoma | breast | breast | 0.002391 | 0.672283 | GDSC2 | -6.03604359050595 |
| EVSA-T | BRCA | Breast invasive carcinoma | breast | breast | 0.002414 | 0.684355 | GDSC2 | -6.0264701563068 |
| Hs-578-T | BRCA | Breast invasive carcinoma | breast | breast | 0.002468 | 0.667063 | GDSC2 | -6.004347172939 |
| HCC1500 | BRCA | Breast invasive carcinoma | breast | breast | 0.002649 | 0.6852 | GDSC2 | -5.9335730686922 |
| HCC1187 | BRCA | Breast invasive carcinoma | breast | breast | 0.002852 | 0.693053 | GDSC2 | -5.85973477643009 |
| MDA-MB-231 | BRCA | Breast invasive carcinoma | breast | breast | 0.003171 | 0.706124 | GDSC2 | -5.75370828342593 |
| MDA-MB-436 | BRCA | Breast invasive carcinoma | breast | breast | 0.003211 | 0.688414 | GDSC2 | -5.74117286387476 |
| OCUB-M | BRCA | Breast invasive carcinoma | breast | breast | 0.00325 | 0.720322 | GDSC2 | -5.72910028264049 |
| EFM-192A | BRCA | Breast invasive carcinoma | breast | breast | 0.003256 | 0.694075 | GDSC2 | -5.72725583084184 |
| MRK-nu-1 | BRCA | Breast invasive carcinoma | breast | breast | 0.003295 | 0.729517 | GDSC2 | -5.71534911102767 |
| HCC1937 | BRCA | Breast invasive carcinoma | breast | breast | 0.003336 | 0.692286 | GDSC2 | -5.70298279448564 |
| CAL-85-1 | BRCA | Breast invasive carcinoma | breast | breast | 0.003492 | 0.706463 | GDSC2 | -5.65728064100478 |
| HCC2157 | BRCA | Breast invasive carcinoma | breast | breast | 0.003592 | 0.709746 | GDSC2 | -5.62904612854218 |
| CAMA-1 | BRCA | Breast invasive carcinoma | breast | breast | 0.003814 | 0.729714 | GDSC2 | -5.56907677180391 |
| HDQ-P1 | BRCA | Breast invasive carcinoma | breast | breast | 0.003873 | 0.712736 | GDSC2 | -5.55372587844952 |
| HCC1954 | BRCA | Breast invasive carcinoma | breast | breast | 0.004136 | 0.737647 | GDSC2 | -5.48802614177601 |
| MDA-MB-157 | BRCA | Breast invasive carcinoma | breast | breast | 0.00425 | 0.729529 | GDSC2 | -5.46083629604581 |
| CAL-120 | BRCA | Breast invasive carcinoma | breast | breast | 0.004428 | 0.670679 | GDSC2 | -5.41980726413575 |
| JIMT-1 | BRCA | Breast invasive carcinoma | breast | breast | 0.004485 | 0.741593 | GDSC2 | -5.40701678347138 |
| AU565 | BRCA | Breast invasive carcinoma | breast | breast | 0.004536 | 0.741267 | GDSC2 | -5.39570971255669 |
| EFM-19 | BRCA | Breast invasive carcinoma | breast | breast | 0.004765 | 0.732567 | GDSC2 | -5.34645774187597 |
| T47D | BRCA | Breast invasive carcinoma | breast | breast | 0.005283 | 0.752964 | GDSC2 | -5.24326116079996 |
| CAL-51 | BRCA | Breast invasive carcinoma | breast | breast | 0.005361 | 0.747837 | GDSC2 | -5.22860475413674 |
| MDA-MB-453 | BRCA | Breast invasive carcinoma | breast | breast | 0.006276 | 0.768514 | GDSC2 | -5.07102244411135 |
| BT-549 | BRCA | Breast invasive carcinoma | breast | breast | 0.00664 | 0.751293 | GDSC2 | -5.01464331549379 |
| MCF7 | BRCA | Breast invasive carcinoma | breast | breast | 0.006897 | 0.754161 | GDSC2 | -4.97666874453298 |
| MDA-MB-175-VII | BRCA | Breast invasive carcinoma | breast | breast | 0.007081 | 0.713249 | GDSC2 | -4.9503401383125 |
| HCC1395 | BRCA | Breast invasive carcinoma | breast | breast | 0.007717 | 0.788616 | GDSC2 | -4.86432959150667 |
| HCC1806 | UNCLASSIFIED | Breast invasive carcinoma | breast | breast | 0.007902 | 0.818282 | GDSC2 | -4.84063938699294 |
| HCC1599 | BRCA | Breast invasive carcinoma | breast | breast | 0.00813 | 0.769155 | GDSC2 | -4.81219435542242 |
| UACC-812 | BRCA | Breast invasive carcinoma | breast | breast | 0.010719 | 0.799262 | GDSC2 | -4.53573741127275 |
| BT-20 | BRCA | Breast invasive carcinoma | breast | breast | 0.012457 | 0.866191 | GDSC2 | -4.38547256507818 |
| HCC1419 | BRCA | Breast invasive carcinoma | breast | breast | 0.012967 | 0.858717 | GDSC2 | -4.34534761041539 |
| MDA-MB-361 | BRCA | Breast invasive carcinoma | breast | breast | 0.014411 | 0.862036 | GDSC2 | -4.23976347512591 |
| HCC1143 | BRCA | Breast invasive carcinoma | breast | breast | 0.015483 | 0.825694 | GDSC2 | -4.16801263114744 |
| MDA-MB-415 | BRCA | Breast invasive carcinoma | breast | breast | 0.020433 | 0.903409 | GDSC2 | -3.89060403805485 |
| HCC2218 | BRCA | Breast invasive carcinoma | breast | breast | 0.027849 | 0.918578 | GDSC2 | -3.58095821989623 |
| MDA-MB-330 | BRCA | Breast invasive carcinoma | breast | breast | 0.03548 | 0.885843 | GDSC2 | -3.33878612154076 |
| UACC-893 | BRCA | Breast invasive carcinoma | breast | breast | 0.03895 | 0.91096 | GDSC2 | -3.24547650666538 |
| CAL-148 | BRCA | Breast invasive carcinoma | breast | breast | 0.041707 | 0.924385 | GDSC2 | -3.17708629855782 |
| HCC1428 | BRCA | Breast invasive carcinoma | breast | breast | 0.055102 | 0.86536 | GDSC2 | -2.89856926584136 |
| BT-474 | BRCA | Breast invasive carcinoma | breast | breast | 0.08264 | 0.908345 | GDSC2 | -2.49326145417075 |
| BT-483 | BRCA | Breast invasive carcinoma | breast | breast | 0.122758 | 0.877155 | GDSC2 | -2.09754034131509 |
| DoTc2-4510 | CESC | Cervical squamous cell carcinoma | urogenital_system | cervix | 0.002128 | 0.644819 | GDSC2 | -6.15257270750274 |
| Ca-Ski | CESC | Cervical squamous cell carcinoma | urogenital_system | cervix | 0.00335 | 0.698085 | GDSC2 | -5.69879493314516 |
| C-4-I | CESC | Cervical squamous cell carcinoma | urogenital_system | cervix | 0.004911 | 0.750522 | GDSC2 | -5.31627769192503 |
| SISO | CESC | Cervical squamous cell carcinoma | urogenital_system | cervix | 0.006234 | 0.760178 | GDSC2 | -5.07773709763699 |
| CAL-39 | CESC | Cervical squamous cell carcinoma | urogenital_system | cervix | 0.007576 | 0.807078 | GDSC2 | -4.88276992309836 |
| C-33-A | CESC | Cervical squamous cell carcinoma | urogenital_system | cervix | 0.010208 | 0.81026 | GDSC2 | -4.5845835523797 |

**Supplementary Table 1. Merged GDSC1 and GDSC2 datasets (860 cell lines; duplicates were excluded from GDSC2 datasets)**

| Sample name | TCGA | TCGA organ system | Organ system | Organ system subtype | IC50 | AUC | Dataset | LN_IC50 |
|---|---|---|---|---|---|---|---|---|
| ME-180 | CESC | Cervical squamous cell carcinoma | urogenital_system | cervix | 0.016729 | 0.848356 | GDSC2 | -4.09061153863299 |
| SiHa | CESC | Cervical squamous cell carcinoma | urogenital_system | cervix | 0.017849 | 0.842284 | GDSC2 | -4.02580779473477 |
| HeLa | CESC | Cervical squamous cell carcinoma | urogenital_system | cervix | 0.019769 | 0.907756 | GDSC2 | -3.92364022476834 |
| SKG-IIIa | CESC | Cervical squamous cell carcinoma | urogenital_system | cervix | 0.023368 | 0.855899 | GDSC2 | -3.7563877138967 |
| MS751 | CESC | Cervical squamous cell carcinoma | urogenital_system | cervix | 0.026097 | 0.910864 | GDSC2 | -3.64593491378862 |
| SW756 | CESC | Cervical squamous cell carcinoma | urogenital_system | cervix | 0.027852 | 0.830949 | GDSC2 | -3.58085050190184 |
| HT-3 | CESC | Cervical squamous cell carcinoma | urogenital_system | cervix | 0.042169 | 0.898975 | GDSC2 | -3.16606992504527 |
| OMC-1 | CESC | Cervical squamous cell carcinoma | urogenital_system | cervix | 0.004445 | 0.691583 | GDSC1 | -5.41597541001627 |
| JVM-2 | CLL | | blood | lymphoid neoplasm other | 0.002283 | 0.65632 | GDSC2 | -6.08226491143448 |
| JVM-3 | CLL | | blood | lymphoid neoplasm other | 0.003114 | 0.709535 | GDSC2 | -5.77184720557033 |
| EHEB | CLL | | blood | lymphoid neoplasm other | 0.002302 | 0.582555 | GDSC1 | -6.07397696868245 |
| DiFi | COADREAD | Colon adenocarcinoma and Rectu | digestive_system | large intestine | 0.003366 | 0.695419 | GDSC2 | -5.69403018321352 |
| CCK-81 | COADREAD | Colon adenocarcinoma and Rectu | digestive_system | large intestine | 0.003477 | 0.714156 | GDSC2 | -5.66158542595641 |
| KM12 | COADREAD | Colon adenocarcinoma and Rectu | digestive_system | large intestine | 0.003637 | 0.71078 | GDSC2 | -5.61659611297728 |
| SK-CO-1 | COADREAD | Colon adenocarcinoma and Rectu | digestive_system | large intestine | 0.004204 | 0.730712 | GDSC2 | -5.47171882596743 |
| MDST8 | COADREAD | Colon adenocarcinoma and Rectu | digestive_system | large intestine | 0.004257 | 0.731836 | GDSC2 | -5.45919059213612 |
| RKO | COADREAD | Colon adenocarcinoma and Rectu | digestive_system | large intestine | 0.004349 | 0.741157 | GDSC2 | -5.43780934536664 |
| SNU-407 | COADREAD | Colon adenocarcinoma and Rectu | digestive_system | large intestine | 0.005004 | 0.742888 | GDSC2 | -5.29751768637747 |
| CL-40 | COADREAD | Colon adenocarcinoma and Rectu | digestive_system | large intestine | 0.005357 | 0.7446 | GDSC2 | -5.22935116208331 |
| CaR-1 | COADREAD | Colon adenocarcinoma and Rectu | digestive_system | large intestine | 0.005514 | 0.756818 | GDSC2 | -5.20046496638053 |
| COLO-205 | COADREAD | Colon adenocarcinoma and Rectu | digestive_system | large intestine | 0.006228 | 0.75849 | GDSC2 | -5.07870002501039 |
| COLO-320-HSR | COADREAD | Colon adenocarcinoma and Rectu | digestive_system | large intestine | 0.006373 | 0.795976 | GDSC2 | -5.05568496264998 |
| SNU-C5 | COADREAD | Colon adenocarcinoma and Rectu | digestive_system | large intestine | 0.006413 | 0.755271 | GDSC2 | -5.04942809881541 |
| CL-11 | COADREAD | Colon adenocarcinoma and Rectu | digestive_system | large intestine | 0.006733 | 0.713201 | GDSC2 | -5.00073446941899 |
| HCT-15 | COADREAD | Colon adenocarcinoma and Rectu | digestive_system | large intestine | 0.006818 | 0.78332 | GDSC2 | -4.98818910526643 |
| SW620 | COADREAD | Colon adenocarcinoma and Rectu | digestive_system | large intestine | 0.007088 | 0.760665 | GDSC2 | -4.94935206567936 |
| HT-29 | COADREAD | Colon adenocarcinoma and Rectu | digestive_system | large intestine | 0.007223 | 0.761498 | GDSC2 | -4.93048489991343 |
| GP5d | COADREAD | Colon adenocarcinoma and Rectu | digestive_system | large intestine | 0.007464 | 0.81439 | GDSC2 | -4.89766381543709 |
| NCI-H716 | COADREAD | Colon adenocarcinoma and Rectu | digestive_system | large intestine | 0.00754 | 0.77186 | GDSC2 | -4.88753309696227 |
| HCC2998 | COADREAD | Colon adenocarcinoma and Rectu | digestive_system | large intestine | 0.007574 | 0.81298 | GDSC2 | -4.88303394950253 |
| SW948 | COADREAD | Colon adenocarcinoma and Rectu | digestive_system | large intestine | 0.007689 | 0.779042 | GDSC2 | -4.86796454293209 |
| C2BBe1 | COADREAD | Colon adenocarcinoma and Rectu | digestive_system | large intestine | 0.008508 | 0.747022 | GDSC2 | -4.76674838164415 |
| NCI-H747 | COADREAD | Colon adenocarcinoma and Rectu | digestive_system | large intestine | 0.008898 | 0.752958 | GDSC2 | -4.72192874659829 |
| SW1463 | COADREAD | Colon adenocarcinoma and Rectu | digestive_system | large intestine | 0.010472 | 0.818843 | GDSC2 | -4.55905025037454 |
| SNU-C2B | COADREAD | Colon adenocarcinoma and Rectu | digestive_system | large intestine | 0.010519 | 0.763772 | GDSC2 | -4.554572133225 |
| SW837 | COADREAD | Colon adenocarcinoma and Rectu | digestive_system | large intestine | 0.011329 | 0.8076 | GDSC2 | -4.48038946909079 |
| HCT-116 | COADREAD | Colon adenocarcinoma and Rectu | digestive_system | large intestine | 0.011789 | 0.863381 | GDSC2 | -4.44058838567215 |
| LoVo | COADREAD | Colon adenocarcinoma and Rectu | digestive_system | large intestine | 0.013459 | 0.819513 | GDSC2 | -4.30810725173056 |
| SNU-175 | COADREAD | Colon adenocarcinoma and Rectu | digestive_system | large intestine | 0.014786 | 0.863801 | GDSC2 | -4.21407449184723 |
| LS-411N | COADREAD | Colon adenocarcinoma and Rectu | digestive_system | large intestine | 0.015031 | 0.838414 | GDSC2 | -4.19764054383105 |
| SW48 | COADREAD | Colon adenocarcinoma and Rectu | digestive_system | large intestine | 0.015167 | 0.828258 | GDSC2 | -4.18863326391288 |
| CW-2 | COADREAD | Colon adenocarcinoma and Rectu | digestive_system | large intestine | 0.015808 | 0.871272 | GDSC2 | -4.14723913797662 |
| HT55 | COADREAD | Colon adenocarcinoma and Rectu | digestive_system | large intestine | 0.016962 | 0.841656 | GDSC2 | -4.07677973104263 |
| T84 | COADREAD | Colon adenocarcinoma and Rectu | digestive_system | large intestine | 0.017686 | 0.858072 | GDSC2 | -4.03498191282859 |
| LS-123 | COADREAD | Colon adenocarcinoma and Rectu | digestive_system | large intestine | 0.017821 | 0.799228 | GDSC2 | -4.02737774179117 |
| LS-180 | COADREAD | Colon adenocarcinoma and Rectu | digestive_system | large intestine | 0.022817 | 0.860428 | GDSC2 | -3.7802494068187 |
| SNU-C1 | COADREAD | Colon adenocarcinoma and Rectu | digestive_system | large intestine | 0.023419 | 0.860003 | GDSC2 | -3.75420762027242 |
| SW1116 | COADREAD | Colon adenocarcinoma and Rectu | digestive_system | large intestine | 0.028422 | 0.873138 | GDSC2 | -3.56059178581247 |
| RCM-1 | COADREAD | Colon adenocarcinoma and Rectu | digestive_system | large intestine | 0.043379 | 0.865418 | GDSC2 | -3.13777982594685 |
| SNU-61 | COADREAD | Colon adenocarcinoma and Rectu | digestive_system | large intestine | 0.04475 | 0.890919 | GDSC2 | -3.10666383426127 |
| SW1417 | COADREAD | Colon adenocarcinoma and Rectu | digestive_system | large intestine | 0.046944 | 0.814163 | GDSC2 | -3.05879987702157 |
| LS-1034 | COADREAD | Colon adenocarcinoma and Rectu | digestive_system | large intestine | 0.050327 | 0.938495 | GDSC2 | -2.98921356656688 |
| COLO-678 | COADREAD | Colon adenocarcinoma and Rectu | digestive_system | large intestine | 0.21117 | 0.891542 | GDSC2 | -1.55509178269399 |
| LS-513 | COADREAD | Colon adenocarcinoma and Rectu | digestive_system | large intestine | 0.003371 | 0.648454 | GDSC1 | -5.69254584273021 |
| DB | DLBC | Lymphoid Neoplasm Diffuse Larg | blood | B cell lymphoma | 0.001278 | 0.593621 | GDSC2 | -6.66245892302679 |
| A3-KAW | DLBC | Lymphoid Neoplasm Diffuse Larg | blood | B cell lymphoma | 0.001585 | 0.629828 | GDSC2 | -6.44717087165289 |
| SU-DHL-5 | DLBC | Lymphoid Neoplasm Diffuse Larg | blood | B cell lymphoma | 0.00187 | 0.656607 | GDSC2 | -6.28181684811564 |
| SU-DHL-6 | DLBC | Lymphoid Neoplasm Diffuse Larg | blood | B cell lymphoma | 0.002262 | 0.683333 | GDSC2 | -6.09150590128821 |
| JSC-1 | DLBC | Lymphoid Neoplasm Diffuse Larg | blood | B cell lymphoma | 0.00229 | 0.671725 | GDSC2 | -6.07920346141599 |
| WSU-DLCL2 | DLBC | Lymphoid Neoplasm Diffuse Larg | blood | B cell lymphoma | 0.002662 | 0.694754 | GDSC2 | -5.92867755900922 |
| GRANTA-519 | DLBC | Lymphoid Neoplasm Diffuse Larg | blood | B cell lymphoma | 0.002757 | 0.676448 | GDSC2 | -5.89361214694048 |
| SCC-3 | DLBC | Lymphoid Neoplasm Diffuse Larg | blood | B cell lymphoma | 0.002886 | 0.711008 | GDSC2 | -5.84788381863046 |
| SU-DHL-10 | DLBC | Lymphoid Neoplasm Diffuse Larg | blood | B cell lymphoma | 0.003159 | 0.727928 | GDSC2 | -5.75749975716219 |
| KARPAS-1106P | DLBC | Lymphoid Neoplasm Diffuse Larg | blood | B cell lymphoma | 0.003329 | 0.723383 | GDSC2 | -5.70508332038925 |
| A4-Fuk | DLBC | Lymphoid Neoplasm Diffuse Larg | blood | B cell lymphoma | 0.003491 | 0.73543 | GDSC2 | -5.65756705085924 |
| VAL | DLBC | Lymphoid Neoplasm Diffuse Larg | blood | B cell lymphoma | 0.003772 | 0.738934 | GDSC2 | -5.58014991421093 |
| BC-1 | DLBC | Lymphoid Neoplasm Diffuse Larg | blood | B cell lymphoma | 0.00385 | 0.744907 | GDSC2 | -5.55968213068244 |
| KARPAS-422 | DLBC | Lymphoid Neoplasm Diffuse Larg | blood | B cell lymphoma | 0.003987 | 0.737113 | GDSC2 | -5.52471621058292 |
| SLVL | DLBC | Lymphoid Neoplasm Diffuse Larg | blood | B cell lymphoma | 0.005285 | 0.780302 | GDSC2 | -5.24288265965994 |
| RC-K8 | DLBC | Lymphoid Neoplasm Diffuse Larg | blood | B cell lymphoma | 0.005363 | 0.758281 | GDSC2 | -5.22823175898135 |
| SU-DHL-8 | DLBC | Lymphoid Neoplasm Diffuse Larg | blood | B cell lymphoma | 0.00561 | 0.812988 | GDSC2 | -5.18320455944753 |
| TUR | DLBC | Lymphoid Neoplasm Diffuse Larg | blood | B cell lymphoma | 0.008256 | 0.851999 | GDSC2 | -4.79681507024293 |
| TK | DLBC | Lymphoid Neoplasm Diffuse Larg | blood | B cell lymphoma | 0.008718 | 0.842292 | GDSC2 | -4.74236542516594 |
| JM1 | DLBC | Lymphoid Neoplasm Diffuse Larg | blood | B cell lymphoma | 0.000713 | 0.40372 | GDSC1 | -7.24602913754998 |
| Farage | DLBC | Lymphoid Neoplasm Diffuse Larg | blood | B cell lymphoma | 0.00088 | 0.439512 | GDSC1 | -7.03558865049202 |
| SU-DHL-16 | DLBC | Lymphoid Neoplasm Diffuse Larg | blood | B cell lymphoma | 0.000897 | 0.440521 | GDSC1 | -7.01645469590548 |
| SU-DHL-4 | DLBC | Lymphoid Neoplasm Diffuse Larg | blood | B cell lymphoma | 0.000903 | 0.441334 | GDSC1 | -7.00978800454729 |
| OCI-LY-19 | DLBC | Lymphoid Neoplasm Diffuse Larg | blood | B cell lymphoma | 0.001389 | 0.517631 | GDSC1 | -6.57917121520993 |
| NU-DUL-1 | DLBC | Lymphoid Neoplasm Diffuse Larg | blood | B cell lymphoma | 0.001581 | 0.540821 | GDSC1 | -6.4496977207548 |
| HT | DLBC | Lymphoid Neoplasm Diffuse Larg | blood | B cell lymphoma | 0.002115 | 0.585304 | GDSC1 | -6.1587004664839 |
| BC-3 | DLBC | Lymphoid Neoplasm Diffuse Larg | blood | B cell lymphoma | 0.002564 | 0.622005 | GDSC1 | -5.96618673992371 |
| MC116 | DLBC | Lymphoid Neoplasm Diffuse Larg | blood | B cell lymphoma | 0.003302 | 0.674775 | GDSC1 | -5.7132269334842 |
| CTB-1 | DLBC | Lymphoid Neoplasm Diffuse Larg | blood | B cell lymphoma | 0.006755 | 0.716379 | GDSC1 | -4.99747230756997 |
| DOHH-2 | DLBC | Lymphoid Neoplasm Diffuse Larg | blood | B cell lymphoma | 0.022236 | 0.906667 | GDSC1 | -3.80604268189091 |
| RL | DLBC | Lymphoid Neoplasm Diffuse Larg | blood | B cell lymphoma | 0.041883 | 0.935571 | GDSC1 | -3.17287526230752 |
| T-T | ESCA | Esophageal carcinoma | aero_digestive_tract | oesophagus | 0.003727 | 0.72944 | GDSC2 | -5.59215165848502 |
| KYSE-150 | ESCA | Esophageal carcinoma | aero_digestive_tract | oesophagus | 0.003754 | 0.718085 | GDSC2 | -5.58493334081782 |
| TE-9 | ESCA | Esophageal carcinoma | aero_digestive_tract | oesophagus | 0.004654 | 0.752314 | GDSC2 | -5.37002821410204 |
| OACM5-1 | ESCA | Esophageal carcinoma | aero_digestive_tract | oesophagus | 0.004917 | 0.724573 | GDSC2 | -5.31505669055233 |
| KYSE-180 | ESCA | Esophageal carcinoma | aero_digestive_tract | oesophagus | 0.005569 | 0.77747 | GDSC2 | -5.19053977437461 |
| KYSE-270 | ESCA | Esophageal carcinoma | aero_digestive_tract | oesophagus | 0.005859 | 0.772975 | GDSC2 | -5.13977633841949 |
| TE-8 | ESCA | Esophageal carcinoma | aero_digestive_tract | oesophagus | 0.006091 | 0.764767 | GDSC2 | -5.10094300712779 |
| KYSE-220 | ESCA | Esophageal carcinoma | aero_digestive_tract | oesophagus | 0.006174 | 0.772271 | GDSC2 | -5.08740835290217 |
| KYSE-450 | ESCA | Esophageal carcinoma | aero_digestive_tract | oesophagus | 0.006299 | 0.755521 | GDSC2 | -5.06736438834235 |
| EC-GI-10 | ESCA | Esophageal carcinoma | aero_digestive_tract | oesophagus | 0.00647 | 0.776378 | GDSC2 | -5.04057917046933 |
| KYAE-1 | ESCA | Esophageal carcinoma | aero_digestive_tract | oesophagus | 0.006672 | 0.78073 | GDSC2 | -5.00983561392569 |
| OE19 | ESCA | Esophageal carcinoma | aero_digestive_tract | oesophagus | 0.006763 | 0.754461 | GDSC2 | -4.99628870038933 |
| KYSE-520 | ESCA | Esophageal carcinoma | aero_digestive_tract | oesophagus | 0.006907 | 0.721213 | GDSC2 | -4.9752198888753 |
| KYSE-140 | ESCA | Esophageal carcinoma | aero_digestive_tract | oesophagus | 0.007192 | 0.789453 | GDSC2 | -4.93478598181282 |
| OE21 | ESCA | Esophageal carcinoma | aero_digestive_tract | oesophagus | 0.007239 | 0.77466 | GDSC2 | -4.92827220367113 |

**Supplementary Table 1. Merged GDSC1 and GDSC2 datasets (860 cell lines; duplicates were excluded from GDSC2 datasets)**

| Sample name | TCGA | TCGA organ system | Organ system | Organ system subtype | IC50 | AUC | Dataset | LN_IC50 |
|---|---|---|---|---|---|---|---|---|
| OACp4C | ESCA | Esophageal carcinoma | aero_digestive_tract | oesophagus | 0.007316 | 0.78978 | GDSC2 | -4.91769154845352 |
| TE-1 | ESCA | Esophageal carcinoma | aero_digestive_tract | oesophagus | 0.007346 | 0.77238 | GDSC2 | -4.91359933158466 |
| TE-4 | ESCA | Esophageal carcinoma | aero_digestive_tract | oesophagus | 0.007902 | 0.814157 | GDSC2 | -4.84063938699294 |
| ESO51 | ESCA | Esophageal carcinoma | aero_digestive_tract | oesophagus | 0.008263 | 0.771137 | GDSC2 | -4.79596756126309 |
| OE33 | ESCA | Esophageal carcinoma | aero_digestive_tract | oesophagus | 0.008456 | 0.783638 | GDSC2 | -4.7728790304142 |
| KYSE-410 | ESCA | Esophageal carcinoma | aero_digestive_tract | oesophagus | 0.009103 | 0.75292 | GDSC2 | -4.69915124945899 |
| KYSE-50 | ESCA | Esophageal carcinoma | aero_digestive_tract | oesophagus | 0.011723 | 0.836784 | GDSC2 | -4.44620255489245 |
| FLO-1 | ESCA | Esophageal carcinoma | aero_digestive_tract | oesophagus | 0.01233 | 0.826816 | GDSC2 | -4.39571996180588 |
| TE-5 | ESCA | Esophageal carcinoma | aero_digestive_tract | oesophagus | 0.013107 | 0.82004 | GDSC2 | -4.33460884034473 |
| KYSE-70 | ESCA | Esophageal carcinoma | aero_digestive_tract | oesophagus | 0.014003 | 0.814126 | GDSC2 | -4.2684836866085 |
| ESO26 | ESCA | Esophageal carcinoma | aero_digestive_tract | oesophagus | 0.019534 | 0.875955 | GDSC2 | -3.93559874195685 |
| SK-GT-4 | ESCA | Esophageal carcinoma | aero_digestive_tract | oesophagus | 0.022845 | 0.793993 | GDSC2 | -3.7790230039669 |
| TE-6 | ESCA | Esophageal carcinoma | aero_digestive_tract | oesophagus | 0.041836 | 0.884507 | GDSC2 | -3.17399806609012 |
| COLO-680N | ESCA | Esophageal carcinoma | aero_digestive_tract | oesophagus | 0.390167 | 0.953019 | GDSC2 | -0.941180426383892 |
| TE-10 | ESCA | Esophageal carcinoma | aero_digestive_tract | oesophagus | 0.000693 | 0.408456 | GDSC1 | -7.27448055877437 |
| TE-15 | ESCA | Esophageal carcinoma | aero_digestive_tract | oesophagus | 0.003495 | 0.649714 | GDSC1 | -5.65642190329636 |
| TE-12 | ESCA | Esophageal carcinoma | aero_digestive_tract | oesophagus | 0.005905 | 0.704586 | GDSC1 | -5.13195582933281 |
| A172 | GBM | Glioblastoma multiforme | nervous_system | glioma | 0.001772 | 0.630072 | GDSC2 | -6.33564642679925 |
| SK-MG-1 | GBM | Glioblastoma multiforme | nervous_system | glioma | 0.003041 | 0.684873 | GDSC2 | -5.79556887028691 |
| SW1088 | GBM | Glioblastoma multiforme | nervous_system | glioma | 0.003067 | 0.682412 | GDSC2 | -5.78705539385002 |
| D-263MG | GBM | Glioblastoma multiforme | nervous_system | glioma | 0.003113 | 0.695161 | GDSC2 | -5.77216838752267 |
| LN-18 | GBM | Glioblastoma multiforme | nervous_system | glioma | 0.003426 | 0.707709 | GDSC2 | -5.67636187908021 |
| 8-MG-BA | GBM | Glioblastoma multiforme | nervous_system | glioma | 0.003697 | 0.729145 | GDSC2 | -5.60023359902764 |
| M059J | GBM | Glioblastoma multiforme | nervous_system | glioma | 0.003967 | 0.716804 | GDSC2 | -5.52974513744994 |
| DK-MG | GBM | Glioblastoma multiforme | nervous_system | glioma | 0.00416 | 0.706672 | GDSC2 | -5.48224020470897 |
| T98G | GBM | Glioblastoma multiforme | nervous_system | glioma | 0.004825 | 0.75981 | GDSC2 | -5.33394454419119 |
| D-392MG | GBM | Glioblastoma multiforme | nervous_system | glioma | 0.004916 | 0.746118 | GDSC2 | -5.31526008727835 |
| U-118-MG | GBM | Glioblastoma multiforme | nervous_system | glioma | 0.005243 | 0.727279 | GDSC2 | -5.25086142539174 |
| LN-229 | GBM | Glioblastoma multiforme | nervous_system | glioma | 0.005333 | 0.734444 | GDSC2 | -5.23384134736367 |
| AM-38 | GBM | Glioblastoma multiforme | nervous_system | glioma | 0.00621 | 0.779108 | GDSC2 | -5.08159438303675 |
| YH-13 | GBM | Glioblastoma multiforme | nervous_system | glioma | 0.006247 | 0.775195 | GDSC2 | -5.0756539304707 |
| GB-1 | GBM | Glioblastoma multiforme | nervous_system | glioma | 0.006492 | 0.738658 | GDSC2 | -5.03718462932979 |
| SF268 | GBM | Glioblastoma multiforme | nervous_system | glioma | 0.008948 | 0.788676 | GDSC2 | -4.71632523535425 |
| 42-MG-BA | GBM | Glioblastoma multiforme | nervous_system | glioma | 0.009528 | 0.810545 | GDSC2 | -4.65352044692914 |
| KS-1 | GBM | Glioblastoma multiforme | nervous_system | glioma | 0.009866 | 0.813131 | GDSC2 | -4.61866077617059 |
| U251 | GBM | Glioblastoma multiforme | nervous_system | glioma | 0.012163 | 0.808798 | GDSC2 | -4.40935672234584 |
| CAS-1 | GBM | Glioblastoma multiforme | nervous_system | glioma | 0.013014 | 0.795207 | GDSC2 | -4.34172957790934 |
| U-87-MG | GBM | Glioblastoma multiforme | nervous_system | glioma | 0.013508 | 0.828869 | GDSC2 | -4.30447317645882 |
| LN-405 | GBM | Glioblastoma multiforme | nervous_system | glioma | 0.016996 | 0.835918 | GDSC2 | -4.07477725672957 |
| Becker | GBM | Glioblastoma multiforme | nervous_system | glioma | 0.027807 | 0.873683 | GDSC2 | -3.58246749142033 |
| DBTRG-05MG | GBM | Glioblastoma multiforme | nervous_system | glioma | 0.03818 | 0.815545 | GDSC2 | -3.26544346068454 |
| D-542MG | GBM | Glioblastoma multiforme | nervous_system | glioma | 0.000363 | 0.322606 | GDSC1 | -7.92110772369942 |
| D-247MG | GBM | Glioblastoma multiforme | nervous_system | glioma | 0.000469 | 0.36343 | GDSC1 | -7.66490778951799 |
| SF126 | GBM | Glioblastoma multiforme | nervous_system | glioma | 0.000673 | 0.404076 | GDSC1 | -7.30376522831955 |
| SNB75 | GBM | Glioblastoma multiforme | nervous_system | glioma | 0.001342 | 0.509801 | GDSC1 | -6.61359424043265 |
| CAL-27 | HNSC | Head and Neck squamous cell car | aero_digestive_tract | head and neck | 0.002861 | 0.685853 | GDSC2 | -5.85658406491416 |
| HO-1-u-1 | HNSC | Head and Neck squamous cell car | aero_digestive_tract | head and neck | 0.003146 | 0.697292 | GDSC2 | -5.76162347434605 |
| SAS | HNSC | Head and Neck squamous cell car | aero_digestive_tract | head and neck | 0.003578 | 0.712848 | GDSC2 | -5.63295129389561 |
| LB771-HNC | HNSC | Head and Neck squamous cell car | aero_digestive_tract | head and neck | 0.004287 | 0.704847 | GDSC2 | -5.4521680913663 |
| Ca9-22 | HNSC | Head and Neck squamous cell car | aero_digestive_tract | head and neck | 0.0048 | 0.746006 | GDSC2 | -5.33913936106829 |
| OSC-20 | HNSC | Head and Neck squamous cell car | aero_digestive_tract | head and neck | 0.005018 | 0.722522 | GDSC2 | -5.29472383103791 |
| Detroit562 | HNSC | Head and Neck squamous cell car | aero_digestive_tract | head and neck | 0.005094 | 0.760599 | GDSC2 | -5.27969190242487 |
| HO-1-N-1 | HNSC | Head and Neck squamous cell car | aero_digestive_tract | head and neck | 0.005183 | 0.765745 | GDSC2 | -5.26237123977457 |
| SKN-3 | HNSC | Head and Neck squamous cell car | aero_digestive_tract | head and neck | 0.005252 | 0.749288 | GDSC2 | -5.24914632254159 |
| HSC-2 | HNSC | Head and Neck squamous cell car | aero_digestive_tract | head and neck | 0.00558 | 0.786253 | GDSC2 | -5.18856650258892 |
| CAL-33 | HNSC | Head and Neck squamous cell car | aero_digestive_tract | head and neck | 0.006694 | 0.778115 | GDSC2 | -5.006543676193 |
| BB49-HNC | HNSC | Head and Neck squamous cell car | aero_digestive_tract | head and neck | 0.007881 | 0.773804 | GDSC2 | -4.84330047961062 |
| JHU-022 | HNSC | Head and Neck squamous cell car | aero_digestive_tract | head and neck | 0.008361 | 0.751417 | GDSC2 | -4.78417724181422 |
| SCC-15 | HNSC | Head and Neck squamous cell car | aero_digestive_tract | head and neck | 0.008453 | 0.781982 | GDSC2 | -4.77323387103535 |
| HSC-3 | HNSC | Head and Neck squamous cell car | aero_digestive_tract | head and neck | 0.010237 | 0.821467 | GDSC2 | -4.5817466710445 |
| FADU | HNSC | Head and Neck squamous cell car | aero_digestive_tract | head and neck | 0.010255 | 0.819106 | GDSC2 | -4.57998988745779 |
| BICR10 | HNSC | Head and Neck squamous cell car | aero_digestive_tract | head and neck | 0.010758 | 0.800176 | GDSC2 | -4.53210561513109 |
| BICR22 | HNSC | Head and Neck squamous cell car | aero_digestive_tract | head and neck | 0.011485 | 0.841421 | GDSC2 | -4.46671344284108 |
| PCI-30 | HNSC | Head and Neck squamous cell car | aero_digestive_tract | head and neck | 0.012456 | 0.795274 | GDSC2 | -4.38555284445044 |
| SCC-4 | HNSC | Head and Neck squamous cell car | aero_digestive_tract | head and neck | 0.013012 | 0.846131 | GDSC2 | -4.34188327037103 |
| BICR78 | HNSC | Head and Neck squamous cell car | aero_digestive_tract | head and neck | 0.013203 | 0.807269 | GDSC2 | -4.32731120248507 |
| SCC-9 | HNSC | Head and Neck squamous cell car | aero_digestive_tract | head and neck | 0.013703 | 0.836424 | GDSC2 | -4.29014049201807 |
| SCC90 | HNSC | Head and Neck squamous cell car | aero_digestive_tract | head and neck | 0.016934 | 0.823414 | GDSC2 | -4.07843184376205 |
| HSC-4 | HNSC | Head and Neck squamous cell car | aero_digestive_tract | head and neck | 0.0171 | 0.872359 | GDSC2 | -4.06867681547352 |
| DOK | HNSC | Head and Neck squamous cell car | aero_digestive_tract | head and neck | 0.018329 | 0.860026 | GDSC2 | -3.99927077399243 |
| SAT | HNSC | Head and Neck squamous cell car | aero_digestive_tract | head and neck | 0.036649 | 0.888542 | GDSC2 | -3.30636913615133 |
| BB30-HNC | HNSC | Head and Neck squamous cell car | aero_digestive_tract | head and neck | 0.000687 | 0.405308 | GDSC1 | -7.28317626574192 |
| A704 | KIRC | Kidney renal clear cell carcinoma | kidney | kidney | 0.001266 | 0.588401 | GDSC2 | -6.67189295526015 |
| A498 | KIRC | Kidney renal clear cell carcinoma | kidney | kidney | 0.001952 | 0.627781 | GDSC2 | -6.23890079099124 |
| KMRC-1 | KIRC | Kidney renal clear cell carcinoma | kidney | kidney | 0.004468 | 0.724878 | GDSC2 | -5.41081439777518 |
| 786-0 | KIRC | Kidney renal clear cell carcinoma | kidney | kidney | 0.005006 | 0.737811 | GDSC2 | -5.29711808597255 |
| BFTC-909 | KIRC | Kidney renal clear cell carcinoma | kidney | kidney | 0.005078 | 0.751235 | GDSC2 | -5.28283779569965 |
| KMRC-20 | KIRC | Kidney renal clear cell carcinoma | kidney | kidney | 0.00551 | 0.752868 | GDSC2 | -5.20119065581731 |
| ACHN | KIRC | Kidney renal clear cell carcinoma | kidney | kidney | 0.005885 | 0.736045 | GDSC2 | -5.1353485382699 |
| CAL-54 | KIRC | Kidney renal clear cell carcinoma | kidney | kidney | 0.007402 | 0.756133 | GDSC2 | -4.90600504501817 |
| 769-P | KIRC | Kidney renal clear cell carcinoma | kidney | kidney | 0.009361 | 0.803772 | GDSC2 | -4.67120315659253 |
| LB996-RCC | KIRC | Kidney renal clear cell carcinoma | kidney | kidney | 0.010748 | 0.782721 | GDSC2 | -4.53303558822889 |
| VMRC-RCZ | KIRC | Kidney renal clear cell carcinoma | kidney | kidney | 0.011432 | 0.755575 | GDSC2 | -4.47133883835457 |
| OS-RC-2 | KIRC | Kidney renal clear cell carcinoma | kidney | kidney | 0.013818 | 0.809929 | GDSC2 | -4.28178318891553 |
| RCC-JW | KIRC | Kidney renal clear cell carcinoma | kidney | kidney | 0.014378 | 0.837758 | GDSC2 | -4.24205601842046 |
| RCC10RGB | KIRC | Kidney renal clear cell carcinoma | kidney | kidney | 0.014844 | 0.769857 | GDSC2 | -4.21015953578379 |
| TK10 | KIRC | Kidney renal clear cell carcinoma | kidney | kidney | 0.017146 | 0.788746 | GDSC2 | -4.06599036872557 |
| SN12C | KIRC | Kidney renal clear cell carcinoma | kidney | kidney | 0.051792 | 0.901621 | GDSC2 | -2.96051958179825 |
| BB65-RCC | KIRC | Kidney renal clear cell carcinoma | kidney | kidney | 0.000294 | 0.298113 | GDSC1 | -8.13193079062559 |
| RXF393 | KIRC | Kidney renal clear cell carcinoma | kidney | kidney | 0.000451 | 0.367309 | GDSC1 | -7.7040432184616 |
| LB2241-RCC | KIRC | Kidney renal clear cell carcinoma | kidney | kidney | 0.000969 | 0.461388 | GDSC1 | -6.93924594607351 |
| HA7-RCC | KIRC | Kidney renal clear cell carcinoma | kidney | kidney | 0.001159 | 0.48867 | GDSC1 | -6.76019771462452 |
| LB1047-RCC | KIRC | Kidney renal clear cell carcinoma | kidney | kidney | 0.002151 | 0.581326 | GDSC1 | -6.14182242869654 |
| OCI-M1 | LAML | Acute Myeloid Leukemia | blood | acute myeloid leukaemia | 0.000874 | 0.545062 | GDSC2 | -7.04243018230874 |
| ML-2 | LAML | Acute Myeloid Leukemia | blood | acute myeloid leukaemia | 0.002458 | 0.690042 | GDSC2 | -6.00840726783829 |
| MOLM-13 | LAML | Acute Myeloid Leukemia | blood | acute myeloid leukaemia | 0.002507 | 0.692965 | GDSC2 | -5.98866845980598 |
| GDM-1 | LAML | Acute Myeloid Leukemia | blood | acute myeloid leukaemia | 0.002736 | 0.680765 | GDSC2 | -5.90125827922183 |
| KMOE-2 | LAML | Acute Myeloid Leukemia | blood | acute myeloid leukaemia | 0.003122 | 0.713566 | GDSC2 | -5.7692814568889 |
| MONO-MAC-6 | LAML | Acute Myeloid Leukemia | blood | acute myeloid leukaemia | 0.003331 | 0.723301 | GDSC2 | -5.70448271977059 |
| KY821 | LAML | Acute Myeloid Leukemia | blood | leukemia | 0.003515 | 0.735511 | GDSC2 | -5.65071575371951 |

### Supplementary Table 1. Merged GDSC1 and GDSC2 datasets (860 cell lines; duplicates were excluded from GDSC2 datasets)

| Sample name | TCGA | TCGA organ system | Organ system | Organ system subtype | IC50 | AUC | Dataset | LN_IC50 |
|---|---|---|---|---|---|---|---|---|
| CTV-1 | LAML | Acute Myeloid Leukemia | blood | acute myeloid leukaemia | 0.003765 | 0.750818 | GDSC2 | -5.58200741773028 |
| HL-60 | LAML | Acute Myeloid Leukemia | blood | acute myeloid leukaemia | 0.004007 | 0.744758 | GDSC2 | -5.51971244732813 |
| ME-1 | LAML | Acute Myeloid Leukemia | blood | acute myeloid leukaemia | 0.004137 | 0.749192 | GDSC2 | -5.48778439150286 |
| OCI-AML2 | LAML | Acute Myeloid Leukemia | blood | acute myeloid leukaemia | 0.004142 | 0.765868 | GDSC2 | -5.48657651600874 |
| OCI-AML3 | LAML | Acute Myeloid Leukemia | blood | acute myeloid leukaemia | 0.004151 | 0.742859 | GDSC2 | -5.48440600991124 |
| KG-1 | LAML | Acute Myeloid Leukemia | blood | acute myeloid leukaemia | 0.004433 | 0.757596 | GDSC2 | -5.41867872321922 |
| CESS | LAML | Acute Myeloid Leukemia | blood | acute myeloid leukaemia | 0.004704 | 0.754841 | GDSC2 | -5.35934206838581 |
| NKM-1 | LAML | Acute Myeloid Leukemia | blood | acute myeloid leukaemia | 0.007584 | 0.806918 | GDSC2 | -4.88171451402942 |
| P31-FUJ | LAML | Acute Myeloid Leukemia | blood | acute myeloid leukaemia | 0.011831 | 0.850683 | GDSC2 | -4.43703207371061 |
| NOMO-1 | LAML | Acute Myeloid Leukemia | blood | acute myeloid leukaemia | 0.014831 | 0.879454 | GDSC2 | -4.21103569422231 |
| CMK | LAML | Acute Myeloid Leukemia | blood | acute myeloid leukaemia | 0.001275 | 0.50338 | GDSC1 | -6.66480910037175 |
| OCI-AML5 | LAML | Acute Myeloid Leukemia | blood | acute myeloid leukaemia | 0.001382 | 0.516723 | GDSC1 | -6.58422355363666 |
| HEL | LAML | Acute Myeloid Leukemia | blood | acute myeloid leukaemia | 0.001552 | 0.537307 | GDSC1 | -6.46821085722111 |
| QIMR-WIL | LAML | Acute Myeloid Leukemia | blood | acute myeloid leukaemia | 0.002122 | 0.583676 | GDSC1 | -6.15539623879035 |
| PL-21 | LAML | Acute Myeloid Leukemia | blood | acute myeloid leukaemia | 0.002666 | 0.607701 | GDSC1 | -5.92717605722562 |
| SIG-M5 | LAML | Acute Myeloid Leukemia | blood | acute myeloid leukaemia | 0.003572 | 0.686318 | GDSC1 | -5.63462961596788 |
| NB4 | LAML | Acute Myeloid Leukemia | blood | acute myeloid leukaemia | 0.004332 | 0.712057 | GDSC1 | -5.44172594984339 |
| KASUMI-1 | LAML | Acute Myeloid Leukemia | blood | acute myeloid leukaemia | 0.036053 | 0.914155 | GDSC1 | -3.32276520096046 |
| THP-1 | LAML | Acute Myeloid Leukemia | blood | acute myeloid leukaemia | 0.055733 | 0.961618 | GDSC1 | -2.88718284787877 |
| BV-173 | LCML | | blood | chronic myeloid leukaemia | 0.002597 | 0.694181 | GDSC2 | -5.95339834630153 |
| CML-T1 | LCML | | blood | chronic myeloid leukaemia | 0.002963 | 0.71657 | GDSC2 | -5.82155301039071 |
| KCL-22 | LCML | | blood | chronic myeloid leukaemia | 0.003654 | 0.733553 | GDSC2 | -5.61193282102632 |
| LAMA-84 | LCML | | blood | chronic myeloid leukaemia | 0.003877 | 0.751452 | GDSC2 | -5.55269362029335 |
| EM-2 | LCML | | blood | chronic myeloid leukaemia | 0.004264 | 0.749951 | GDSC2 | -5.45754759211859 |
| KU812 | LCML | | blood | chronic myeloid leukaemia | 0.004391 | 0.75906 | GDSC2 | -5.42819828740256 |
| MEG-01 | LCML | | blood | chronic myeloid leukaemia | 0.004554 | 0.758159 | GDSC2 | -5.39174931134059 |
| K-562 | LCML | | blood | chronic myeloid leukaemia | 0.014477 | 0.854279 | GDSC2 | -4.23519409581023 |
| RPMI-8866 | LCML | | blood | chronic myeloid leukaemia | 0.003421 | 0.661421 | GDSC1 | -5.67782237298667 |
| JURL-MK1 | LCML | | blood | chronic myeloid leukaemia | 0.004639 | 0.697339 | GDSC1 | -5.37325645321253 |
| MOG-G-CCM | LGG | Brain Lower Grade Glioma | nervous_system | glioma | 0.003039 | 0.676266 | GDSC2 | -5.79622676504748 |
| SF539 | LGG | Brain Lower Grade Glioma | nervous_system | glioma | 0.003543 | 0.728035 | GDSC2 | -5.6427814530988 |
| GAMG | LGG | Brain Lower Grade Glioma | nervous_system | glioma | 0.003964 | 0.731685 | GDSC2 | -5.5305016625144 |
| SW1783 | LGG | Brain Lower Grade Glioma | nervous_system | glioma | 0.004097 | 0.73259 | GDSC2 | -5.4975002804174 |
| MOG-G-UVW | LGG | Brain Lower Grade Glioma | nervous_system | glioma | 0.004381 | 0.749635 | GDSC2 | -5.43047827015038 |
| GI-1 | LGG | Brain Lower Grade Glioma | nervous_system | glioma | 0.004629 | 0.758431 | GDSC2 | -5.375414416933 |
| H4 | LGG | Brain Lower Grade Glioma | nervous_system | glioma | 0.006661 | 0.795346 | GDSC2 | -5.01148565555109 |
| KINGS-1 | LGG | Brain Lower Grade Glioma | nervous_system | glioma | 0.00978 | 0.763183 | GDSC2 | -4.62741579493541 |
| KALS-1 | LGG | Brain Lower Grade Glioma | nervous_system | glioma | 0.011158 | 0.805281 | GDSC2 | -4.4955985495588 |
| no-10 | LGG | Brain Lower Grade Glioma | nervous_system | glioma | 0.000213 | 0.25458 | GDSC1 | -8.45421839225485 |
| KNS-42 | LGG | Brain Lower Grade Glioma | nervous_system | glioma | 0.000329 | 0.30692 | GDSC1 | -8.0194528071989 |
| D-336MG | LGG | Brain Lower Grade Glioma | nervous_system | glioma | 0.000595 | 0.390355 | GDSC1 | -7.42694915241864 |
| KNS-81-FD | LGG | Brain Lower Grade Glioma | nervous_system | glioma | 0.001079 | 0.480504 | GDSC1 | -6.83172059270614 |
| no-11 | LGG | Brain Lower Grade Glioma | nervous_system | glioma | 0.00161 | 0.535751 | GDSC1 | -6.43152109998577 |
| NMC-G1 | LGG | Brain Lower Grade Glioma | nervous_system | glioma | 0.003295 | 0.637406 | GDSC1 | -5.71534911102767 |
| D-502MG | LGG | Brain Lower Grade Glioma | nervous_system | glioma | 0.006631 | 0.707184 | GDSC1 | -5.01599965659541 |
| JHH-1 | LIHC | Liver hepatocellular carcinoma | digestive_system | liver | 0.003758 | 0.709039 | GDSC2 | -5.58386837799086 |
| HLE | LIHC | Liver hepatocellular carcinoma | digestive_system | liver | 0.005209 | 0.76339 | GDSC2 | -5.25736738021908 |
| JHH-2 | LIHC | Liver hepatocellular carcinoma | digestive_system | liver | 0.007097 | 0.766594 | GDSC2 | -4.94808311943929 |
| SK-HEP-1 | LIHC | Liver hepatocellular carcinoma | digestive_system | liver | 0.008425 | 0.812364 | GDSC2 | -4.77655180274371 |
| JHH-4 | LIHC | Liver hepatocellular carcinoma | digestive_system | liver | 0.009825 | 0.78077 | GDSC2 | -4.62282512122681 |
| SNU-423 | LIHC | Liver hepatocellular carcinoma | digestive_system | liver | 0.010117 | 0.808289 | GDSC2 | -4.59353810175838 |
| SNU-387 | LIHC | Liver hepatocellular carcinoma | digestive_system | liver | 0.010385 | 0.790338 | GDSC2 | -4.56739282165406 |
| SNU-398 | LIHC | Liver hepatocellular carcinoma | digestive_system | liver | 0.012836 | 0.817455 | GDSC2 | -4.35550155573453 |
| SNU-182 | LIHC | Liver hepatocellular carcinoma | digestive_system | liver | 0.015505 | 0.813532 | GDSC2 | -4.16659272642972 |
| JHH-7 | LIHC | Liver hepatocellular carcinoma | digestive_system | liver | 0.021569 | 0.842544 | GDSC2 | -3.8364981803419 |
| SNU-449 | LIHC | Liver hepatocellular carcinoma | digestive_system | liver | 0.024875 | 0.845003 | GDSC2 | -3.69389199593748 |
| huH-1 | LIHC | Liver hepatocellular carcinoma | digestive_system | liver | 0.031976 | 0.857636 | GDSC2 | -3.44276965757311 |
| C3A | LIHC | Liver hepatocellular carcinoma | digestive_system | liver | 0.03695 | 0.864161 | GDSC2 | -3.29818963158793 |
| HuH-7 | LIHC | Liver hepatocellular carcinoma | digestive_system | liver | 0.048566 | 0.90202 | GDSC2 | -3.02483158137927 |
| JHH-6 | LIHC | Liver hepatocellular carcinoma | digestive_system | liver | 0.257712 | 0.948335 | GDSC2 | -1.35591259663381 |
| NCI-H1975 | LUAD | Lung adenocarcinoma | lung | lung NSCLC adenocarcinoma | 0.002676 | 0.663699 | GDSC2 | -5.92343213671615 |
| NCI-H1693 | LUAD | Lung adenocarcinoma | lung | lung NSCLC adenocarcinoma | 0.004011 | 0.670809 | GDSC2 | -5.51871469219422 |
| NCI-H1755 | LUAD | Lung adenocarcinoma | lung | lung NSCLC adenocarcinoma | 0.004498 | 0.721369 | GDSC2 | -5.40412242544501 |
| HOP-62 | LUAD | Lung adenocarcinoma | lung | lung NSCLC adenocarcinoma | 0.004672 | 0.725554 | GDSC2 | -5.36616803345621 |
| HCC-78 | LUAD | Lung adenocarcinoma | lung | lung NSCLC adenocarcinoma | 0.00502 | 0.720698 | GDSC2 | -5.2943253452785 |
| LXF-289 | LUAD | Lung adenocarcinoma | lung | lung NSCLC adenocarcinoma | 0.005396 | 0.780593 | GDSC2 | -5.22209734063663 |
| NCI-H2085 | LUAD | Lung adenocarcinoma | lung | lung NSCLC adenocarcinoma | 0.006663 | 0.761357 | GDSC2 | -5.01118544540174 |
| EMC-BAC-2 | LUAD | Lung adenocarcinoma | lung | lung NSCLC adenocarcinoma | 0.007918 | 0.786829 | GDSC2 | -4.8386166302982 |
| A549 | LUAD | Lung adenocarcinoma | lung | lung NSCLC adenocarcinoma | 0.008002 | 0.777521 | GDSC2 | -4.82806376854709 |
| HCC-827 | LUAD | Lung adenocarcinoma | lung | lung NSCLC adenocarcinoma | 0.008568 | 0.757412 | GDSC2 | -4.75972094583669 |
| NCI-H1781 | LUAD | Lung adenocarcinoma | lung | lung NSCLC adenocarcinoma | 0.009143 | 0.77137 | GDSC2 | -4.69476671979985 |
| PC-3_[JPC-3] | LUAD | Lung adenocarcinoma | lung | lung NSCLC adenocarcinoma | 0.009458 | 0.758594 | GDSC2 | -4.66089433476045 |
| NCI-H2009 | LUAD | Lung adenocarcinoma | lung | lung NSCLC adenocarcinoma | 0.010611 | 0.821056 | GDSC2 | -4.54586408009068 |
| NCI-H23 | LUAD | Lung adenocarcinoma | lung | lung NSCLC adenocarcinoma | 0.010785 | 0.830124 | GDSC2 | -4.52959899914102 |
| COR-L105 | LUAD | Lung adenocarcinoma | lung | lung NSCLC adenocarcinoma | 0.011372 | 0.782725 | GDSC2 | -4.47660108519338 |
| NCI-H1650 | LUAD | Lung adenocarcinoma | lung | lung NSCLC adenocarcinoma | 0.011513 | 0.745069 | GDSC2 | -4.46427844729061 |
| HCC-44 | LUAD | Lung adenocarcinoma | lung | lung NSCLC adenocarcinoma | 0.011778 | 0.831755 | GDSC2 | -4.44152189445976 |
| PC-14 | LUAD | Lung adenocarcinoma | lung | lung NSCLC adenocarcinoma | 0.011824 | 0.796146 | GDSC2 | -4.43762391477629 |
| NCI-H1648 | LUAD | Lung adenocarcinoma | lung | lung NSCLC adenocarcinoma | 0.013608 | 0.834392 | GDSC2 | -4.29709742388858 |
| NCI-H358 | LUAD | Lung adenocarcinoma | lung | lung NSCLC adenocarcinoma | 0.014082 | 0.808307 | GDSC2 | -4.2628578928852 |
| Calu-3 | LUAD | Lung adenocarcinoma | lung | lung NSCLC adenocarcinoma | 0.014273 | 0.807021 | GDSC2 | -4.24938563833451 |
| NCI-H2122 | LUAD | Lung adenocarcinoma | lung | lung NSCLC adenocarcinoma | 0.014318 | 0.874565 | GDSC2 | -4.24623779200814 |
| NCI-H522 | LUAD | Lung adenocarcinoma | lung | lung NSCLC adenocarcinoma | 0.014881 | 0.850958 | GDSC2 | -4.20767004753422 |
| NCI-H2228 | LUAD | Lung adenocarcinoma | lung | lung NSCLC adenocarcinoma | 0.017328 | 0.806595 | GDSC2 | -4.0554315887235 |
| Calu-6 | LUAD | Lung adenocarcinoma | lung | lung NSCLC adenocarcinoma | 0.020599 | 0.836461 | GDSC2 | -3.8825127480542 |
| NCI-H1993 | LUAD | Lung adenocarcinoma | lung | lung NSCLC adenocarcinoma | 0.021099 | 0.866449 | GDSC2 | -3.85852963298815 |
| NCI-H650 | LUAD | Lung adenocarcinoma | lung | lung NSCLC adenocarcinoma | 0.022386 | 0.884916 | GDSC2 | -3.79931951551506 |
| RERF-LC-KJ | LUAD | Lung adenocarcinoma | lung | lung NSCLC adenocarcinoma | 0.023658 | 0.867913 | GDSC2 | -3.74405395485859 |
| NCI-H2087 | LUAD | Lung adenocarcinoma | lung | lung NSCLC adenocarcinoma | 0.025953 | 0.865605 | GDSC2 | -3.65146806911579 |
| NCI-H322M | LUAD | Lung adenocarcinoma | lung | lung NSCLC adenocarcinoma | 0.029542 | 0.862627 | GDSC2 | -3.52194229936373 |
| NCI-H1792 | LUAD | Lung adenocarcinoma | lung | lung NSCLC adenocarcinoma | 0.032033 | 0.936816 | GDSC2 | -3.4409886575554 |
| NCI-H1355 | LUAD | Lung adenocarcinoma | lung | lung NSCLC adenocarcinoma | 0.032928 | 0.858909 | GDSC2 | -3.4134319193305 |
| NCI-H2347 | LUAD | Lung adenocarcinoma | lung | lung NSCLC adenocarcinoma | 0.033748 | 0.807218 | GDSC2 | -3.38883412267876 |
| NCI-H1666 | LUAD | Lung adenocarcinoma | lung | lung NSCLC adenocarcinoma | 0.037745 | 0.844761 | GDSC2 | -3.27690226239055 |
| SK-LU-1 | LUAD | Lung adenocarcinoma | lung | lung NSCLC adenocarcinoma | 0.037939 | 0.858073 | GDSC2 | -3.27177567222906 |
| NCI-H2023 | LUAD | Lung adenocarcinoma | lung | lung NSCLC adenocarcinoma | 0.046949 | 0.906592 | GDSC2 | -3.05869337280922 |
| NCI-H1623 | LUAD | Lung adenocarcinoma | lung | lung NSCLC adenocarcinoma | 0.047709 | 0.885507 | GDSC2 | -3.04263521964025 |
| NCI-H3122 | LUAD | Lung adenocarcinoma | lung | lung NSCLC adenocarcinoma | 0.047825 | 0.900335 | GDSC2 | -3.04020676365582 |
| NCI-H1573 | LUAD | Lung adenocarcinoma | lung | lung NSCLC adenocarcinoma | 0.049413 | 0.900283 | GDSC2 | -3.00754173151349 |
| NCI-H1651 | LUAD | Lung adenocarcinoma | lung | lung NSCLC adenocarcinoma | 0.06317 | 0.941933 | GDSC2 | -2.76192577407153 |

**Supplementary Table 1. Merged GDSC1 and GDSC2 datasets (860 cell lines; duplicates were excluded from GDSC2 datasets)**

| Sample name | TCGA | TCGA organ system | Organ system | Organ system subtype | IC50 | AUC | Dataset | LN_IC50 |
|---|---|---|---|---|---|---|---|---|
| NCI-H2342 | LUAD | Lung adenocarcinoma | lung | lung NSCLC adenocarcinoma | 0.072552 | 0.921352 | GDSC2 | -2.62345173283687 |
| NCI-H1563 | LUAD | Lung adenocarcinoma | lung | lung NSCLC adenocarcinoma | 0.080912 | 0.925361 | GDSC2 | -2.51439313464451 |
| SW1573 | LUAD | Lung adenocarcinoma | lung | lung NSCLC adenocarcinoma | 0.081928 | 0.892679 | GDSC2 | -2.501914466209 |
| NCI-H2405 | LUAD | Lung adenocarcinoma | lung | lung NSCLC adenocarcinoma | 0.100375 | 0.851055 | GDSC2 | -2.29884210671521 |
| NCI-H1568 | LUAD | Lung adenocarcinoma | lung | lung NSCLC adenocarcinoma | 0.114223 | 0.894811 | GDSC2 | -2.16960260098791 |
| NCI-H1944 | LUAD | Lung adenocarcinoma | lung | lung NSCLC adenocarcinoma | 0.121007 | 0.921337 | GDSC2 | -2.11190688381904 |
| VMRC-LCD | LUAD | Lung adenocarcinoma | lung | lung NSCLC adenocarcinoma | 0.260655 | 0.968984 | GDSC2 | -1.34455758513981 |
| NCI-H1435 | LUAD | Lung adenocarcinoma | lung | lung NSCLC adenocarcinoma | 0.335284 | 0.993337 | GDSC2 | -1.0927773451096 |
| NCI-H1838 | LUAD | Lung adenocarcinoma | lung | lung NSCLC adenocarcinoma | 0.416641 | 0.937982 | GDSC2 | -0.875530339251258 |
| NCI-H1793 | LUAD | Lung adenocarcinoma | lung | lung NSCLC adenocarcinoma | 0.64715 | 0.939308 | GDSC2 | -0.43517717209369 |
| NCI-H441 | LUAD | Lung adenocarcinoma | lung | lung NSCLC adenocarcinoma | 0.842319 | 0.916848 | GDSC2 | -0.171596476631603 |
| LC-2-ad | LUAD | Lung adenocarcinoma | lung | lung NSCLC adenocarcinoma | 0.001316 | 0.508191 | GDSC1 | -6.63315844607901 |
| EKVX | LUAD | Lung adenocarcinoma | lung | lung NSCLC adenocarcinoma | 0.002275 | 0.568887 | GDSC1 | -6.08577522657922 |
| NCI-H1869 | LUSC | Lung squamous cell carcinoma | lung | lung NSCLC squamous cell carcinoma | 0.002252 | 0.643331 | GDSC2 | -6.09593656870469 |
| LOU-NH91 | LUSC | Lung squamous cell carcinoma | lung | lung NSCLC squamous cell carcinoma | 0.002614 | 0.665886 | GDSC2 | -5.94687366378011 |
| EBC-1 | LUSC | Lung squamous cell carcinoma | lung | lung NSCLC squamous cell carcinoma | 0.003115 | 0.67795 | GDSC2 | -5.77152612674272 |
| HARA | LUSC | Lung squamous cell carcinoma | lung | lung NSCLC squamous cell carcinoma | 0.004429 | 0.742418 | GDSC2 | -5.41958145404108 |
| LC-1-sq | LUSC | Lung squamous cell carcinoma | lung | lung NSCLC squamous cell carcinoma | 0.008502 | 0.794716 | GDSC2 | -4.76745384904554 |
| SW900 | LUSC | Lung squamous cell carcinoma | lung | lung NSCLC squamous cell carcinoma | 0.008649 | 0.750322 | GDSC2 | -4.75031157165776 |
| NCI-H2170 | LUSC | Lung squamous cell carcinoma | lung | lung NSCLC squamous cell carcinoma | 0.009203 | 0.803409 | GDSC2 | -4.68822576112542 |
| HCC-15 | LUSC | Lung squamous cell carcinoma | lung | lung NSCLC squamous cell carcinoma | 0.009872 | 0.794076 | GDSC2 | -4.6180528118191 |
| KNS-62 | LUSC | Lung squamous cell carcinoma | lung | lung NSCLC squamous cell carcinoma | 0.010306 | 0.816019 | GDSC2 | -4.5750290290761 |
| RERF-LC-Sq1 | LUSC | Lung squamous cell carcinoma | lung | lung NSCLC squamous cell carcinoma | 0.010725 | 0.802643 | GDSC2 | -4.53517781416806 |
| NCI-H520 | LUSC | Lung squamous cell carcinoma | lung | lung NSCLC squamous cell carcinoma | 0.011228 | 0.806179 | GDSC2 | -4.4893446204825 |
| EPLC-272H | LUSC | Lung squamous cell carcinoma | lung | lung NSCLC squamous cell carcinoma | 0.01219 | 0.819277 | GDSC2 | -4.40713933548896 |
| LK-2 | LUSC | Lung squamous cell carcinoma | lung | lung NSCLC squamous cell carcinoma | 0.023801 | 0.866854 | GDSC2 | -3.73802768238067 |
| SK-MES-1 | LUSC | Lung squamous cell carcinoma | lung | lung NSCLC squamous cell carcinoma | 0.072725 | 0.929796 | GDSC2 | -2.62107007460087 |
| NCI-H226 | LUSC | Lung squamous cell carcinoma | lung | lung NSCLC squamous cell carcinoma | 0.121788 | 0.877422 | GDSC2 | -2.10547345072746 |
| PFSK-1 | MB | | nervous_system | medulloblastoma | 0.002202 | 0.663749 | GDSC2 | -6.11838924068165 |
| D-283MED | MB | | nervous_system | medulloblastoma | 0.002781 | 0.699194 | GDSC2 | -5.88494470373031 |
| Daoy | MB | | nervous_system | medulloblastoma | 0.004877 | 0.760941 | GDSC2 | -5.32322500225422 |
| ONS-76 | MB | | nervous_system | medulloblastoma | 0.005771 | 0.756409 | GDSC2 | -5.15490990325331 |
| NCI-H2052 | MESO | Mesothelioma | lung | mesothelioma | 0.004852 | 0.722088 | GDSC2 | -5.32836428790033 |
| NCI-H2818 | MESO | Mesothelioma | lung | mesothelioma | 0.004941 | 0.718581 | GDSC2 | -5.31018753911852 |
| NCI-H2731 | MESO | Mesothelioma | lung | mesothelioma | 0.005199 | 0.744104 | GDSC2 | -5.25928897958056 |
| NCI-H2795 | MESO | Mesothelioma | lung | mesothelioma | 0.0052 | 0.748744 | GDSC2 | -5.25909665339476 |
| NCI-H2373 | MESO | Mesothelioma | lung | mesothelioma | 0.006533 | 0.73249 | GDSC2 | -5.03088902312352 |
| NCI-H2369 | MESO | Mesothelioma | lung | mesothelioma | 0.009082 | 0.790196 | GDSC2 | -4.70146084630638 |
| NCI-H2452 | MESO | Mesothelioma | lung | mesothelioma | 0.010671 | 0.773789 | GDSC2 | -4.54022549734771 |
| NCI-H513 | MESO | Mesothelioma | lung | mesothelioma | 0.013451 | 0.781504 | GDSC2 | -4.30870182625569 |
| MPP-89 | MESO | Mesothelioma | lung | mesothelioma | 0.015976 | 0.854563 | GDSC2 | -4.13666768286862 |
| NCI-H2722 | MESO | Mesothelioma | lung | mesothelioma | 0.017461 | 0.766252 | GDSC2 | -4.04778545644215 |
| NCI-H2591 | MESO | Mesothelioma | lung | mesothelioma | 0.01844 | 0.785056 | GDSC2 | -3.99323306085369 |
| MSTO-211H | MESO | Mesothelioma | lung | mesothelioma | 0.021789 | 0.854173 | GDSC2 | -3.82635002369 |
| NCI-H2461 | MESO | Mesothelioma | lung | mesothelioma | 0.031858 | 0.855325 | GDSC2 | -3.44646675110969 |
| IST-MES1 | MESO | Mesothelioma | lung | mesothelioma | 0.041757 | 0.8897 | GDSC2 | -3.17588817706876 |
| NCI-H28 | MESO | Mesothelioma | lung | mesothelioma | 0.130786 | 0.889969 | GDSC2 | -2.03419287932669 |
| NCI-H2595 | MESO | Mesothelioma | lung | mesothelioma | 0.372021 | 0.95654 | GDSC2 | -0.98880497468942 |
| AMO-1 | MM | | blood | haematopoietic neoplasm other | 0.000804 | 0.533148 | GDSC2 | -7.12591128878531 |
| MC-CAR | MM | | blood | haematopoietic neoplasm other | 0.001779 | 0.643234 | GDSC2 | -6.33170387029844 |
| ARH-77 | MM | | blood | myeloma | 0.002626 | 0.693683 | GDSC2 | -5.94229350310153 |
| JJN-3 | MM | | blood | haematopoietic neoplasm other | 0.00268 | 0.704499 | GDSC2 | -5.92193848445937 |
| MM1S | MM | | blood | myeloma | 0.002993 | 0.717157 | GDSC2 | -5.81147905011157 |
| NCI-H929 | MM | | blood | myeloma | 0.003066 | 0.714506 | GDSC2 | -5.78738149853251 |
| KMS-12-BM | MM | | blood | myeloma | 0.003253 | 0.727718 | GDSC2 | -5.72817763149092 |
| RPMI-8226 | MM | | blood | myeloma | 0.003381 | 0.729013 | GDSC2 | -5.68958375525639 |
| OPM-2 | MM | | blood | myeloma | 0.003564 | 0.743457 | GDSC2 | -5.63687176937357 |
| IM-9 | MM | | blood | myeloma | 0.003589 | 0.744112 | GDSC2 | -5.62988166681667 |
| U-266 | MM | | blood | myeloma | 0.004207 | 0.740316 | GDSC2 | -5.47100547437375 |
| EJM | MM | | blood | lymphoid neoplasm other | 0.005253 | 0.769321 | GDSC2 | -5.24895593701031 |
| SK-MM-2 | MM | | blood | myeloma | 0.005612 | 0.776226 | GDSC2 | -5.18284811674192 |
| KARPAS-620 | MM | | blood | haematopoietic neoplasm other | 0.005867 | 0.786774 | GDSC2 | -5.13841184903841 |
| L-363 | MM | | blood | myeloma | 0.00745 | 0.825775 | GDSC2 | -4.89954124659067 |
| MOLP-8 | MM | | blood | myeloma | 0.009333 | 0.839837 | GDSC2 | -4.67419877239853 |
| LP-1 | MM | | blood | myeloma | 0.003336 | 0.667363 | GDSC1 | -5.70298279448564 |
| KELLY | NB | | nervous_system | neuroblastoma | 0.002088 | 0.665523 | GDSC2 | -6.17154860896174 |
| NB13 | NB | | nervous_system | neuroblastoma | 0.003 | 0.708122 | GDSC2 | -5.80914299031403 |
| CHP-134 | NB | | nervous_system | neuroblastoma | 0.003438 | 0.71163 | GDSC2 | -5.67286537202148 |
| IMR-5 | NB | | nervous_system | neuroblastoma | 0.003592 | 0.741001 | GDSC2 | -5.62904612854218 |
| BE2-M17 | NB | | nervous_system | neuroblastoma | 0.003603 | 0.705912 | GDSC2 | -5.62598844721618 |
| NB69 | NB | | nervous_system | neuroblastoma | 0.003988 | 0.72541 | GDSC2 | -5.52446542688255 |
| NB14 | NB | | nervous_system | neuroblastoma | 0.004063 | 0.748052 | GDSC2 | -5.50583366197655 |
| SIMA | NB | | nervous_system | neuroblastoma | 0.004381 | 0.731125 | GDSC2 | -5.43047827015038 |
| SK-N-AS | NB | | nervous_system | neuroblastoma | 0.005112 | 0.735456 | GDSC2 | -5.2761645619069 |
| SK-N-DZ | NB | | nervous_system | neuroblastoma | 0.005295 | 0.753518 | GDSC2 | -5.24099229992877 |
| CHP-212 | NB | | nervous_system | neuroblastoma | 0.005394 | 0.754959 | GDSC2 | -5.2224680542646 |
| NB(TU)1-10 | NB | | nervous_system | neuroblastoma | 0.007638 | 0.800904 | GDSC2 | -4.87461949017881 |
| SK-N-FI | NB | | nervous_system | neuroblastoma | 0.007932 | 0.79374 | GDSC2 | -4.83685006832459 |
| NB1 | NB | | nervous_system | neuroblastoma | 0.008323 | 0.758297 | GDSC2 | -4.78873251221818 |
| TGW | NB | | nervous_system | neuroblastoma | 0.009135 | 0.819976 | GDSC2 | -4.69564208915217 |
| GI-ME-N | NB | | nervous_system | neuroblastoma | 0.011412 | 0.794689 | GDSC2 | -4.47308984563088 |
| SK-N-SH | NB | | nervous_system | neuroblastoma | 0.012147 | 0.801066 | GDSC2 | -4.410673053264 |
| NB7 | NB | | nervous_system | neuroblastoma | 0.012153 | 0.837936 | GDSC2 | -4.41017922609347 |
| KP-N-YN | NB | | nervous_system | neuroblastoma | 0.012896 | 0.825234 | GDSC2 | -4.35083809321786 |
| NB12 | NB | | nervous_system | neuroblastoma | 0.015019 | 0.842813 | GDSC2 | -4.19843921275869 |
| GOTO | NB | | nervous_system | neuroblastoma | 0.016838 | 0.853144 | GDSC2 | -4.08411704206666 |
| NH-12 | NB | | nervous_system | neuroblastoma | 0.018163 | 0.845708 | GDSC2 | -4.00836872121483 |
| NB10 | NB | | nervous_system | neuroblastoma | 0.018831 | 0.883359 | GDSC2 | -3.97225083097863 |
| LAN-6 | NB | | nervous_system | neuroblastoma | 0.002104 | 0.577413 | GDSC1 | -6.16391498410667 |
| NB5 | NB | | nervous_system | neuroblastoma | 0.00499 | 0.714693 | GDSC1 | -5.30031936921871 |
| CHP-126 | NB | | nervous_system | neuroblastoma | 0.006206 | 0.735319 | GDSC1 | -5.08223871295595 |
| MHH-NB-11 | NB | | nervous_system | neuroblastoma | 0.013394 | 0.817322 | GDSC1 | -4.31294843349428 |
| NB6 | NB | | nervous_system | neuroblastoma | 0.021219 | 0.850752 | GDSC1 | -3.85285827226017 |
| KP-N-YS | NB | | nervous_system | neuroblastoma | 0.027846 | 0.89062 | GDSC1 | -3.58106594949504 |
| TOV-21G | OV | Ovarian serous cystadenocarcinoı | urogenital_system | ovary | 0.002824 | 0.669674 | GDSC2 | -5.86960095935114 |
| Caov-4 | OV | Ovarian serous cystadenocarcinoı | urogenital_system | ovary | 0.004065 | 0.70758 | GDSC2 | -5.50534153598236 |
| OC-314 | OV | Ovarian serous cystadenocarcinoı | urogenital_system | ovary | 0.004946 | 0.762053 | GDSC2 | -5.30917610988291 |
| OAW-28 | OV | Ovarian serous cystadenocarcinoı | urogenital_system | ovary | 0.005017 | 0.724506 | GDSC2 | -5.29492313348002 |
| OVCAR-3 | OV | Ovarian serous cystadenocarcinoı | urogenital_system | ovary | 0.005867 | 0.755049 | GDSC2 | -5.13841184903841 |
| OVISE | OV | Ovarian serous cystadenocarcinoı | urogenital_system | ovary | 0.006795 | 0.76056 | GDSC2 | -4.99156823137903 |

**Supplementary Table 1. Merged GDSC1 and GDSC2 datasets (860 cell lines; duplicates were excluded from GDSC2 datasets)**

| Sample name | TCGA | TCGA organ system | Organ system | Organ system subtype | IC50 | AUC | Dataset | LN_IC50 |
|---|---|---|---|---|---|---|---|---|
| OVCAR-8 | OV | Ovarian serous cystadenocarcino | urogenital_system | ovary | 0.007322 | 0.803828 | GDSC2 | -4.91687176428409 |
| IGROV-1 | OV | Ovarian serous cystadenocarcino | urogenital_system | ovary | 0.008174 | 0.775296 | GDSC2 | -4.80679689384005 |
| SW626 | OV | Ovarian serous cystadenocarcino | urogenital_system | ovary | 0.008306 | 0.745589 | GDSC2 | -4.79077713377358 |
| OVCA420 | OV | Ovarian serous cystadenocarcino | urogenital_system | ovary | 0.009267 | 0.775297 | GDSC2 | -4.68129557637758 |
| OV-90 | OV | Ovarian serous cystadenocarcino | urogenital_system | ovary | 0.010321 | 0.77812 | GDSC2 | -4.57357462439834 |
| OVCAR-4 | OV | Ovarian serous cystadenocarcino | urogenital_system | ovary | 0.012765 | 0.810826 | GDSC2 | -4.36104822828869 |
| DOV13 | OV | Ovarian serous cystadenocarcino | urogenital_system | ovary | 0.012808 | 0.852203 | GDSC2 | -4.35768530328772 |
| OV-56 | OV | Ovarian serous cystadenocarcino | urogenital_system | ovary | 0.014719 | 0.807948 | GDSC2 | -4.21861610277169 |
| OVTOKO | OV | Ovarian serous cystadenocarcino | urogenital_system | ovary | 0.015114 | 0.81406 | GDSC2 | -4.19213381238361 |
| PEO1 | OV | Ovarian serous cystadenocarcino | urogenital_system | ovary | 0.016338 | 0.810064 | GDSC2 | -4.11426159606246 |
| Hey | OV | Ovarian serous cystadenocarcino | urogenital_system | ovary | 0.019581 | 0.82083 | GDSC2 | -3.93319557066095 |
| TOV-112D | OV | Ovarian serous cystadenocarcino | urogenital_system | ovary | 0.021414 | 0.893042 | GDSC2 | -3.84371036524606 |
| OVMIU | OV | Ovarian serous cystadenocarcino | urogenital_system | ovary | 0.022963 | 0.83294 | GDSC2 | -3.7738710540454 |
| OVCAR-5 | OV | Ovarian serous cystadenocarcino | urogenital_system | ovary | 0.023185 | 0.814486 | GDSC2 | -3.76424976113871 |
| SK-OV-3 | OV | Ovarian serous cystadenocarcino | urogenital_system | ovary | 0.027538 | 0.822957 | GDSC2 | -3.59218840995996 |
| OAW-42 | OV | Ovarian serous cystadenocarcino | urogenital_system | ovary | 0.033955 | 0.883702 | GDSC2 | -3.38271916041638 |
| FU-OV-1 | OV | Ovarian serous cystadenocarcino | urogenital_system | ovary | 0.041952 | 0.864179 | GDSC2 | -3.17122917140085 |
| EFO-21 | OV | Ovarian serous cystadenocarcino | urogenital_system | ovary | 0.064097 | 0.841564 | GDSC2 | -2.74735771802283 |
| OVKATE | OV | Ovarian serous cystadenocarcino | urogenital_system | ovary | 0.119285 | 0.885101 | GDSC2 | -2.12623969122848 |
| DAN-G | PAAD | Pancreatic adenocarcinoma | pancreas | pancreas | 0.004099 | 0.736429 | GDSC2 | -5.49701223745994 |
| PA-TU-8988T | PAAD | Pancreatic adenocarcinoma | pancreas | pancreas | 0.004108 | 0.709285 | GDSC2 | -5.49481898691583 |
| SW1990 | PAAD | Pancreatic adenocarcinoma | pancreas | pancreas | 0.004706 | 0.716295 | GDSC2 | -5.35891698867697 |
| CFPAC-1 | PAAD | Pancreatic adenocarcinoma | pancreas | pancreas | 0.005174 | 0.718478 | GDSC2 | -5.2641091952183 |
| KP-1N | PAAD | Pancreatic adenocarcinoma | pancreas | pancreas | 0.005208 | 0.735213 | GDSC2 | -5.25755937407587 |
| HuP-T3 | PAAD | Pancreatic adenocarcinoma | pancreas | pancreas | 0.005738 | 0.731083 | GDSC2 | -5.16064456142296 |
| HPAC | PAAD | Pancreatic adenocarcinoma | pancreas | pancreas | 0.007003 | 0.711444 | GDSC2 | -4.96141665030876 |
| PSN1 | PAAD | Pancreatic adenocarcinoma | pancreas | pancreas | 0.007822 | 0.781085 | GDSC2 | -4.85081500264881 |
| PANC-08-13 | PAAD | Pancreatic adenocarcinoma | pancreas | pancreas | 0.008819 | 0.764085 | GDSC2 | -4.73084679407609 |
| PANC-02-03 | PAAD | Pancreatic adenocarcinoma | pancreas | pancreas | 0.009825 | 0.764498 | GDSC2 | -4.62282512122681 |
| HPAF-II | PAAD | Pancreatic adenocarcinoma | pancreas | pancreas | 0.01038 | 0.775045 | GDSC2 | -4.56787440124439 |
| HuP-T4 | PAAD | Pancreatic adenocarcinoma | pancreas | pancreas | 0.010758 | 0.815146 | GDSC2 | -4.53210561513109 |
| PA-TU-8902 | PAAD | Pancreatic adenocarcinoma | pancreas | pancreas | 0.011378 | 0.775742 | GDSC2 | -4.47607361265368 |
| CAPAN-1 | PAAD | Pancreatic adenocarcinoma | pancreas | pancreas | 0.011995 | 0.799892 | GDSC2 | -4.42326538269048 |
| BxPC-3 | PAAD | Pancreatic adenocarcinoma | pancreas | pancreas | 0.012199 | 0.823165 | GDSC2 | -4.40640129781554 |
| KP-2 | PAAD | Pancreatic adenocarcinoma | pancreas | pancreas | 0.012474 | 0.811648 | GDSC2 | -4.38410880087821 |
| PL4 | PAAD | Pancreatic adenocarcinoma | pancreas | pancreas | 0.013212 | 0.813343 | GDSC2 | -4.32662977145359 |
| KP-4 | PAAD | Pancreatic adenocarcinoma | pancreas | pancreas | 0.014308 | 0.847392 | GDSC2 | -4.24693645758537 |
| PANC-10-05 | PAAD | Pancreatic adenocarcinoma | pancreas | pancreas | 0.014423 | 0.783242 | GDSC2 | -4.23893112438076 |
| YAPC | PAAD | Pancreatic adenocarcinoma | pancreas | pancreas | 0.016151 | 0.819152 | GDSC2 | -4.12577331172576 |
| PL18 | PAAD | Pancreatic adenocarcinoma | pancreas | pancreas | 0.018388 | 0.83691 | GDSC2 | -3.99605700103816 |
| MIA-PaCa-2 | PAAD | Pancreatic adenocarcinoma | pancreas | pancreas | 0.022961 | 0.893325 | GDSC2 | -3.77395815447225 |
| Hs-766T | PAAD | Pancreatic adenocarcinoma | pancreas | pancreas | 0.0242 | 0.797229 | GDSC2 | -3.7214026458195 |
| SUIT-2 | PAAD | Pancreatic adenocarcinoma | pancreas | pancreas | 0.032833 | 0.832972 | GDSC2 | -3.416321171808 |
| SU8686 | PAAD | Pancreatic adenocarcinoma | pancreas | pancreas | 0.051176 | 0.8449 | GDSC2 | -2.9724846068343 |
| PANC-03-27 | PAAD | Pancreatic adenocarcinoma | pancreas | pancreas | 0.06161 | 0.901678 | GDSC2 | -2.78693108395566 |
| CAPAN-2 | PAAD | Pancreatic adenocarcinoma | pancreas | pancreas | 0.065628 | 0.862094 | GDSC2 | -2.7237528448295 |
| PANC-04-03 | PAAD | Pancreatic adenocarcinoma | pancreas | pancreas | 0.187922 | 0.876575 | GDSC2 | -1.67172829586138 |
| AsPC-1 | PAAD | Pancreatic adenocarcinoma | pancreas | pancreas | 3.530379 | 0.965074 | GDSC2 | 1.26140523062104 |
| MZ1-PC | PAAD | Pancreatic adenocarcinoma | pancreas | pancreas | 0.003271 | 0.640386 | GDSC1 | -5.72265953042589 |
| PWR-1E | PRAD | Prostate adenocarcinoma | urogenital_system | prostate | 0.003884 | 0.731159 | GDSC2 | -5.55088972855306 |
| LNCaP-Clone-FGC | PRAD | Prostate adenocarcinoma | urogenital_system | prostate | 0.007491 | 0.765775 | GDSC2 | -4.89405297901639 |
| 22RV1 | PRAD | Prostate adenocarcinoma | urogenital_system | prostate | 0.00877 | 0.779146 | GDSC2 | -4.73641847259805 |
| PC-3 | PRAD | Prostate adenocarcinoma | urogenital_system | prostate | 0.019706 | 0.82274 | GDSC2 | -3.92683212108185 |
| VCaP | PRAD | Prostate adenocarcinoma | urogenital_system | prostate | 0.020624 | 0.854325 | GDSC2 | -3.88129983278531 |
| DU-145 | PRAD | Prostate adenocarcinoma | urogenital_system | prostate | 0.027068 | 0.885038 | GDSC2 | -3.60940306061216 |
| NCI-H1417 | SCLC | | lung | lung small cell carcinoma | 0.00137 | 0.601828 | GDSC2 | -6.5929445391421 |
| NCI-H446 | SCLC | | lung | lung small cell carcinoma | 0.002649 | 0.684285 | GDSC2 | -5.9335730686922 |
| NCI-H209 | SCLC | | lung | lung small cell carcinoma | 0.003259 | 0.714203 | GDSC2 | -5.72633487912671 |
| NCI-H2141 | SCLC | | lung | lung small cell carcinoma | 0.003646 | 0.707101 | GDSC2 | -5.61412460272567 |
| NCI-H69 | SCLC | | lung | lung small cell carcinoma | 0.003746 | 0.729672 | GDSC2 | -5.58706667496024 |
| NCI-H847 | SCLC | | lung | lung small cell carcinoma | 0.004028 | 0.747571 | GDSC2 | -5.51448530412582 |
| DMS-273 | SCLC | | lung | lung small cell carcinoma | 0.005063 | 0.764591 | GDSC2 | -5.28579608599436 |
| SHP-77 | SCLC | | lung | lung small cell carcinoma | 0.005225 | 0.777429 | GDSC2 | -5.25430048113126 |
| SBC-3 | SCLC | | lung | lung small cell carcinoma | 0.005945 | 0.780462 | GDSC2 | -5.12520474883939 |
| IST-SL2 | SCLC | | lung | lung small cell carcinoma | 0.006048 | 0.7746 | GDSC2 | -5.1080276401049 |
| NCI-H1963 | SCLC | | lung | lung small cell carcinoma | 0.006346 | 0.763925 | GDSC2 | -5.05993058582113 |
| SBC-5 | SCLC | | lung | lung small cell carcinoma | 0.006801 | 0.802028 | GDSC2 | -4.99068561878864 |
| NCI-H524 | SCLC | | lung | lung small cell carcinoma | 0.007276 | 0.8257 | GDSC2 | -4.92317401832626 |
| NCI-H1092 | SCLC | | lung | lung small cell carcinoma | 0.00756 | 0.775036 | GDSC2 | -4.8848840887907 |
| DMS-114 | SCLC | | lung | lung small cell carcinoma | 0.008627 | 0.804389 | GDSC2 | -4.7528584588877 |
| COR-L311 | SCLC | | lung | lung small cell carcinoma | 0.009863 | 0.848777 | GDSC2 | -4.61896489701028 |
| NCI-H82 | SCLC | | lung | lung small cell carcinoma | 0.009908 | 0.864549 | GDSC2 | -4.61441276735502 |
| CPC-N | SCLC | | lung | lung small cell carcinoma | 0.011656 | 0.853344 | GDSC2 | -4.45193421008923 |
| LU-135 | SCLC | | lung | lung small cell carcinoma | 0.011968 | 0.844038 | GDSC2 | -4.42551885775002 |
| NCI-H526 | SCLC | | lung | lung small cell carcinoma | 0.012127 | 0.858829 | GDSC2 | -4.41232090730748 |
| NCI-H1048 | SCLC | | lung | lung small cell carcinoma | 0.012353 | 0.839077 | GDSC2 | -4.39385633042746 |
| COR-L32 | SCLC | | lung | lung small cell carcinoma | 0.012769 | 0.84099 | GDSC2 | -4.36073492053959 |
| NCI-H1694 | SCLC | | lung | lung small cell carcinoma | 0.013896 | 0.849764 | GDSC2 | -4.27615425004333 |
| NCI-H1341 | SCLC | | lung | lung small cell carcinoma | 0.016187 | 0.827495 | GDSC2 | -4.12354682803031 |
| COR-L279 | SCLC | | lung | lung small cell carcinoma | 0.019044 | 0.876681 | GDSC2 | -3.96100318764986 |
| NCI-H841 | SCLC | | lung | lung small cell carcinoma | 0.019202 | 0.850948 | GDSC2 | -3.9527408387067 |
| NCI-H1688 | SCLC | | lung | lung small cell carcinoma | 0.022501 | 0.899432 | GDSC2 | -3.79419552631494 |
| MS-1 | SCLC | | lung | lung small cell carcinoma | 0.022621 | 0.927409 | GDSC2 | -3.78887660060644 |
| NCI-H1876 | SCLC | | lung | lung small cell carcinoma | 0.024503 | 0.895467 | GDSC2 | -3.70895971994813 |
| SW1271 | SCLC | | lung | lung small cell carcinoma | 0.025424 | 0.836223 | GDSC2 | -3.67206166918778 |
| DMS-53 | SCLC | | lung | lung small cell carcinoma | 0.031372 | 0.874532 | GDSC2 | -3.46183950363172 |
| NCI-H196 | SCLC | | lung | lung small cell carcinoma | 0.044117 | 0.894626 | GDSC2 | -3.12091008328222 |
| NCI-H64 | SCLC | | lung | lung small cell carcinoma | 0.045372 | 0.893771 | GDSC2 | -3.09286010428534 |
| COLO-668 | SCLC | | lung | lung small cell carcinoma | 0.046366 | 0.906247 | GDSC2 | -3.07118884696944 |
| NCI-H211 | SCLC | | lung | lung small cell carcinoma | 0.059485 | 0.944123 | GDSC2 | -2.82203109905365 |
| COR-L88 | SCLC | | lung | lung small cell carcinoma | 0.119125 | 0.952209 | GDSC2 | -2.12758191700777 |
| LU-165 | SCLC | | lung | lung small cell carcinoma | 0.166904 | 0.948161 | GDSC2 | -1.79033648215456 |
| NCI-H2066 | SCLC | | lung | lung small cell carcinoma | 0.818439 | 0.968838 | GDSC2 | -0.20035641152119 |
| NCI-H748 | SCLC | | lung | lung small cell carcinoma | 0.003114 | 0.628847 | GDSC1 | -5.77184720557033 |
| SBC-1 | SCLC | | lung | lung small cell carcinoma | 0.003398 | 0.647467 | GDSC1 | -5.6845682557324 |
| HCC-33 | SCLC | | lung | lung small cell carcinoma | 0.004024 | 0.683992 | GDSC1 | -5.5154788461847 |
| IST-SL1 | SCLC | | lung | lung small cell carcinoma | 0.004214 | 0.684432 | GDSC1 | -5.46934296360014 |
| LU-139 | SCLC | | lung | lung small cell carcinoma | 0.004239 | 0.671133 | GDSC1 | -5.46342788661164 |
| NCI-H2196 | SCLC | | lung | lung small cell carcinoma | 0.005701 | 0.723856 | GDSC1 | -5.16711368093269 |
| COR-L95 | SCLC | | lung | lung small cell carcinoma | 0.00697 | 0.746527 | GDSC1 | -4.9661400542097 |

**Supplementary Table 1. Merged GDSC1 and GDSC2 datasets (860 cell lines; duplicates were excluded from GDSC2 datasets)**

| Sample name | TCGA | TCGA organ system | Organ system | Organ system subtype | IC50 | AUC | Dataset | LN_IC50 |
|---|---|---|---|---|---|---|---|---|
| NCI-H2227 | SCLC | | lung | lung small cell carcinoma | 0.008146 | 0.76483 | GDSC1 | -4.81022826975592 |
| LU-134-A | SCLC | | lung | lung small cell carcinoma | 0.00822 | 0.761297 | GDSC1 | -4.80118506991405 |
| NCI-H2081 | SCLC | | lung | lung small cell carcinoma | 0.010145 | 0.797915 | GDSC1 | -4.59077430570436 |
| LB647-SCLC | SCLC | | lung | lung small cell carcinoma | 0.010397 | 0.784661 | GDSC1 | -4.5662379759863 |
| NCI-H510A | SCLC | | lung | lung small cell carcinoma | 0.010478 | 0.802634 | GDSC1 | -4.55847745799611 |
| NCI-H345 | SCLC | | lung | lung small cell carcinoma | 0.01111 | 0.787461 | GDSC1 | -4.4999096753306 |
| NCI-H1304 | SCLC | | lung | lung small cell carcinoma | 0.012201 | 0.815522 | GDSC1 | -4.40623736338894 |
| NCI-H1836 | SCLC | | lung | lung small cell carcinoma | 0.017661 | 0.853761 | GDSC1 | -4.0363964602679 |
| NCI-H1436 | SCLC | | lung | lung small cell carcinoma | 0.024289 | 0.86819 | GDSC1 | -3.71773170602097 |
| NCI-H2171 | SCLC | | lung | lung small cell carcinoma | 0.03458 | 0.950971 | GDSC1 | -3.36447979872699 |
| NCI-H128 | SCLC | | lung | lung small cell carcinoma | 0.054393 | 0.885646 | GDSC1 | -2.91151980987036 |
| NCI-H187 | SCLC | | lung | lung small cell carcinoma | 0.056995 | 0.935444 | GDSC1 | -2.8647917342934 |
| DMS-79 | SCLC | | lung | lung small cell carcinoma | 0.067533 | 0.92992 | GDSC1 | -2.695138911682 |
| A101D | SKCM | Skin Cutaneous Melanoma | skin | melanoma | 0.001033 | 0.561269 | GDSC2 | -6.87528808884464 |
| G-361 | SKCM | Skin Cutaneous Melanoma | skin | melanoma | 0.002282 | 0.662833 | GDSC2 | -6.08270302754225 |
| COLO-792 | SKCM | Skin Cutaneous Melanoma | skin | melanoma | 0.002634 | 0.677107 | GDSC2 | -5.93925167566105 |
| WM35 | SKCM | Skin Cutaneous Melanoma | skin | melanoma | 0.003087 | 0.688772 | GDSC2 | -5.78055553346212 |
| SH-4 | SKCM | Skin Cutaneous Melanoma | skin | melanoma | 0.003195 | 0.695729 | GDSC2 | -5.74616819115264 |
| A2058 | SKCM | Skin Cutaneous Melanoma | skin | melanoma | 0.003245 | 0.723897 | GDSC2 | -5.73063992882608 |
| IGR-1 | SKCM | Skin Cutaneous Melanoma | skin | melanoma | 0.003529 | 0.719598 | GDSC2 | -5.646740734289 |
| WM793B | SKCM | Skin Cutaneous Melanoma | skin | melanoma | 0.003712 | 0.719434 | GDSC2 | -5.59618446405818 |
| GAK | SKCM | Skin Cutaneous Melanoma | skin | melanoma | 0.003922 | 0.73438 | GDSC2 | -5.54115355120798 |
| LOXIMVI | SKCM | Skin Cutaneous Melanoma | skin | melanoma | 0.004036 | 0.733942 | GDSC2 | -5.51250117649077 |
| SK-MEL-2 | SKCM | Skin Cutaneous Melanoma | skin | melanoma | 0.004058 | 0.750169 | GDSC2 | -5.50706503757851 |
| IST-MEL1 | SKCM | Skin Cutaneous Melanoma | skin | melanoma | 0.004265 | 0.709284 | GDSC2 | -5.45731309803849 |
| RPMI-7951 | SKCM | Skin Cutaneous Melanoma | skin | melanoma | 0.004291 | 0.745318 | GDSC2 | -5.45123547297275 |
| COLO-679 | SKCM | Skin Cutaneous Melanoma | skin | melanoma | 0.004528 | 0.728199 | GDSC2 | -5.39747493808126 |
| A375 | SKCM | Skin Cutaneous Melanoma | skin | melanoma | 0.004691 | 0.742611 | GDSC2 | -5.36210949963582 |
| M14 | SKCM | Skin Cutaneous Melanoma | skin | melanoma | 0.004803 | 0.734585 | GDSC2 | -5.33851455629945 |
| HT-144 | SKCM | Skin Cutaneous Melanoma | skin | melanoma | 0.004888 | 0.750928 | GDSC2 | -5.32097205711284 |
| WM278 | SKCM | Skin Cutaneous Melanoma | skin | melanoma | 0.005493 | 0.716211 | GDSC2 | -5.20428072462165 |
| IGR-37 | SKCM | Skin Cutaneous Melanoma | skin | melanoma | 0.006747 | 0.800563 | GDSC2 | -4.99865731733685 |
| HMV-II | SKCM | Skin Cutaneous Melanoma | skin | melanoma | 0.007022 | 0.764257 | GDSC2 | -4.9587072012359 |
| CHL-1 | SKCM | Skin Cutaneous Melanoma | skin | melanoma | 0.007487 | 0.795962 | GDSC2 | -4.89458709573359 |
| UACC-62 | SKCM | Skin Cutaneous Melanoma | skin | melanoma | 0.00845 | 0.801526 | GDSC2 | -4.77358883761305 |
| C32 | SKCM | Skin Cutaneous Melanoma | skin | melanoma | 0.008831 | 0.782086 | GDSC2 | -4.72948702049547 |
| Mewo | SKCM | Skin Cutaneous Melanoma | skin | melanoma | 0.009827 | 0.795362 | GDSC2 | -4.62262157960185 |
| WM-115 | SKCM | Skin Cutaneous Melanoma | skin | melanoma | 0.010391 | 0.803784 | GDSC2 | -4.56681523211163 |
| COLO-829 | SKCM | Skin Cutaneous Melanoma | skin | melanoma | 0.010942 | 0.752753 | GDSC2 | -4.5151466833405 |
| UACC-257 | SKCM | Skin Cutaneous Melanoma | skin | melanoma | 0.013285 | 0.808746 | GDSC2 | -4.32111969977036 |
| WM1552C | SKCM | Skin Cutaneous Melanoma | skin | melanoma | 0.015092 | 0.861327 | GDSC2 | -4.19359047688007 |
| MEL-JUSO | SKCM | Skin Cutaneous Melanoma | skin | melanoma | 0.019277 | 0.856273 | GDSC2 | -3.94884260356668 |
| SK-MEL-24 | SKCM | Skin Cutaneous Melanoma | skin | melanoma | 0.094184 | 0.935276 | GDSC2 | -2.36250496320624 |
| SK-MEL-1 | SKCM | Skin Cutaneous Melanoma | skin | melanoma | 0.225859 | 0.951072 | GDSC2 | -1.48784436821789 |
| MMAc-SF | SKCM | Skin Cutaneous Melanoma | skin | melanoma | 0.000187 | 0.229045 | GDSC1 | -8.58440194110969 |
| MZ2-MEL | SKCM | Skin Cutaneous Melanoma | skin | melanoma | 0.000395 | 0.332713 | GDSC1 | -7.83662479306315 |
| LB2518-MEL | SKCM | Skin Cutaneous Melanoma | skin | melanoma | 0.000526 | 0.36593 | GDSC1 | -7.55020934522656 |
| LB373-MEL-D | SKCM | Skin Cutaneous Melanoma | skin | melanoma | 0.000529 | 0.368464 | GDSC1 | -7.54452212610598 |
| CP66-MEL | SKCM | Skin Cutaneous Melanoma | skin | melanoma | 0.000627 | 0.395458 | GDSC1 | -7.37456401733135 |
| MZ7-mel | SKCM | Skin Cutaneous Melanoma | skin | melanoma | 0.000867 | 0.443331 | GDSC1 | -7.05047158118373 |
| COLO-800 | SKCM | Skin Cutaneous Melanoma | skin | melanoma | 0.014195 | 0.849108 | GDSC1 | -4.2548654890572 |
| Hs-746T | STAD | Stomach adenocarcinoma | digestive_system | stomach | 0.003164 | 0.675413 | GDSC2 | -5.75591822907673 |
| SNU-16 | STAD | Stomach adenocarcinoma | digestive_system | stomach | 0.003331 | 0.72103 | GDSC2 | -5.70448271977059 |
| AGS | STAD | Stomach adenocarcinoma | digestive_system | stomach | 0.003652 | 0.724174 | GDSC2 | -5.61248031624941 |
| HGC-27 | STAD | Stomach adenocarcinoma | digestive_system | stomach | 0.004064 | 0.744089 | GDSC2 | -5.50558756870596 |
| 23132-87 | STAD | Stomach adenocarcinoma | digestive_system | stomach | 0.004728 | 0.732685 | GDSC2 | -5.35425299887834 |
| TGBC11TKB | STAD | Stomach adenocarcinoma | digestive_system | stomach | 0.005923 | 0.771384 | GDSC2 | -5.12891220168731 |
| GCIY | STAD | Stomach adenocarcinoma | digestive_system | stomach | 0.007435 | 0.768011 | GDSC2 | -4.90155669907002 |
| SNU-5 | STAD | Stomach adenocarcinoma | digestive_system | stomach | 0.007599 | 0.790707 | GDSC2 | -4.87973861929449 |
| RERF-GC-1B | STAD | Stomach adenocarcinoma | digestive_system | stomach | 0.007774 | 0.786869 | GDSC2 | -4.85697044655211 |
| MKN45 | STAD | Stomach adenocarcinoma | digestive_system | stomach | 0.008737 | 0.784474 | GDSC2 | -4.74018839766618 |
| MKN7 | STAD | Stomach adenocarcinoma | digestive_system | stomach | 0.009623 | 0.745157 | GDSC2 | -4.64359921260787 |
| SK-GT-2 | STAD | Stomach adenocarcinoma | digestive_system | stomach | 0.009628 | 0.820152 | GDSC2 | -4.64307975906131 |
| NUGC-3 | STAD | Stomach adenocarcinoma | digestive_system | stomach | 0.01115 | 0.804671 | GDSC2 | -4.49631578107601 |
| IM-95 | STAD | Stomach adenocarcinoma | digestive_system | stomach | 0.013188 | 0.835926 | GDSC2 | -4.32844795377265 |
| FU97 | STAD | Stomach adenocarcinoma | digestive_system | stomach | 0.013231 | 0.80497 | GDSC2 | -4.32519271792186 |
| MKN28 | STAD | Stomach adenocarcinoma | digestive_system | stomach | 0.015256 | 0.827937 | GDSC2 | -4.18278241068402 |
| RF-48 | STAD | Stomach adenocarcinoma | digestive_system | stomach | 0.016143 | 0.875187 | GDSC2 | -4.12626875980675 |
| SNU-1 | STAD | Stomach adenocarcinoma | digestive_system | stomach | 0.018534 | 0.884545 | GDSC2 | -3.98814839581764 |
| NCI-N87 | STAD | Stomach adenocarcinoma | digestive_system | stomach | 0.022723 | 0.823723 | GDSC2 | -3.78437765159248 |
| KATOIII | STAD | Stomach adenocarcinoma | digestive_system | stomach | 0.025049 | 0.835805 | GDSC2 | -3.68692137240777 |
| NUGC-4 | STAD | Stomach adenocarcinoma | digestive_system | stomach | 0.026643 | 0.820873 | GDSC2 | -3.62522882703766 |
| OCUM-1 | STAD | Stomach adenocarcinoma | digestive_system | stomach | 0.183433 | 0.936488 | GDSC2 | -1.69590580077681 |
| HSC-39 | STAD | Stomach adenocarcinoma | digestive_system | stomach | 0.003227 | 0.660936 | GDSC1 | -5.73620236591231 |
| HTC-C3 | THCA | Thyroid carcinoma | thyroid | thyroid | 0.002225 | 0.644809 | GDSC2 | -6.10799836336393 |
| ASH-3 | THCA | Thyroid carcinoma | thyroid | thyroid | 0.004164 | 0.735628 | GDSC2 | -5.48127912822941 |
| 8305C | THCA | Thyroid carcinoma | thyroid | thyroid | 0.004247 | 0.707537 | GDSC2 | -5.461542427651 |
| BHT-101 | THCA | Thyroid carcinoma | thyroid | thyroid | 0.004437 | 0.740247 | GDSC2 | -5.41777680658536 |
| 8505C | THCA | Thyroid carcinoma | thyroid | thyroid | 0.005688 | 0.732629 | GDSC2 | -5.16939658864812 |
| B-CPAP | THCA | Thyroid carcinoma | thyroid | thyroid | 0.005922 | 0.741308 | GDSC2 | -5.12908104930274 |
| FTC-133 | THCA | Thyroid carcinoma | thyroid | thyroid | 0.006277 | 0.751147 | GDSC2 | -5.07086311964674 |
| TT | THCA | Thyroid carcinoma | thyroid | thyroid | 0.00687 | 0.716028 | GDSC2 | -4.98059117274788 |
| CAL-62 | THCA | Thyroid carcinoma | thyroid | thyroid | 0.008694 | 0.800541 | GDSC2 | -4.74512214641554 |
| K5 | THCA | Thyroid carcinoma | thyroid | thyroid | 0.00922 | 0.814232 | GDSC2 | -4.68638024141363 |
| IHH-4 | THCA | Thyroid carcinoma | thyroid | thyroid | 0.010991 | 0.83809 | GDSC2 | -4.51067852289537 |
| CGTH-W-1 | THCA | Thyroid carcinoma | thyroid | thyroid | 0.015386 | 0.84679 | GDSC2 | -4.17429727394539 |
| TT2609-C02 | THCA | Thyroid carcinoma | thyroid | thyroid | 0.103646 | 0.886075 | GDSC2 | -2.26677403225843 |
| RO82-W-1 | THCA | Thyroid carcinoma | thyroid | thyroid | 0.145274 | 0.879874 | GDSC2 | -1.92913366454148 |
| MFE-319 | UCEC | Uterine Corpus Endometrial Carci | urogenital_system | endometrium | 0.002976 | 0.692365 | GDSC2 | -5.81717516201129 |
| SNG-M | UCEC | Uterine Corpus Endometrial Carci | urogenital_system | endometrium | 0.005894 | 0.790865 | GDSC2 | -5.13382039466663 |
| HEC-1 | UCEC | Uterine Corpus Endometrial Carci | urogenital_system | endometrium | 0.008015 | 0.778811 | GDSC2 | -4.82644049292062 |
| KLE | UCEC | Uterine Corpus Endometrial Carci | urogenital_system | endometrium | 0.009001 | 0.75049 | GDSC2 | -4.71041959670719 |
| MFE-280 | UCEC | Uterine Corpus Endometrial Carci | urogenital_system | endometrium | 0.010921 | 0.792485 | GDSC2 | -4.51706773776655 |
| EN | UCEC | Uterine Corpus Endometrial Carci | urogenital_system | endometrium | 0.011069 | 0.800076 | GDSC2 | -4.50360687057865 |
| AN3-CA | UCEC | Uterine Corpus Endometrial Carci | urogenital_system | endometrium | 0.011945 | 0.836352 | GDSC2 | -4.42744249820436 |
| COLO-684 | UCEC | Uterine Corpus Endometrial Carci | urogenital_system | endometrium | 0.023545 | 0.868629 | GDSC2 | -3.74884179528662 |
| MFE-296 | UCEC | Uterine Corpus Endometrial Carci | urogenital_system | endometrium | 0.03565 | 0.890578 | GDSC2 | -3.33400613212183 |
| A253 | UNCLASSIFIED | | aero_digestive_tract | head and neck | 0.006114 | 0.78755 | GDSC2 | -5.09717405551349 |
| NCI-H3118 | UNCLASSIFIED | | aero_digestive_tract | head and neck | 0.007701 | 0.787097 | GDSC2 | -4.86640508842502 |
| DEL | UNCLASSIFIED | | blood | lymphoid neoplasm other | 0.001282 | 0.599824 | GDSC2 | -6.65933392048366 |

**Supplementary Table 1. Merged GDSC1 and GDSC2 datasets (860 cell lines; duplicates were excluded from GDSC2 datasets)**

| Sample name | TCGA | TCGA organ system | Organ system | Organ system subtype | IC50 | AUC | Dataset | LN_IC50 |
|---|---|---|---|---|---|---|---|---|
| U-698-M | UNCLASSIFIED | | blood | B cell leukemia | 0.001585 | 0.625516 | GDSC2 | -6.44717087165289 |
| WSU-NHL | UNCLASSIFIED | | blood | lymphoid neoplasm other | 0.001999 | 0.660209 | GDSC2 | -6.21510822346387 |
| GA-10 | UNCLASSIFIED | | blood | Burkitt lymphoma | 0.002632 | 0.693456 | GDSC2 | -5.94001126551907 |
| H9 | UNCLASSIFIED | | blood | lymphoid neoplasm other | 0.002644 | 0.696654 | GDSC2 | -5.9354623569927 |
| BL-41 | UNCLASSIFIED | | blood | Burkitt lymphoma | 0.003074 | 0.728739 | GDSC2 | -5.78477563386573 |
| SUP-HD1 | UNCLASSIFIED | | blood | Hodgkin lymphoma | 0.003116 | 0.690403 | GDSC2 | -5.77120515097364 |
| SUP-M2 | UNCLASSIFIED | | blood | anaplastic large cell lymphoma | 0.003128 | 0.729593 | GDSC2 | -5.76736145629907 |
| MHH-CALL-2 | UNCLASSIFIED | | blood | B cell leukemia | 0.003138 | 0.716228 | GDSC2 | -5.7641696246713 |
| KOPN-8 | UNCLASSIFIED | | blood | B cell leukemia | 0.003151 | 0.72275 | GDSC2 | -5.760035416207 |
| EB-3 | UNCLASSIFIED | | blood | Burkitt lymphoma | 0.003345 | 0.708777 | GDSC2 | -5.70028858540195 |
| MV-4-11 | UNCLASSIFIED | | blood | leukemia | 0.003374 | 0.736905 | GDSC2 | -5.69165629485836 |
| JiyoyeP-2003 | UNCLASSIFIED | | blood | Burkitt lymphoma | 0.003433 | 0.719783 | GDSC2 | -5.67432076450658 |
| NAMALWA | UNCLASSIFIED | | blood | Burkitt lymphoma | 0.003459 | 0.729648 | GDSC2 | -5.66677574902711 |
| L-540 | UNCLASSIFIED | | blood | Hodgkin lymphoma | 0.003518 | 0.733961 | GDSC2 | -5.64986263266677 |
| RPMI-6666 | UNCLASSIFIED | | blood | Hodgkin lymphoma | 0.003738 | 0.731047 | GDSC2 | -5.58920456994877 |
| BALL-1 | UNCLASSIFIED | | blood | B cell leukemia | 0.003816 | 0.744675 | GDSC2 | -5.5685525253961 |
| L-1236 | UNCLASSIFIED | | blood | Hodgkin lymphoma | 0.003868 | 0.740755 | GDSC2 | -5.55501770139109 |
| KARPAS-299 | UNCLASSIFIED | | blood | anaplastic large cell lymphoma | 0.003904 | 0.743342 | GDSC2 | -5.54575361043129 |
| SR | UNCLASSIFIED | | blood | lymphoid neoplasm other | 0.003936 | 0.752982 | GDSC2 | -5.53759029979213 |
| ST486 | UNCLASSIFIED | | blood | Burkitt lymphoma | 0.003984 | 0.748886 | GDSC2 | -5.52546893925979 |
| DG-75 | UNCLASSIFIED | | blood | Burkitt lymphoma | 0.004017 | 0.729638 | GDSC2 | -5.51721992360499 |
| MLMA | UNCLASSIFIED | | blood | hairy cell leukaemia | 0.004058 | 0.746918 | GDSC2 | -5.50706503757851 |
| KM-H2 | UNCLASSIFIED | | blood | Hodgkin lymphoma | 0.004149 | 0.74429 | GDSC2 | -5.48488793763141 |
| Raji | UNCLASSIFIED | | blood | Burkitt lymphoma | 0.004163 | 0.75534 | GDSC2 | -5.4815193107693 |
| EB2 | UNCLASSIFIED | | blood | Burkitt lymphoma | 0.004254 | 0.732446 | GDSC2 | -5.45989556220409 |
| 697 | UNCLASSIFIED | | blood | lymphoblastic leukemia | 0.004374 | 0.765793 | GDSC2 | -5.43207735672756 |
| WIL2-NS | UNCLASSIFIED | | blood | lymphoid neoplasm other | 0.004381 | 0.760967 | GDSC2 | -5.43047827015038 |
| NALM-6 | UNCLASSIFIED | | blood | B cell leukemia | 0.004601 | 0.769373 | GDSC2 | -5.38148160780881 |
| Hs-445 | UNCLASSIFIED | | blood | Hodgkin lymphoma | 0.004647 | 0.756399 | GDSC2 | -5.3715334288793 |
| EoL-1-cell | UNCLASSIFIED | | blood | haematopoietic neoplasm other | 0.004686 | 0.776186 | GDSC2 | -5.36317593889653 |
| L-428 | UNCLASSIFIED | | blood | Hodgkin lymphoma | 0.004879 | 0.768595 | GDSC2 | -5.32281499814844 |
| P32-ISH | UNCLASSIFIED | | blood | Burkitt lymphoma | 0.004918 | 0.788696 | GDSC2 | -5.31485333518814 |
| HDLM-2 | UNCLASSIFIED | | blood | Hodgkin lymphoma | 0.00497 | 0.759563 | GDSC2 | -5.3043354388736 |
| Mo-T | UNCLASSIFIED | | blood | hairy cell leukaemia | 0.005234 | 0.76428 | GDSC2 | -5.25257947487418 |
| Daudi | UNCLASSIFIED | | blood | Burkitt lymphoma | 0.005355 | 0.793178 | GDSC2 | -5.22972457508243 |
| HC-1 | UNCLASSIFIED | | blood | hairy cell leukaemia | 0.005804 | 0.801283 | GDSC2 | -5.1492079439602 |
| CA46 | UNCLASSIFIED | | blood | Burkitt lymphoma | 0.006094 | 0.771389 | GDSC2 | -5.10045059841862 |
| MHH-PREB-1 | UNCLASSIFIED | | blood | B cell leukemia | 0.009462 | 0.84334 | GDSC2 | -4.66047150177318 |
| MN-60 | UNCLASSIFIED | | blood | B cell leukemia | 0.009838 | 0.808624 | GDSC2 | -4.62150284060899 |
| Ramos-2G6-4C10 | UNCLASSIFIED | | blood | Burkitt lymphoma | 0.010749 | 0.89708 | GDSC2 | -4.53294255199121 |
| KARPAS-231 | UNCLASSIFIED | | blood | B cell leukemia | 0.011299 | 0.900235 | GDSC2 | -4.48304105275503 |
| REH | UNCLASSIFIED | | blood | B cell leukemia | 0.011539 | 0.878932 | GDSC2 | -4.46202267676961 |
| HH | UNCLASSIFIED | | blood | T cell leukemia | 0.018446 | 0.868358 | GDSC2 | -3.99290773416861 |
| HD-MY-Z | UNCLASSIFIED | | blood | Hodgkin lymphoma | 0.163965 | 0.884983 | GDSC2 | -1.80810228856823 |
| NK-92MI | UNCLASSIFIED | | blood | lymphoid neoplasm other | 0.000761 | 0.417139 | GDSC1 | -7.18087720010259 |
| SU-DHL-1 | UNCLASSIFIED | | blood | anaplastic large cell lymphoma | 0.003431 | 0.675764 | GDSC1 | -5.67490351510582 |
| ROS-50 | UNCLASSIFIED | | blood | B cell leukemia | 0.007336 | 0.740226 | GDSC1 | -4.91496154402797 |
| SKM-1 | UNCLASSIFIED | | blood | haematopoietic neoplasm other | 0.054058 | 0.926092 | GDSC1 | -2.91769773474865 |
| ES6 | UNCLASSIFIED | | bone | ewings sarcoma | 0.001847 | 0.620566 | GDSC2 | -6.29419257776503 |
| MG-63 | UNCLASSIFIED | | bone | osteosarcoma | 0.002852 | 0.67764 | GDSC2 | -5.85973477643009 |
| CS1 | UNCLASSIFIED | | bone | bone other | 0.002907 | 0.667556 | GDSC2 | -5.8406336574054 |
| CAL-78 | UNCLASSIFIED | | bone | chondrosarcoma | 0.003759 | 0.727947 | GDSC2 | -5.5836023144001 |
| CAL-72 | UNCLASSIFIED | | bone | osteosarcoma | 0.004653 | 0.732531 | GDSC2 | -5.37024310611963 |
| HOS | UNCLASSIFIED | | bone | osteosarcoma | 0.004683 | 0.764469 | GDSC2 | -5.36381634878073 |
| EW-1 | UNCLASSIFIED | | bone | ewings sarcoma | 0.005128 | 0.769145 | GDSC2 | -5.27303955936377 |
| ES8 | UNCLASSIFIED | | bone | ewings sarcoma | 0.005149 | 0.778948 | GDSC2 | -5.26895275791813 |
| EW-22 | UNCLASSIFIED | | bone | ewings sarcoma | 0.005333 | 0.765251 | GDSC2 | -5.23384134736367 |
| ES7 | UNCLASSIFIED | | bone | ewings sarcoma | 0.005855 | 0.783278 | GDSC2 | -5.14045928193246 |
| CHSA8926 | UNCLASSIFIED | | bone | chondrosarcoma | 0.005919 | 0.762576 | GDSC2 | -5.12958776327355 |
| SK-PN-DW | UNCLASSIFIED | | bone | ewings sarcoma | 0.006244 | 0.797517 | GDSC2 | -5.07613427632895 |
| ES1 | UNCLASSIFIED | | bone | ewings sarcoma | 0.00675 | 0.791477 | GDSC2 | -4.9982127740977 |
| SJSA-1 | UNCLASSIFIED | | bone | bone other | 0.006855 | 0.784854 | GDSC2 | -4.98277696596786 |
| HuO9 | UNCLASSIFIED | | bone | osteosarcoma | 0.00698 | 0.794534 | GDSC2 | -4.96470636220786 |
| SK-ES-1 | UNCLASSIFIED | | bone | ewings sarcoma | 0.007273 | 0.804599 | GDSC2 | -4.92358641780973 |
| EW-7 | UNCLASSIFIED | | bone | ewings sarcoma | 0.007388 | 0.809441 | GDSC2 | -4.90789821664514 |
| EW-18 | UNCLASSIFIED | | bone | ewings sarcoma | 0.008293 | 0.820037 | GDSC2 | -4.79234349351307 |
| CADO-ES1 | UNCLASSIFIED | | bone | ewings sarcoma | 0.008981 | 0.835745 | GDSC2 | -4.71264404429332 |
| TC-71 | UNCLASSIFIED | | bone | ewings sarcoma | 0.009853 | 0.850061 | GDSC2 | -4.6199793016418 |
| EW-24 | UNCLASSIFIED | | bone | ewings sarcoma | 0.009877 | 0.788986 | GDSC2 | -4.61754645705615 |
| EW-11 | UNCLASSIFIED | | bone | ewings sarcoma | 0.010173 | 0.818149 | GDSC2 | -4.58801812717053 |
| EW-3 | UNCLASSIFIED | | bone | ewings sarcoma | 0.010256 | 0.820904 | GDSC2 | -4.57989237880382 |
| G-292-Clone-A141B1 | UNCLASSIFIED | | bone | osteosarcoma | 0.011401 | 0.792458 | GDSC2 | -4.47405420813055 |
| ES4 | UNCLASSIFIED | | bone | ewings sarcoma | 0.013603 | 0.814758 | GDSC2 | -4.29746492233084 |
| EW-16 | UNCLASSIFIED | | bone | ewings sarcoma | 0.01841 | 0.85878 | GDSC2 | -3.99486128373715 |
| H-EMC-SS | UNCLASSIFIED | | bone | chondrosarcoma | 0.018842 | 0.822159 | GDSC2 | -3.97166685835566 |
| NOS-1 | UNCLASSIFIED | | bone | osteosarcoma | 0.01923 | 0.887431 | GDSC2 | -3.95128371938145 |
| U-2-OS | UNCLASSIFIED | | bone | osteosarcoma | 0.01933 | 0.850856 | GDSC2 | -3.9460969857619 |
| NY | UNCLASSIFIED | | bone | osteosarcoma | 0.024989 | 0.89802 | GDSC2 | -3.68931955094234 |
| HuO-3N1 | UNCLASSIFIED | | bone | osteosarcoma | 0.028267 | 0.865322 | GDSC2 | -3.56606023246909 |
| MHH-ES-1 | UNCLASSIFIED | | bone | ewings sarcoma | 0.028767 | 0.897921 | GDSC2 | -3.54852638214248 |
| EW-13 | UNCLASSIFIED | | bone | ewings sarcoma | 0.052193 | 0.918119 | GDSC2 | -2.95280689270256 |
| Saos-2 | UNCLASSIFIED | | bone | osteosarcoma | 0.27205 | 0.971932 | GDSC2 | -1.3017694060502 |
| ES5 | UNCLASSIFIED | | bone | ewings sarcoma | 0.012617 | 0.805581 | GDSC1 | -4.37271016803653 |
| ES3 | UNCLASSIFIED | | bone | ewings sarcoma | 0.020736 | 0.878212 | GDSC1 | -3.87588395881227 |
| EW-12 | UNCLASSIFIED | | bone | ewings sarcoma | 0.069303 | 0.881217 | GDSC1 | -2.66926708367998 |
| ZR-75-30 | BRCA | Breast invasive carcinoma | breast | breast | 0.209801 | 0.931953 | GDSC2 | -1.56159581658707 |
| ECC10 | UNCLASSIFIED | | digestive_system | stomach | 0.004022 | 0.741769 | GDSC2 | -5.51597598763168 |
| MKN1 | UNCLASSIFIED | | digestive_system | stomach | 0.006297 | 0.743262 | GDSC2 | -5.06768194947553 |
| HuCCT1 | UNCLASSIFIED | | digestive_system | biliary tract | 0.007323 | 0.772962 | GDSC2 | -4.9167351989032 |
| ETK-1 | UNCLASSIFIED | | digestive_system | biliary tract | 0.007456 | 0.775614 | GDSC2 | -4.89873620159885 |
| TGBC1TKB | UNCLASSIFIED | | digestive_system | biliary tract | 0.010325 | 0.781535 | GDSC2 | -4.57318714013504 |
| EGI-1 | UNCLASSIFIED | | digestive_system | biliary tract | 0.014366 | 0.786699 | GDSC2 | -4.24289097532953 |
| TGBC24TKB | UNCLASSIFIED | | digestive_system | biliary tract | 0.10926 | 0.932226 | GDSC2 | -2.21402491601457 |
| HuTu-80 | UNCLASSIFIED | | digestive_system | digestive system other | 0.001119 | 0.480839 | GDSC1 | -6.79531984965235 |
| ECC12 | UNCLASSIFIED | | digestive_system | stomach | 0.001495 | 0.525732 | GDSC1 | -6.50562907213949 |
| SCH | UNCLASSIFIED | | digestive_system | stomach | 0.085445 | 0.958391 | GDSC1 | -2.45988238488327 |
| G-401 | UNCLASSIFIED | | kidney | kidney | 0.004491 | 0.721805 | GDSC2 | -5.40567988487654 |
| COR-L23 | UNCLASSIFIED | | lung | lung NSCLC large cell | 0.00418 | 0.704014 | GDSC2 | -5.47744403244547 |
| NCI-H1703 | UNCLASSIFIED | | lung | lung NSCLC adenocarcinoma | 0.004233 | 0.722618 | GDSC2 | -5.46484431744335 |
| NCI-H1915 | UNCLASSIFIED | | lung | lung NSCLC large cell | 0.007882 | 0.758953 | GDSC2 | -4.84317360020933 |

**Supplementary Table 1. Merged GDSC1 and GDSC2 datasets (860 cell lines; duplicates were excluded from GDSC2 datasets)**

| Sample name | TCGA | TCGA organ system | Organ system | Organ system subtype | IC50 | AUC | Dataset | LN_IC50 |
|---|---|---|---|---|---|---|---|---|
| LCLC-103H | UNCLASSIFIED | | lung | lung NSCLC large cell | 0.007981 | 0.792115 | GDSC2 | -4.83069156208827 |
| LCLC-97TM1 | UNCLASSIFIED | | lung | lung NSCLC large cell | 0.008206 | 0.8063 | GDSC2 | -4.80288968496214 |
| NCI-H1155 | UNCLASSIFIED | | lung | lung NSCLC large cell | 0.009224 | 0.814293 | GDSC2 | -4.68594649601538 |
| NCI-H720 | UNCLASSIFIED | | lung | lung NSCLC carcinoid | 0.009687 | 0.81714 | GDSC2 | -4.63697049853789 |
| NCI-H1299 | UNCLASSIFIED | | lung | lung NSCLC large cell | 0.010588 | 0.813158 | GDSC2 | -4.54803399461728 |
| HOP-92 | UNCLASSIFIED | | lung | lung NSCLC large cell | 0.010746 | 0.78612 | GDSC2 | -4.53322168667551 |
| NCI-H596 | UNCLASSIFIED | | lung | lung NSCLC adenocarcinoma | 0.011825 | 0.813994 | GDSC2 | -4.43753934460414 |
| NCI-H1581 | UNCLASSIFIED | | lung | lung NSCLC large cell | 0.012245 | 0.847768 | GDSC2 | -4.40263758857801 |
| NCI-H810 | UNCLASSIFIED | | lung | lung NSCLC large cell | 0.013092 | 0.854989 | GDSC2 | -4.3357539223432 |
| UMC-11 | UNCLASSIFIED | | lung | lung NSCLC carcinoid | 0.018502 | 0.858787 | GDSC2 | -3.98987644463301 |
| IA-LM | UNCLASSIFIED | | lung | lung NSCLC large cell | 0.036362 | 0.905629 | GDSC2 | -3.31423100568506 |
| NCI-H661 | UNCLASSIFIED | | lung | lung NSCLC large cell | 0.036746 | 0.884754 | GDSC2 | -3.30372590278459 |
| LU-65 | UNCLASSIFIED | | lung | lung NSCLC large cell | 0.048229 | 0.900949 | GDSC2 | -3.03179477909921 |
| NCI-H835 | UNCLASSIFIED | | lung | lung NSCLC carcinoid | 0.060368 | 0.949365 | GDSC2 | -2.80729611576018 |
| NCI-H727 | UNCLASSIFIED | | lung | lung NSCLC carcinoid | 0.063839 | 0.907188 | GDSC2 | -2.75139099012367 |
| LU-99A | UNCLASSIFIED | | lung | lung NSCLC large cell | 0.28162 | 0.929303 | GDSC2 | -1.26719663449297 |
| NCI-H2135 | UNCLASSIFIED | | lung | lung NSCLC not specified | 0.004486 | 0.698685 | GDSC1 | -5.40679384288412 |
| NCI-H1770 | UNCLASSIFIED | | lung | lung NSCLC not specified | 0.085353 | 0.965078 | GDSC1 | -2.46095968097453 |
| KP-3 | UNCLASSIFIED | | pancreas | pancreas | 0.011373 | 0.770659 | GDSC2 | -4.47651315377983 |
| A388 | UNCLASSIFIED | | skin | skin other | 0.000387 | 0.332426 | GDSC1 | -7.85708586493449 |
| DJM-1 | UNCLASSIFIED | | skin | skin other | 0.002517 | 0.610956 | GDSC1 | -5.98468756282896 |
| SW872 | UNCLASSIFIED | | soft_tissue | soft tissue other | 0.002724 | 0.659125 | GDSC2 | -5.90565389069487 |
| RH-1 | UNCLASSIFIED | | soft_tissue | rhabdomyosarcoma | 0.002936 | 0.711764 | GDSC2 | -5.83070716822987 |
| HT-1080 | UNCLASSIFIED | | soft_tissue | fibrosarcoma | 0.004316 | 0.719323 | GDSC2 | -5.44542623158625 |
| G-402 | UNCLASSIFIED | | soft_tissue | soft tissue other | 0.00457 | 0.73664 | GDSC2 | -5.38824207407602 |
| Hs-633T | UNCLASSIFIED | | soft_tissue | fibrosarcoma | 0.004578 | 0.766056 | GDSC2 | -5.38649305745176 |
| A673 | UNCLASSIFIED | | soft_tissue | rhabdomyosarcoma | 0.005203 | 0.772067 | GDSC2 | -5.25851989667397 |
| RH-41 | UNCLASSIFIED | | soft_tissue | rhabdomyosarcoma | 0.006642 | 0.79734 | GDSC2 | -5.01434215602758 |
| SK-UT-1 | UNCLASSIFIED | | soft_tissue | soft tissue other | 0.006704 | 0.785021 | GDSC2 | -5.00505091580236 |
| SW982 | UNCLASSIFIED | | soft_tissue | soft tissue other | 0.006784 | 0.782365 | GDSC2 | -4.99318838049254 |
| A204 | UNCLASSIFIED | | soft_tissue | rhabdomyosarcoma | 0.007878 | 0.762333 | GDSC2 | -4.84368121443342 |
| MFH-ino | UNCLASSIFIED | | soft_tissue | soft tissue other | 0.008142 | 0.765096 | GDSC2 | -4.81071942890135 |
| KYM-1 | UNCLASSIFIED | | soft_tissue | rhabdomyosarcoma | 0.008224 | 0.830315 | GDSC2 | -4.80069857026933 |
| VA-ES-BJ | UNCLASSIFIED | | soft_tissue | soft tissue other | 0.009224 | 0.777619 | GDSC2 | -4.68594649601538 |
| SK-LMS-1 | UNCLASSIFIED | | soft_tissue | soft tissue other | 0.014803 | 0.853027 | GDSC2 | -4.21292541605078 |
| RD | UNCLASSIFIED | | soft_tissue | rhabdomyosarcoma | 0.017445 | 0.85265 | GDSC2 | -4.0487022043434 |
| SJRH30 | UNCLASSIFIED | | soft_tissue | rhabdomyosarcoma | 0.02061 | 0.898599 | GDSC2 | -3.88197888407977 |
| SW684 | UNCLASSIFIED | | soft_tissue | fibrosarcoma | 0.025875 | 0.838675 | GDSC2 | -3.6544780273966 |
| GCT | UNCLASSIFIED | | soft_tissue | soft tissue other | 0.035406 | 0.892113 | GDSC2 | -3.34087398167905 |
| TE-441-T | UNCLASSIFIED | | soft_tissue | rhabdomyosarcoma | 0.034015 | 0.922575 | GDSC1 | -3.38095367518511 |
| MES-SA | UNCLASSIFIED | | urogenital_system | uterus | 0.003808 | 0.721288 | GDSC2 | -5.57065116205302 |
| NEC8 | UNCLASSIFIED | | urogenital_system | testis | 0.003847 | 0.755426 | GDSC2 | -5.56046165521198 |
| NCCIT | UNCLASSIFIED | | urogenital_system | testis | 0.004751 | 0.760979 | GDSC2 | -5.34940015677735 |
| NTERA-2-cl-D1 | UNCLASSIFIED | | urogenital_system | testis | 0.004828 | 0.74923 | GDSC2 | -5.33332297574685 |
| TYK-nu | UNCLASSIFIED | | urogenital_system | ovary | 0.005037 | 0.715084 | GDSC2 | -5.29094461221862 |
| ESS-1 | UNCLASSIFIED | | urogenital_system | endometrium | 0.005341 | 0.775453 | GDSC2 | -5.2323423776245 |
| A2780 | UNCLASSIFIED | | urogenital_system | ovary | 0.005555 | 0.752626 | GDSC2 | -5.19305685589054 |
| PA-1 | UNCLASSIFIED | | urogenital_system | ovary | 0.006307 | 0.791246 | GDSC2 | -5.06609515130062 |
| ES-2 | UNCLASSIFIED | | urogenital_system | ovary | 0.007199 | 0.768005 | GDSC2 | -4.93381315149497 |
| TC-YIK | UNCLASSIFIED | | urogenital_system | cervix | 0.007444 | 0.802732 | GDSC2 | -4.90034694020213 |
| KGN | UNCLASSIFIED | | urogenital_system | ovary | 0.008727 | 0.800425 | GDSC2 | -4.74133361080096 |
| SKN | UNCLASSIFIED | | urogenital_system | uterus | 0.008896 | 0.791291 | GDSC2 | -4.72215354147391 |
| RMG-I | UNCLASSIFIED | | urogenital_system | ovary | 0.010706 | 0.762784 | GDSC2 | -4.53695094701095 |
| BPH-1 | UNCLASSIFIED | | urogenital_system | prostate | 0.011156 | 0.804018 | GDSC2 | -4.49577780921689 |
| RL95-2 | UNCLASSIFIED | | urogenital_system | endometrium | 0.014075 | 0.850142 | GDSC2 | -4.26335510495638 |
| KURAMOCHI | UNCLASSIFIED | | urogenital_system | ovary | 0.029917 | 0.837322 | GDSC2 | -3.50932839828265 |
| RKN | UNCLASSIFIED | | urogenital_system | ovary | 0.08518 | 0.916179 | GDSC2 | -2.46298861448707 |
| SW954 | UNCLASSIFIED | | urogenital_system | urogenital system other | 0.001971 | 0.573503 | GDSC1 | -6.22921425081155 |
| SW962 | UNCLASSIFIED | | urogenital_system | urogenital system other | 0.004521 | 0.686068 | GDSC1 | -5.39902207066967 |
| JAR | UNCLASSIFIED | | urogenital_system | urogenital system other | 0.012693 | 0.838099 | GDSC1 | -4.3667046185761 |
