# Supplementary material for: Multi-omics analysis identifies repurposing bortezomib in the treatment of kidney-, nervous system-, and hematological cancers
Source: Sci Rep. 2024 Aug 10;14:18576. doi: 10.1038/s41598-024-62339-x (PMC11316778; doi:10.1038/s41598-024-62339-x)
Supplement: Supplementary file 4 — Supplementary Table 3. [file 41598_2024_62339_MOESM4_ESM.pdf]

Supplementary Table 3: Statistical analysis of the 50 most mutated genes identified in sensitive cell line group and comparison across sensitivity groups

1= Mutated gene X axis represent the 50 most mutated genes in sensitive cell lines (49 cell lines)
0= No mutated gene Y axis represent all cell lines in Sanger COSMIC dataset (Complete mutation data)

Insensitive cell lines

Sample n	ADGRV1	ANKRD36	ANKRD36	CSMD1	CSMD3	CUBN	DNAH11	DST	HECTD4	HLA-B	HMCN1	HYDIN	KIAA1671	KIR2DL1	LRP1B	LRP2	MACF1	MGAM	MUC16	MUC4	MUC5B	NBEA	NBPF1	NEB	OBSCN	PCDHA1	PCDHA2	PCDHA3	PCDHA4	PCDHA5	PCDHGA1	PCDHGA2	PCDHGA3	PCDHGA4	PCDHGA5	PCDHGA6	PCDHGB1	PCDHGB2	PCDHGB3	PCLO	PKHD1	PKHD1L1	PLEKHB2	RYR2	RYR3	SYNE1	TP53	TSPEAR	TTN	USH2A
AsPC-1	1	1	1	1	0	0	0	0	0	1	1	1	0	0	1	1	1	0	0	1	0	1	1	1	0	1	1	1	1	1	0	0	0	0	0	0	0	0	0	1	0	0	0	1	1	0	1	0	1	1
BT-474	1	0	1	1	1	0	1	0	0	0	1	1	1	0	0	1	1	1	1	1	0	1	0	1	1	1	1	1	1	1	1	1	1	1	1	1	1	1	1	0	0	0	0	1	1	1	1	1	1	1
COLO-680	1	1	1	0	1	0	1	1	1	0	1	1	1	0	1	0	0	1	1	1	1	1	1	1	1	0	0	0	0	0	1	1	1	1	1	1	1	1	1	0	0	1	1	0	1	1	1	0	1	1
COR-L88	1	1	1	1	1	1	1	0	1	1	1	1	0	0	1	1	0	0	0	1	0	0	0	1	1	0	0	0	0	0	0	0	0	0	0	0	0	0	0	0	1	0	1	1	1	1	1	0	1	1
DMS-79	0	1	1	1	1	1	0	1	1	0	1	1	0	1	1	1	1	1	1	0	0	0	1	0	1	0	0	0	0	0	0	0	0	0	0	0	0	0	0	1	1	0	1	1	1	1	0	0	1	1
EW-13	0	0	1	0	0	0	0	1	0	1	0	1	1	0	0	1	0	1	0	1	0	1	1	1	0	0	0	0	0	0	1	1	1	1	1	1	1	1	1	0	1	0	0	1	0	0	1	1	0	1
JHH-6	1	1	1	0	0	1	1	1	0	1	0	1	1	1	1	0	1	1	0	1	0	0	0	0	0	1	1	1	1	1	1	1	1	0	0	0	1	0	0	1	0	1	1	0	0	1	1	1	0	0
LU-165	0	0	1	0	1	1	1	1	0	1	1	1	0	0	0	1	0	0	1	1	1	0	1	1	1	1	1	1	1	1	0	0	0	0	0	0	0	0	0	0	0	1	1	1	1	1	1	1	1	1
LU-99A	1	1	1	1	1	0	0	0	0	1	1	1	0	0	1	0	0	0	1	1	0	1	1	1	1	1	1	1	1	1	1	1	1	1	1	1	1	1	1	0	1	0	1	1	0	1	0	1	1	1
NCI-H143	1	0	1	1	1	1	1	1	1	0	1	1	1	0	1	1	1	1	1	1	1	0	0	1	1	1	1	1	1	1	1	1	1	1	1	1	1	1	1	0	1	1	1	1	1	1	1	1	1	1
NCI-H156	0	1	1	0	1	1	1	0	1	0	0	1	1	0	1	1	1	1	1	1	1	0	0	1	1	1	1	1	1	1	1	1	0	0	0	0	0	0	0	1	1	1	1	1	1	1	0	1	1	1
NCI-H165	1	1	1	1	1	0	0	1	1	0	1	1	0	0	0	1	1	1	1	1	1	1	1	1	1	1	1	1	1	1	1	1	1	1	0	0	1	0	0	1	0	1	1	1	1	1	1	1	1	1
NCI-H177	1	1	1	1	1	1	1	1	0	0	1	1	0	0	1	1	1	1	1	1	1	1	1	1	1	1	1	1	1	1	0	0	0	0	0	0	0	0	0	1	1	1	1	1	1	1	1	0	1	1
NCI-H179	1	1	1	1	1	1	0	0	1	1	1	1	1	1	1	1	0	1	1	0	1	1	0	1	1	1	1	1	1	1	1	1	1	1	1	1	1	1	1	1	1	1	0	1	1	1	1	0	1	1
NCI-H183	0	0	1	0	1	1	0	1	0	0	1	1	0	1	1	1	1	1	1	0	0	0	1	1	1	1	1	1	1	1	0	0	0	0	0	0	0	0	0	1	1	1	0	1	1	1	1	1	1	1
NCI-H187	1	0	1	1	1	1	0	0	0	1	1	1	1	1	0	0	1	1	1	1	1	1	1	0	1	1	1	1	1	0	1	1	1	1	1	1	1	1	1	1	0	1	0	0	1	1	1	0	1	0
NCI-H194	0	0	0	1	1	1	1	0	1	1	0	1	0	0	1	1	1	1	1	1	0	1	0	1	1	1	1	1	1	1	1	1	1	1	1	1	1	1	1	1	1	1	1	1	1	0	0	0	1	0
NCI-H206	0	1	0	1	1	0	1	0	0	1	1	1	1	1	1	1	1	0	1	1	0	0	0	1	1	1	1	1	0	0	0	0	0	0	0	0	0	0	0	1	0	0	1	1	1	1	1	0	1	1
NCI-H211	0	1	1	1	0	0	1	1	0	0	1	1	1	1	1	1	0	1	1	1	0	0	1	1	1	1	1	1	1	1	1	1	1	1	1	1	1	1	1	1	1	0	1	1	1	1	1	1	1	1
NCI-H234	1	0	1	1	1	1	1	1	1	0	1	1	0	1	1	1	1	1	1	1	1	1	0	1	1	1	1	1	1	1	1	1	1	1	1	1	1	1	1	1	1	1	1	1	1	1	1	1	1	1
NCI-H441	1	0	1	1	1	1	1	0	0	1	0	0	1	0	1	0	0	1	1	1	0	1	1	1	1	1	1	1	1	1	1	1	1	1	1	1	1	1	1	0	1	1	0	1	1	1	1	0	1	0
NCI-H727	1	1	0	1	1	0	1	0	0	1	1	1	1	1	1	1	0	1	1	1	0	1	1	0	1	1	1	1	1	1	1	1	1	1	1	1	1	1	1	0	0	0	1	1	1	0	1	1	1	0
NCI-H835	1	1	1	0	1	1	1	1	1	1	0	1	1	1	1	1	1	1	1	1	1	1	1	1	1	1	1	1	1	1	0	0	0	0	0	0	0	0	0	1	0	1	1	1	1	1	1	1	1	1
OCUM-1	1	1	1	0	0	1	1	1	0	1	1	1	1	1	1	0	0	0	1	1	0	0	1	1	0	1	1	0	0	0	0	0	0	0	0	0	0	0	0	0	0	0	1	1	1	1	1	1	1	0
PANC-03-	0	0	0	1	0	0	1	1	0	1	1	0	0	1	0	0	0	1	0	1	0	0	1	0	1	0	0	0	0	0	0	0	0	0	0	0	0	0	0	0	1	1	1	1	0	0	1	1	0	0
RKN	0	1	1	1	0	0	1	1	0	1	0	1	0	0	1	0	0	0	1	1	1	0	1	0	0	1	1	1	1	1	0	0	0	0	0	0	0	0	0	0	0	1	0	0	1	0	1	1	1	0
SCH	0	1	0	1	0	1	1	0	1	0	1	0	0	1	0	0	0	0	1	0	0	1	0	1	1	0	0	0	0	0	1	1	1	1	1	0	1	1	0	0	1	0	1	0	1	1	1	1	1	1
SK-MEL-2	0	1	1	1	1	1	0	1	0	0	1	0	1	0	1	0	1	1	1	1	1	1	0	1	1	1	1	1	1	1	1	1	1	1	1	1	1	1	1	0	1	0	1	0	1	1	0	1	1	0
SK-MES-1	0	0	0	0	1	0	1	1	1	0	1	1	0	0	1	1	1	1	0	1	1	1	0	1	1	0	0	0	0	0	1	1	0	0	0	0	0	0	0	0	0	1	1	1	1	1	1	1	1	0
SKM-1	0	1	1	1	0	0	0	1	1	1	1	1	1	0	0	1	0	1	0	1	1	0	0	1	1	1	1	1	1	1	1	1	0	0	0	0	0	0	0	0	1	1	0	1	0	1	1	0	1	1
SN12C	1	1	0	1	1	0	1	0	0	0	1	1	1	0	1	1	1	0	1	0	1	0	1	1	1	0	0	0	0	0	1	1	1	1	1	1	1	1	1	1	0	1	1	1	1	1	1	1	1	1
Saos-2	1	1	1	1	1	0	1	1	0	1	0	1	0	1	0	0	0	1	1	1	0	1	1	0	0	1	1	1	1	1	0	0	0	0	0	0	0	0	0	1	1	1	0	0	0	0	0	1	1	1
TGBC24TK	1	1	0	1	1	0	0	1	1	0	1	1	0	0	1	0	0	1	1	1	1	1	0	0	0	1	1	1	1	1	1	1	1	0	0	0	0	0	0	0	0	1	1	1	1	1	1	0	1	0
THP-1	0	1	1	1	0	0	0	1	0	1	0	1	0	1	1	0	1	1	0	1	1	0	1	0	0	0	0	0	0	0	1	1	1	1	1	1	1	1	1	1	1	1	0	0	1	1	1	1	1	1
VMRC-LC	0	0	1	1	1	0	1	1	1	0	1	1	0	0	1	1	1	1	1	0	1	0	1	1	1	1	1	1	1	1	1	1	1	1	1	1	1	1	1	1	1	1	1	1	1	1	1	0	1	1
ZR-75-30	0	1	1	1	1	1	1	1	0	0	1	1	1	0	0	0	1	1	1	1	1	0	1	1	0	0	0	0	0	0	1	1	1	1	1	1	1	1	1	1	1	0	0	1	1	0	0	1	1	1

Sensitive cell lines

Sample n	ADGRV1	ANKRD36	ANKRD36	CSMD1	CSMD3	CUBN	DNAH11	DST	HECTD4	HLA-B	HMCN1	HYDIN	KIAA1671	KIR2DL1	LRP1B	LRP2	MACF1	MGAM	MUC16	MUC4	MUC5B	NBEA	NBPF1	NEB	OBSCN	PCDHA1	PCDHA2	PCDHA3	PCDHA4	PCDHA5	PCDHGA1	PCDHGA2	PCDHGA3	PCDHGA4	PCDHGA5	PCDHGA6	PCDHGB1	PCDHGB2	PCDHGB3	PCLO	PKHD1	PKHD1L1	PLEKHB2	RYR2	RYR3	SYNE1	TP53	TSPEAR	TTN	USH2A
A101D	0	1	0	0	1	0	0	0	0	0	0	1	1	0	1	0	0	0	1	0	0	0	1	0	1	0	0	0	0	0	0	0	0	0	0	0	0	0	0	0	0	1	1	1	1	0	0	1	1	0
A388	1	0	1	0	1	1	1	1	0	1	1	0	1	0	1	0	1	1	0	0	0	1	0	0	0	0	0	0	0	0	1	1	1	1	1	1	1	1	1	1	0	1	1	1	0	1	1	0	1	0
A704	0	1	1	1	1	0	0	1	1	0	0	1	1	1	1	1	0	1	0	1	1	0	0	1	0	0	0	0	0	0	1	1	1	1	1	1	1	1	1	0	1	0	1	1	0	0	1	0	1	0
AMO-1	0	1	0	1	1	1	1	1	1	1	1	1	1	0	1	1	1	1	1	1	0	1	1	0	0	1	1	1	1	1	1	1	1	1	1	1	1	1	1	1	1	1	1	1	1	1	0	1	1	0
BB30-HN	0	0	0	0	0	0	0	0	0	0	0	0	0	0	0	0	0	0	0	1	0	0	0	0	0	1	1	1	1	1	1	1	1	1	1	0	1	1	1	0	0	0	0	0	0	0	1	0	0	0
BB65-RCC	0	0	0	0	0	1	0	1	0	0	0	0	0	0	0	0	0	0	0	1	1	1	0	0	1	0	0	0	0	0	0	0	0	0	0	0	0	0	0	0	0	1	0	0	0	0	1	0	1	0
BE-13	1	1	0	1	1	0	1	0	1	1	1	1	1	1	1	1	1	0	0	1	1	1	1	1	1	1	1	1	1	1	1	1	1	1	1	1	1	1	1	1	1	1	1	0	0	0	1	1	1	1
CMK	1	1	0	1	0	1	1	0	0	0	1	1	1	0	1	1	1	1	1	1	0	1	1	0	1	1	1	1	0	0	1	1	1	1	1	1	1	1	1	1	0	1	1	0	1	1	1	1	1	1
CP66-MEL	1	0	1	1	0	0	1	1	1	1	1	1	1	1	1	1	1	1	1	0	1	1	0	1	1	1	1	1	1	1	1	1	1	1	1	1	1	1	1	1	1	0	1	1	1	1	0	1	1	1
D-247MG	0	0	0	1	0	0	0	0	1	0	1	1	1	1	0	1	0	1	1	1	1	1	1	0	0	1	1	0	0	0	0	0	0	0	0	0	0	0	0	1	0	1	1	1	0	1	0	1	1	0
D-336MG	1	0	1	0	1	0	1	0	0	1	1	1	1	0	0	1	0	0	0	1	1	0	1	1	0	1	1	1	1	1	0	0	0	0	0	0	0	0	0	1	1	0	1	1	0	1	1	1	1	0
D-542MG	0	0	0	0	0	0	0	0	0	0	1	0	0	0	0	0	0	0	0	0	0	0	0	1	0	1	1	1	1	1	1	1	1	1	0	0	1	1	0	0	0	0	0	0	0	0	1	0	1	1
DB	1	0	1	1	1	1	1	1	1	0	1	1	0	1	0	0	0	1	1	1	0	1	1	1	1	1	1	1	1	1	1	1	1	1	1	1	1	1	1	1	0	1	1	0	1	1	1	1	1	1
DEL	1	1	1	1	0	0	1	1	0	1	0	0	0	1	1	1	0	1	0	1	1	0	1	0	1	1	1	1	1	1	0	0	0	0	0	0	0	0	0	1	0	1	0	1	0	1	1	1	1	0
ECC12	0	0	1	0	1	1	1	1	1	1	0	0	1	0	0	0	1	0	0	1	0	1	1	0	1	1	0	0	0	0	0	0	0	0	0	0	0	0	0	0	1	1	1	1	1	1	1	1	1	1
EHEB	1	1	0	1	0	1	1	0	0	1	0	1	1	0	0	1	0	1	1	1	0	0	1	0	0	0	0	0	0	0	1	1	1	1	1	1	1	1	1	0	0	1	1	1	1	0	0	1	1	0
EKVX	1	1	0	1	0	1	0	1	0	0	0	1	0	1	1	1	1	1	0	1	0	0	0	1	1	1	1	1	1	1	0	0	0	0	0	0	0	0	0	0	1	1	0	1	1	1	1	1	1	1
Farage	1	1	1	1	1	0	0	1	1	1	0	1	1	1	1	1	1	0	1	1	0	1	1	1	1	1	1	1	1	1	1	1	1	1	1	1	1	1	1	1	1	1	1	1	1	1	1	1	1	1
HA7-RCC	1	0	0	0	0	0	0	0	1	0	1	0	0	0	0	0	1	0	1	0	1	0	0	0	1	0	0	0	0	0	0	0	0	0	0	0	0	0	0	0	1	0	0	0	1	1	0	0	1	0
HEL	0	0	0	1	1	0	0	0	0	1	0	1	1	1	0	1	1	0	0	1	0	0	1	0	0	0	0	0	0	0	0	0	0	0	0	0	0	0	0	1	0	0	0	1	0	1	1	0	1	1
HT	1	0	1	1	1	1	1	1	1	1	1	1	1	1	1	1	1	1	1	1	1	0	1	1	1	1	1	1	1	1	1	1	1	1	1	0	1	1	0	1	1	1	1	1	1	1	1	1	1	1
HUTU-80	1	1	1	1	1	1	1	0	0	0	1	1	1	1	1	1	1	1	0	1	0	0	1	1	0	1	1	1	1	1	0	0	0	0	0	0	0	0	0	1	1	0	1	0	0	1	0	1	1	1
JM1	0	1	0	0	0	0	1	1	1	0	1	1	1	1	1	1	0	0	0	1	0	0	0	0	1	0	0	0	0	0	1	1	1	0	0	0	1	0	0	1	0	0	1	1	1	1	0	0	0	1
KNS-42	1	0	0	0	0	1	0	1	0	1	0	0	1	0	1	0	0	1	1	1	1	1	1	1	1	0	0	0	0	0	0	0	0	0	0	0	0	0	0	0	1	0	0	0	1	1	1	0	1	1
KNS-81-F	1	1	1	0	0	0	1	0	0	1	1	1	0	0	0	1	0	0	0	1	1	1	0	1	0	1	1	1	1	1	1	1	1	1	1	1	1	1	1	1	1	1	0	1	1	1	0	0	1	1
LAN-6	0	1	1	0	0	1	1	0	0	1	1	1	1	1	0	0	1	1	1	0	0	0	0	1	0	0	0	0	0	0	0	0	0	0	0	0	0	0	0	0	0	1	1	0	1	0	1	1	1	0
LB1047-R	0	1	0	1	1	1	0	1	0	0	0	0	0	0	0	0	0	0	0	0	0	0	0	0	0	0	0	0	0	0	0	0	0	0	0	0	0	0	0	0	0	0	0	0	0	0	0	0	0	0
LB2241-R	0	0	0	0	0	1	0	0	0	0	0	0	0	0	0	0	0	0	1	0	0	0	0	0	0	0	0	0	0	0	1	1	1	1	1	1	1	1	1	0	0	0	0	0	0	0	0	0	1	0
LB2518-M	1	0	0	1	0	1	1	1	1	0	0	1	0	0	1	0	0	1	1	1	0	0	0	0	1	0	0	0	0	0	1	1	0	0	0	0	0	0	0	1	1	1	1	0	0	1	0	1	1	1
LB373-ME	0	1	0	0	0	0	0	0	0	0	1	1	0	0	0	1	0	0	0	0	0	0	0	0	1	1	1	1	1	1	0	0	0	0	0	0	0	0	0	1	0	0	1	0	1	0	0	0	1	0
LC-2-ad	0	0	0	1	1	0	1	0	1	1	1	1	1	1	0	0	1	1	1	1	1	1	1	1	1	0	0	0	0	0	1	1	1	1	1	1	1	1	1	1	0	1	1	1	1	1	1	1	1	1
MMAC-SF	0	1	1	1	1	1	1	0	0	1	0	1	1	0	0	0	0	0	1	0	0	0	0	0	1	0	0	0	0	0	1	1	0	0	0	0	0	0	0	0	0	0	1	0	1	1	0	1	1	1
MY-M12	1	1	0	1	1	1	1	1	0	1	0	1	1	1	0	0	1	0	1	1	1	0	1	1	0	1	1	1	1	1	1	1	1	1	1	1	1	1	1	0	0	1	0	0	1	1	0	1	1	0
MZ2-MEL	1	0	1	0	1	0	0	0	1	1	0	1	1	0	1	1	1	1	1	1	1	1	1	1	1	1	1	1	1	1	1	1	1	1	1	1	1	1	1	0	0	1	1	0	1	0	0	1	1	0
MZ7-mel	1	1	1	1	1	1	1	1	1	1	1	1	1	0	1	1	1	0	1	1	1	1	0	1	1	1	1	1	1	1	1	1	1	1	1	1	1	1	1	1	1	1	1	1	1	1	0	1	1	1
NK-92MI	0	1	1	0	1	0	0	1	0	0	0	0	1	1	1	1	1	1	1	1	0	1	1	1	1	1	1	1	1	1	1	1	1	0	0	0	1	0	0	1	1	1	1	0	0	1	1	0	1	1
NU-DUL-1	0	0	0	1	1	1	1	1	0	0	0	0	1	0	1	0	0	1	0	1	1	1	1	1	1	1	1	1	1	1	1	1	1	1	1	1	1	1	1	1	1	1	0	1	0	1	1	0	1	1
OCI-AML5	1	0	1	1	1	1	0	1	1	0	0	0	1	0	1	0	1	0	0	1	0	1	1	1	0	1	1	1	1	1	1	1	1	1	0	0	1	1	0	1	1	1	1	1	1	0	0	0	1	0
OCI-LY-19	1	1	1	0	1	1	1	0	0	1	0	1	0	0	0	1	0	0	0	1	0	0	0	1	0	0	0	0	0	0	1	1	1	1	1	1	1	1	1	0	0	0	0	0	0	0	0	0	1	0
OCI-M1	1	0	1	1	1	1	0	1	0	0	1	1	1	1	1	1	0	0	0	1	1	0	0	0	1	1	1	1	1	0	0	0	0	0	0	0	0	0	0	1	1	0	1	0	1	1	1	0	1	0
QIMR-WI	0	0	0	0	0	1	1	1	1	0	1	1	0	0	1	0	0	1	0	1	0	0	0	0	0	1	1	1	1	1	1	1	1	1	1	1	1	1	1	0	1	0	0	1	1	1	0	1	0	0
RXF393	1	1	1	1	1	0	1	0	1	0	0	1	1	1	0	0	0	0	1	0	0	1	1	1	1	0	0	0	0	0	1	1	1	1	1	1	1	1	1	0	1	0	1	1	0	0	1	1	1	0
SF126	1	0	1	1	0	0	0	0	1	0	0	1	0	1	1	0	1	1	0	1	1	1	1	1	0	1	1	1	1	1	0	0	0	0	0	0	0	0	0	0	0	0	1	0	1	0	1	1	1	0
SNB75	1	1	1	1	1	1	1	0	1	0	0	1	0	1	0	1	0	1	1	1	1	1	1	1	1	0	0	0	0	0	1	1	1	1	1	1	1	1	1	0	1	0	0	0	0	0	1	0	1	0
SU-DHL-1	0	0	0	1	1	0	1	1	0	0	1	0	1	1	0	0	1	0	0	1	0	0	0	1	0	1	1	0	0	0	0	0	0	0	0	0	0	0	0	1	1	0	1	0	1	1	1	1	0	0
SU-DHL-4	1	1	1	1	1	0	1	0	1	1	1	1	0	1	1	1	1	0	1	1	1	1	0	1	1	1	1	1	1	1	1	1	1	1	1	1	1	1	1	0	1	0	1	0	0	0	1	0	1	0
TE-10	1	1	0	0	0	0	0	0	1	1	1	1	0	0	1	1	1	0	0	1	0	1	1	0	1	0	0	0	0	0	0	0	0	0	0	0	0	0	0	1	0	1	0	0	1	1	1	1	1	0
no-10	0	0	1	0	1	0	0	1	0	1	1	1	0	0	1	0	1	1	0	1	1	0	0	1	0	1	1	1	1	1	1	1	1	1	1	1	1	1	1	1	1	1	0	0	0	1	1	1	0	1
no-11	1	1	1	0	0	0	1	1	0	0	1	1	0	0	1	0	0	0	1	1	1	0	0	1	0	1	1	1	0	0	0	0	0	0	0	0	0	0	0	1	1	0	1	0	0	0	1	1	1	1

Average mutated genes per cell line in each cell line group

	ADGRV1	ANKRD36	ANKRD36	CSMD1	CSMD3	CUBN	DNAH11	DST	HECTD4	HLA-B	HMCN1	HYDIN	KIAA1671	KIR2DL1	LRP1B	LRP2	MACF1	MGAM	MUC16	MUC4	MUC5B	NBEA	NBPF1	NEB	OBSCN	PCDHA1	PCDHA2	PCDHA3	PCDHA4	PCDHA5	PCDHGA1	PCDHGA2	PCDHGA3	PCDHGA4	PCDHGA5	PCDHGA6	PCDHGB1	PCDHGB2	PCDHGB3	PCLO	PKHD1	PKHD1L1	PLEKHB2	RYR2	RYR3	SYNE1	TP53	TSPEAR	TTN	USH2A
Average i	0,52778	0,66667	0,77778	0,75	0,72222	0,5	0,66667	0,63889	0,41667	0,52778	0,75	0,88889	0,5	0,41667	0,72222	0,61111	0,55556	0,75	0,77778	0,83333	0,52778	0,52778	0,61111	0,75	0,75	0,72222	0,72222	0,69444	0,66667	0,63889	0,66667	0,66667	0,58333	0,52778	0,5	0,47222	0,55556	0,5	0,47222	0,52778	0,58333	0,63889	0,66667	0,77778	0,83333	0,77778	0,80556	0,63889	0,91667	0,69444
Average s	0,57143	0,5102	0,5102	0,57143	0,57143	0,4898	0,57143	0,5102	0,44898	0,46939	0,5102	0,71429	0,59184	0,44898	0,55102	0,5102	0,4898	0,46939	0,4898	0,77551	0,44898	0,46939	0,5102	0,57143	0,55102	0,61224	0,59184	0,55102	0,5102	0,4898	0,61224	0,61224	0,57143	0,53061	0,4898	0,44898	0,57143	0,53061	0,46939	0,55102	0,53061	0,53061	0,63265	0,44898	0,55102	0,61224	0,59184	0,59184	0,87755	0,44898

Mutation frequence between insensitive (36) and sensitive (49) cell lines. The intermediate cell lines were excluded in this analysis (922)

p-value	0,69342	0,15272	0,01151	0,09071	0,1572	0,92701	0,37933	0,24211	0,76984	0,59987	0,02496	0,05238	0,40612	0,76984	0,11	0,36115	0,55434	0,009	0,0067	0,51594	0,47843	0,59987	0,36115	0,09071	0,06078	0,29623	0,21864	0,18421	0,15272	0,17595	0,61158	0,61158	0,91389	0,97967	0,92701	0,83416	0,88574	0,7833	0,97967	0,83416	0,63391	0,32394	0,74932	0,00203	0,00581	0,10777	0,03684	0,66461	0,56791	0,02446
Genes	ADGRV1	ANKRD36	ANKRD36	CSMD1	CSMD3	CUBN	DNAH11	DST	HECTD4	HLA-B	HMCN1	HYDIN	KIAA1671	KIR2DL1	LRP1B	LRP2	MACF1	MGAM	MUC16	MUC4	MUC5B	NBEA	NBPF1	NEB	OBSCN	PCDHA1	PCDHA2	PCDHA3	PCDHA4	PCDHA5	PCDHGA1	PCDHGA2	PCDHGA3	PCDHGA4	PCDHGA5	PCDHGA6	PCDHGB1	PCDHGB2	PCDHGB3	PCLO	PKHD1	PKHD1L1	PLEKHB2	RYR2	RYR3	SYNE1	TP53	TSPEAR	TTN	USH2A
