# Supplementary material for: Multi-omics analysis identifies repurposing bortezomib in the treatment of kidney-, nervous system-, and hematological cancers
Source: Sci Rep. 2024 Aug 10;14:18576. doi: 10.1038/s41598-024-62339-x (PMC11316778; doi:10.1038/s41598-024-62339-x)
Supplement: Supplementary file 5 — Supplementary Table 4. [file 41598_2024_62339_MOESM5_ESM.pdf]

Supplementary Table 4: Examination of the 19 most mutated genes in the CCLE dataset in terms of their mutation frequency

1= Mutated gene
0= No mutated gene

Bortezomib-insensitive cell lines

	STUDY_ID	Cell_lines	Organ syst	ANKRD36(	FSIP2	HERC2	HMCN1	MGAM	MUC12	MUC16	PCDH15	PDE4DIP	PTPRN2	RIMS2	RYR1	RYR2	RYR3	SPTA1	TP53	TRRAP	USH2A	WDFY4
1	ccle_broa(	ASPC1	PANCREAS	1	0	0	0	0	0	0	0	0	0	0	0	0	0	0	1	0	1	1
2	ccle_broa(	BT474	BREAST	0	0	1	0	0	1	1	1	1	0	0	1	0	1	0	1	0	0	0
3	ccle_broa(	COLO680N	OESOPHA(	0	0	0	1	0	0	0	0	0	0	0	0	0	0	0	1	0	1	0
4	ccle_broa(	CORL88	LUNG	0	0	0	1	0	0	0	1	0	0	0	0	1	0	1	1	1	1	0
5	ccle_broa(	DMS79	LUNG	1	0	0	1	1	0	1	0	0	0	1	1	1	1	0	1	0	0	0
6	ccle_broa(	JHH6	LIVER	0	0	0	0	0	0	0	0	0	0	0	0	1	0	0	1	0	0	1
7	ccle_broa(	NCIH1435	LUNG	0	1	0	1	0	1	1	0	0	0	0	0	1	1	1	1	0	1	1
8	ccle_broa(	NCIH1563	LUNG	0	0	1	0	0	0	1	0	0	0	0	0	0	1	0	0	0	1	0
9	ccle_broa(	NCIH1651	LUNG	1	0	0	1	0	0	1	1	1	0	1	1	1	1	1	1	0	0	0
10	ccle_broa(	NCIH1793	LUNG	0	1	0	0	0	1	1	1	1	0	1	0	1	1	0	1	1	1	1
11	ccle_broa(	NCIH1838	LUNG	1	1	1	0	1	0	1	1	0	1	1	0	1	0	1	1	0	1	1
12	ccle_broa(	NCIH1944	LUNG	0	0	0	0	0	0	0	0	0	0	0	0	0	0	0	0	0	0	0
13	ccle_broa(	NCIH2066	LUNG	0	0	0	1	0	0	0	0	0	0	0	1	1	0	0	1	0	1	0
14	ccle_broa(	NCIH211	LUNG	0	0	0	0	0	1	1	0	0	0	0	0	0	0	0	1	0	1	0
15	ccle_broa(	NCIH2342	LUNG	0	0	1	1	0	1	1	1	0	1	0	0	1	1	1	1	1	1	1
16	ccle_broa(	NCIH441	LUNG	0	0	0	0	1	0	1	0	0	0	1	1	1	0	0	1	1	0	0
17	ccle_broa(	NCIH727	LUNG	0	1	0	0	0	0	1	1	0	0	1	1	0	0	0	1	0	0	0
18	ccle_broa(	OCUM1	STOMACH	0	0	0	0	0	1	1	0	0	1	0	0	0	0	0	0	0	0	0
19	ccle_broa(	PANC0327	PANCREAS	0	0	0	0	0	1	1	0	0	0	0	0	1	0	0	0	0	0	0
20	ccle_broa(	RKN	SOFT	0	1	0	0	0	0	1	0	0	0	0	1	0	0	0	1	0	0	0
21	ccle_broa(	SKM1	HAEMATO	0	0	0	0	0	0	0	0	0	0	0	0	0	0	1	1	0	1	0
22	ccle_broa(	SKMEL1	SKIN	0	0	0	0	1	0	1	0	0	0	1	0	0	0	0	0	0	0	0
23	ccle_broa(	SKMEL24	SKIN	0	0	0	0	0	0	1	0	0	0	0	0	0	1	0	0	0	0	0
24	ccle_broa(	SKMES1	LUNG	0	0	0	0	0	1	0	0	0	0	0	0	0	0	1	1	0	0	0
25	ccle_broa(	THP1	HAEMATO	1	1	1	0	0	0	0	0	0	0	0	0	0	0	0	1	0	0	0
26	ccle_broa(	VMRCLCD	LUNG	0	1	1	1	1	0	0	1	1	0	1	1	0	0	0	1	0	1	0
27	ccle_broa(	ZR7530	BREAST	0	0	0	0	0	0	1	0	0	0	0	0	0	0	0	0	0	1	0

Bortezomib-sensitive cell lines

	STUDY_ID	Cell_lines	Organ syst	ANKRD36(	FSIP2	HERC2	HMCN1	MGAM	MUC12	MUC16	PCDH15	PDE4DIP	PTPRN2	RIMS2	RYR1	RYR2	RYR3	SPTA1	TP53	TRRAP	USH2A	WDFY4
1	ccle_broa(	A101D	SKIN	0	0	0	1	0	0	0	0	0	0	0	0	1	0	0	0	0	0	0
2	ccle_broa(	A704	KIDNEY	0	0	1	0	0	1	0	0	0	1	0	1	0	0	1	1	0	0	0
3	ccle_broa(	AMO1	HAEMATO	0	0	0	1	0	0	0	0	1	0	0	0	0	0	0	0	0	0	0
4	ccle_broa(	CMK	HAEMATO	0	0	0	0	0	0	1	0	0	0	0	0	1	0	0	1	0	1	0
5	ccle_broa(	DB	HAEMATO	0	1	0	0	0	0	1	1	0	0	0	0	0	1	0	1	0	1	0
6	ccle_broa(	DEL	HAEMATO	0	0	0	0	0	0	0	0	0	0	0	0	1	0	0	1	0	0	0
7	ccle_broa(	ECC12	STOMACH	0	1	0	0	0	0	0	0	0	0	0	0	0	0	0	1	0	0	0
8	ccle_broa(	EHEB	HAEMATO	1	0	0	0	0	0	1	0	0	0	0	0	0	0	0	0	0	0	0
9	ccle_broa(	EKVX	LUNG	0	1	0	0	0	0	0	1	1	0	0	0	0	1	0	1	0	1	0
10	ccle_broa(	HEL	HAEMATO	0	0	0	0	0	0	0	0	0	0	0	0	0	0	0	1	0	1	0
11	ccle_broa(	HT	HAEMATO	0	1	0	0	0	0	1	1	1	1	0	0	0	1	0	1	1	0	1
12	ccle_broa(	HUTU80	SMALL	0	0	0	0	0	0	0	1	0	0	0	0	0	0	0	0	0	0	0
13	ccle_broa(	JM1	HAEMATO	0	0	0	0	0	0	1	0	0	0	1	0	0	0	0	0	0	0	0
14	ccle_broa(	KNS42	CENTRAL	0	0	0	0	0	1	0	0	0	0	0	0	0	0	0	1	0	1	0
15	ccle_broa(	NUDUL1	HAEMATO	0	1	0	0	0	0	0	0	0	0	0	0	0	0	0	1	0	1	0
16	ccle_broa(	OCIAML5	HAEMATO	0	0	0	0	0	0	0	0	0	0	0	0	0	0	0	0	0	0	0
17	ccle_broa(	OCILY19	HAEMATO	0	0	0	0	0	0	0	0	0	0	0	0	0	0	0	0	0	0	0
18	ccle_broa(	OCIM1	HAEMATO	0	0	0	1	0	0	0	0	0	0	0	0	0	0	0	1	0	0	0
19	ccle_broa(	SF126	CENTRAL	0	0	0	0	1	0	0	0	0	0	1	0	0	1	0	0	0	0	0
20	ccle_broa(	SNB75	CENTRAL	0	0	0	0	0	0	0	0	0	0	0	0	0	0	0	1	0	0	0
21	ccle_broa(	SUDHL4	HAEMATO	0	0	0	1	0	0	0	1	0	0	0	0	0	0	0	1	1	0	0
22	ccle_broa(	TE10	OESOPHA(	0	0	0	0	0	0	0	0	1	0	0	0	0	0	0	1	0	0	0

Average mutated genes per cell line in each cell line group

Average bortezomib-insensitive	0,185185	0,259259	0,222222	0,296296	0,185185	0,296296	0,62963	0,296296	0,148148	0,111111	0,296296	0,296296	0,407407	0,296296	0,259259	0,740741	0,148148	0,481481	0,222222
Average bortezomib-sensitive	0,045455	0,227273	0,045455	0,181818	0,045455	0,045455	0,227273	0,227273	0,227273	0,090909	0,045455	0,090909	0,136364	0,181818	0,045455	0,636364	0,090909	0,272727	0,045455

Mutation frequency between bortezomib-insensitive (27) and sensitive (22) cell lines.

p-value	0,143603	0,800746	0,081601	0,364432	0,143603	0,023955	0,004157	0,595358	0,487119	0,820866	0,023955	0,07886	0,037273	0,364432	0,044998	0,440933	0,552922	0,141517	0,081601
Genes	ANKRD36(	FSIP2	HERC2	HMCN1	MGAM	MUC12	MUC16	PCDH15	PDE4DIP	PTPRN2	RIMS2	RYR1	RYR2	RYR3	SPTA1	TP53	TRRAP	USH2A	WDFY4
