# Supplementary material for: Multi-omics analysis identifies repurposing bortezomib in the treatment of kidney-, nervous system-, and hematological cancers
Source: Sci Rep. 2024 Aug 10;14:18576. doi: 10.1038/s41598-024-62339-x (PMC11316778; doi:10.1038/s41598-024-62339-x)
Supplement: Supplementary file 6 — Supplementary Table 5. [file 41598_2024_62339_MOESM6_ESM.pdf]

Supplementray Table 5. Seven bortezomib sensitivity genes that differ between MM patients

RPS4Y1	ENSG00000129824	RPS4Y1	ribosomal protein S4 Y-linked 1 [Source:HGNC Symbol;Acc:HGNC:10425]	ENTREZGENE,GENECARDS,HGNC,UNIPROT_GN,WIKIGENE
DDX3Y	ENSG00000067048	DDX3Y	DEAD-box helicase 3 Y-linked [Source:HGNC Symbol;Acc:HGNC:2699]	ENTREZGENE,GENECARDS,HGNC,UNIPROT_GN,WIKIGENE
KDM5D	ENSG00000012817	KDM5D	lysine demethylase 5D [Source:HGNC Symbol;Acc:HGNC:11115]	ENTREZGENE,GENECARDS,HGNC,UNIPROT_GN,WIKIGENE
ZFY	ENSG00000067646	ZFY	zinc finger protein Y-linked [Source:HGNC Symbol;Acc:HGNC:12870]	ENTREZGENE,GENECARDS,HGNC,UNIPROT_GN,WIKIGENE
USP9Y	ENSG00000114374	USP9Y	ubiquitin specific peptidase 9 Y-linked [Source:HGNC Symbol;Acc:HGNC:12633]	ENTREZGENE,GENECARDS,HGNC,UNIPROT_GN,WIKIGENE
EIF1AY	ENSG00000198692	EIF1AY	eukaryotic translation initiation factor 1A Y-linked [Source:HGNC Symbol;Acc:HGNC:3252]	ENTREZGENE,GENECARDS,HGNC,UNIPROT_GN,WIKIGENE
UTY	ENSG00000183878	UTY	ubiquitously transcribed tetratricopeptide repeat containing, Y-linked [Source:HGNC Symbol;Acc:HGNC:12638]	ENTREZGENE,GENECARDS,HGNC,UNIPROT_GN,WIKIGENE
