# Supplementary material for: Multi-omics analysis identifies repurposing bortezomib in the treatment of kidney-, nervous system-, and hematological cancers
Source: Sci Rep. 2024 Aug 10;14:18576. doi: 10.1038/s41598-024-62339-x (PMC11316778; doi:10.1038/s41598-024-62339-x)
Supplement: Supplementary file 7 — Supplementary Table 6. [file 41598_2024_62339_MOESM7_ESM.pdf]

Supplementary Table 6. Genes identified using limma and genes identified in bortezomib-insensitive and bortezomib-sensitive cell lines

	logFC	AveExpr	t	P,Value	adj P Val	B
06-sep	1,39111	0,027709	3,688309	0,00022575	0,017384	0,393739
1-Mar#1	-1,77773	0,047461	-4,71338	2,44E-06	0,002329	4,435958
ABCB5	1,482832	0,010753	3,931494	8,44E-05	0,011152	1,263465
ABCC4	1,281081	0,013695	3,396584	0,000682329	0,029564	-0,57635
ACOT4	-1,32268	0,049536	-3,50687	0,000453404	0,023841	-0,21898
ACSL4	1,232004	0,001218	3,266462	0,001089006	0,039198	-0,98328
ACTA2	1,514242	0,032095	4,014773	5,95E-05	0,009536	1,574063
ACTL6B	-1,24739	0,039773	-3,30726	0,000942135	0,036274	-0,8574
ADAMTSL3	1,593038	0,03192	4,223687	2,40E-05	0,007064	2,381884
AGAP1	-2,08851	0,040578	-5,53736	3,07E-08	0,000125	8,400359
AIM2	1,300364	0,054076	3,44771	0,000565362	0,025952	-0,41211
AKAP1	-1,23415	0,047905	-3,27215	0,001067339	0,039059	-0,96583
AKR1C2	-1,2131	0,064923	-3,21633	0,001298404	0,043671	-1,13581
ALDH18A1	1,243208	-0,00479	3,296169	0,000980132	0,037035	-0,89177
ALPG	-1,26618	0,036883	-3,35708	0,00078771	0,032567	-0,70157
ALPK2	1,262321	0,014737	3,346844	0,000817375	0,033446	-0,73378
AMMECR1	1,190793	-0,00953	3,1572	0,001592926	0,048182	-1,3127
ANK3	-1,20375	0,071723	-3,19156	0,00141505	0,045438	-1,2103
ANKEF1	-1,18991	0,057245	-3,15485	0,001605825	0,048277	-1,31967
ANKRD44	1,346962	0,023488	3,571257	0,000355273	0,021488	-0,00509
ANXA9	-1,50616	0,036966	-3,99335	6,51E-05	0,009536	1,493555
AP1M1	1,62141	-0,03337	4,298912	1,72E-05	0,005934	2,682795
AP5M1	-1,22046	0,008958	-3,23587	0,00121274	0,04189	-1,07665
APEX1	-1,37596	-0,02663	-3,64815	0,000264137	0,018829	0,255453
APOBEC3D	1,456445	0,008782	3,861534	0,000112678	0,012454	1,007572
APOBEC3G	1,245693	0,033055	3,302758	0,00095739	0,03655	-0,87137
APOBEC3H	1,184477	0,038163	3,140453	0,001686869	0,049298	-1,3622
APOL3	1,537844	0,029959	4,077349	4,56E-05	0,008963	1,811732
ARFGEF3	-1,32378	0,069825	-3,50979	0,000448466	0,023691	-0,20939
ARHGAP32	-1,52187	0,052621	-4,03499	5,46E-05	0,009536	1,650459
ARHGEF16	-1,54687	0,052755	-4,10129	4,11E-05	0,008901	1,903642
ARHGEF19	-1,38175	0,053406	-3,66349	0,000248799	0,018459	0,308111
ARHGEF26	-1,43549	0,035851	-3,80597	0,000141251	0,013875	0,807602
ARHGEF5	-1,34534	0,042147	-3,56696	0,00036115	0,021502	-0,0195
ARHGEF6	1,617615	0,035676	4,288849	1,80E-05	0,005955	2,642232
ARID5B	1,201516	-0,02456	3,185629	0,0014444	0,045802	-1,22807
ARMCX5	-1,21555	0,005872	-3,22284	0,001269248	0,043119	-1,11613
ARPC1A	-1,29921	0,007152	-3,44464	0,00057182	0,025952	-0,42203
ARPC2	1,19517	-0,03187	3,168805	0,001530674	0,047192	-1,27824
ARSK	1,385758	0,005335	3,674118	0,000238674	0,018056	0,344699
ASIC2	-1,23115	0,046481	-3,2642	0,001097726	0,0392	-0,9902
ASPH	-1,19538	0,021476	-3,16935	0,001527805	0,047192	-1,27662
ASRGL1	-1,18569	0,023055	-3,14366	0,001668481	0,049143	-1,35274
ATAD2	-1,18563	-0,02969	-3,14352	0,001669281	0,049143	-1,35315
ATM	1,434271	0,011682	3,802744	0,000143103	0,013875	0,796087
ATP11C	1,397227	-0,02706	3,704526	0,000211788	0,016704	0,450008
ATP6V1C2	-1,42639	0,053829	-3,78184	0,000155675	0,014203	0,721666
ATP9A	-1,42056	0,029401	-3,7664	0,000165619	0,014698	0,666974
ATXN7	1,612192	-0,00741	4,274471	1,92E-05	0,006104	2,584443
BAIAP2L1	-1,31834	0,030805	-3,49536	0,00047342	0,024544	-0,25682
BCL2A1	1,532274	0,007833	4,062581	4,85E-05	0,009064	1,755312
BDH2	1,35775	0,038906	3,599858	0,000318392	0,020725	0,091172
BGN	1,291457	0,006419	3,424094	0,000616854	0,027459	-0,48828
BPIFA1	1,185617	0,013426	3,143475	0,001669547	0,049143	-1,35329
BRMS1L	-1,25018	0,026656	-3,31464	0,0009176	0,035583	-0,83445
BRPF3	-1,3785	0,030052	-3,65488	0,000257304	0,018664	0,27852
BSPRY	-1,58531	0,064211	-4,2032	2,63E-05	0,007314	2,300863
BTAF1	1,237714	0,016171	3,281604	0,001032187	0,038029	-0,93674
BTK	1,214724	0,029608	3,220648	0,001279014	0,043205	-1,12277
BTN2A2	1,799542	0,000991	4,7712	1,83E-06	0,002289	4,693373
BTN3A1	1,523649	0,031455	4,039713	5,35E-05	0,009452	1,668347
BTN3A2	1,451122	-0,03149	3,847421	0,000119368	0,012676	0,956506
BTN3A3	1,537544	0,015944	4,076555	4,57E-05	0,008963	1,808692
C11orf80	-1,43103	0,020836	-3,79416	0,000148145	0,013956	0,765481
C16orf54	1,189412	0,046842	3,153539	0,001613041	0,048277	-1,32355
C19orf33	-1,30524	0,055645	-3,46062	0,000538927	0,025604	-0,37023
C19orf53	1,263273	-0,01677	3,349367	0,000809965	0,033317	-0,72584
C19orf66	1,778461	-0,00975	4,715307	2,41E-06	0,002329	4,444506
C1orf115	-1,40365	0,014881	-3,72155	0,000198003	0,016108	0,509353
C1orf226	-1,43115	0,051342	-3,79446	0,000147968	0,013956	0,766536
C2orf15	-1,6426	0,062074	-4,3551	1,33E-05	0,004912	2,911024
C3orf14	-1,2108	0,015284	-3,21024	0,001326236	0,044135	-1,15418

C5orf15	1,431774	-0,0085	3,796123	0,000146977	0,013956	0,772474
C7orf61	1,530687	0,042405	4,058375	4,94E-05	0,009124	1,73928
CABYR	-1,32209	0,045759	-3,5053	0,000456093	0,023905	-0,22416
CALM1	-1,29783	0,012074	-3,441	0,000579579	0,026014	-0,43381
CAMSAP3	-1,50098	0,056574	-3,97961	6,90E-05	0,010014	1,442139
CARM1	1,220564	0,001672	3,236132	0,001211615	0,04189	-1,07585
CASP1	1,369595	0,026987	3,631263	0,000282038	0,019372	0,197759
CCDC130	1,301416	-0,00224	3,450498	0,000559554	0,025952	-0,40308
CCND2	1,192867	0,030062	3,162698	0,001563144	0,047738	-1,29639
CCNH	1,252961	-0,03223	3,322028	0,000893659	0,035073	-0,81145
CD47	1,235182	0,010186	3,27489	0,001057034	0,038769	-0,9574
CD48	1,443884	0,035655	3,828229	0,000129069	0,01317	0,887366
CD5L	1,274571	-0,02365	3,379323	0,000726647	0,030988	-0,63124
CD70	1,268954	0,000144	3,364431	0,000767018	0,031955	-0,67838
CD72	1,314477	0,02419	3,485128	0,000491903	0,024744	-0,29035
CD74	1,621833	0,028607	4,300034	1,71E-05	0,005934	2,687321
CD81	1,398108	-0,03997	3,706862	0,000209844	0,016632	0,458136
CDC42EP2	-1,34971	0,030908	-3,57855	0,00034551	0,021345	0,019372
CDK6	1,708273	-0,00398	4,529214	5,92E-06	0,003103	3,637058
CEACAM6	-1,41612	0,05612	-3,75461	0,000173613	0,014948	0,625346
CELF4	-1,29928	0,052033	-3,44484	0,000571391	0,025952	-0,42138
CENPX_EN	-1,39492	0,014685	-3,69842	0,000216947	0,016947	0,42879
CERS6	-1,45994	0,020929	-3,87079	0,000108485	0,012241	1,041154
CFD	-1,41937	0,053633	-3,76324	0,000167728	0,014698	0,655798
CGGBP1	1,305981	-0,01466	3,462602	0,00053498	0,025506	-0,3638
CGN	-1,69094	0,049443	-4,48326	7,35E-06	0,003124	3,442668
CHEK2_EN	-1,27165	0,066543	-3,37158	0,000747382	0,03157	-0,65577
CHI3L2	1,460488	0,020279	3,872252	0,000107835	0,012241	1,046476
CHMP4C	-1,26058	0,053488	-3,34222	0,000831122	0,033592	-0,7483
CHN2	-1,22437	0,06484	-3,24623	0,001169459	0,040977	-1,04513
CHST2	1,44309	0,012301	3,826126	0,000130176	0,01317	0,87981
CLDN3	-1,50861	0,072693	-3,99985	6,34E-05	0,009536	1,517926
CLDN4	-1,23183	0,054716	-3,26601	0,001090745	0,039198	-0,98466
CLINT1	1,272744	0,000846	3,374479	0,000739558	0,031456	-0,6466
CLK4	1,232744	0,029938	3,268425	0,001081481	0,039198	-0,97726
CLPSL2	-1,20186	0,030557	-3,18653	0,001439896	0,045789	-1,22537
CMTM4	-1,69993	0,02775	-4,50709	6,57E-06	0,003124	3,54324
CNKSR1	-1,43727	0,052921	-3,81069	0,000138581	0,01373	0,824476
COBL	-1,58347	0,053158	-4,19831	2,69E-05	0,007314	2,281574
COL4A3BP	1,300699	0,011311	3,448598	0,000563507	0,025952	-0,40923
COQ6	-1,49278	0,021455	-3,95787	7,56E-05	0,010543	1,361122
CPE	-1,33849	0,043024	-3,54879	0,000387	0,022063	-0,08016
CPED1	1,359465	0,029463	3,604407	0,000312867	0,020584	0,106553
CPOX	1,359054	0,013209	3,603317	0,000314183	0,020584	0,102864
CR1L	1,617673	0,027203	4,289005	1,79E-05	0,005955	2,64286
CRY1	-1,25192	-0,02429	-3,31926	0,000902581	0,035168	-0,82009
CRYAB	1,317826	0,024551	3,494007	0,00047583	0,024544	-0,26126
CRYGS	1,468948	0,016347	3,894684	9,83E-05	0,012171	1,128251
CSK	1,256063	0,016687	3,330253	0,000867672	0,034385	-0,78577
CTSV_ENST	-1,29924	0,022993	-3,44472	0,000571655	0,025952	-0,42178
CXADR	-1,46048	0,029381	-3,87222	0,000107849	0,012241	1,046363
CXorf38	1,354078	0,030021	3,590122	0,000330524	0,020735	0,058316
CYB5A	-1,24022	-0,00738	-3,28826	0,001008105	0,037523	-0,91623
CYLD	1,37926	0,0216	3,656889	0,000255296	0,018664	0,285417
CYTL1	1,232503	0,047255	3,267786	0,001083924	0,039198	-0,97922
DDO	1,587156	0,045201	4,208093	2,58E-05	0,007314	2,320173
DGKA	1,218214	0,019897	3,229901	0,001238333	0,042448	-1,09476
DIMT1	1,238872	-0,02159	3,284673	0,001021011	0,037789	-0,92728
DMXL1	1,491657	0,010485	3,954892	7,66E-05	0,010543	1,350072
DPY30	-1,3481	0,020898	-3,57428	0,000351195	0,021435	0,005043
DPYD	1,192667	0,042487	3,162169	0,00156599	0,047738	-1,29796
DRG2	1,195952	0,009401	3,170877	0,001519799	0,047125	-1,27208
DSP	-1,55392	0,041641	-4,11997	3,79E-05	0,008618	1,975714
DVL1	-1,44573	0,011569	-3,83312	0,000126529	0,013094	0,904955
EAPP	-1,55007	0,026192	-4,10977	3,96E-05	0,008696	1,936301
EBAG9	-1,2584	0,040691	-3,33644	0,000848596	0,034044	-0,76642
ECT2	-1,30035	-0,04066	-3,44768	0,000565428	0,025952	-0,41221
EIF2D	1,369136	-0,01742	3,630048	0,000283369	0,019372	0,193618
EIF3G	1,197832	-0,00611	3,175862	0,001493924	0,047013	-1,25723
EIF4G2	-1,4531	-0,02353	-3,85265	0,000116845	0,01249	0,975423
EIF5	-1,22032	0,010237	-3,23549	0,001214329	0,04189	-1,07779
EIF5A	1,208707	0,002033	3,204696	0,001352054	0,044391	-1,17088
ELF3	-1,36714	0,060392	-3,62477	0,000289223	0,019545	0,175636
ELP2	1,227867	0,003808	3,255495	0,00113195	0,039982	-1,01685
EMP2	-1,3546	0,034572	-3,59151	0,00032877	0,020735	0,062989

ENPP5	-1,32585	0,040351	-3,51529	0,000439281	0,023584	-0,19126
ENTPD4	1,262867	0,022549	3,348292	0,000813114	0,033362	-0,72922
EOGT	1,22208	0,005335	3,240151	0,001194665	0,041476	-1,06363
EPB41L4B	-1,84519	0,03711	-4,89222	9,97E-07	0,00162	5,242287
EPB41L5	-1,27156	0,047038	-3,37133	0,000748058	0,03157	-0,65656
EPCAM	-2,14242	0,05548	-5,68029	1,34E-08	0,000125	9,152875
EPHA3	1,25322	0,044324	3,322714	0,000891466	0,035072	-0,80931
ERAP1	1,716704	0,018142	4,551568	5,32E-06	0,003103	3,732334
ERAP2	1,389418	0,034149	3,683823	0,000229763	0,017575	0,378215
ERP44	1,184636	0,008596	3,140874	0,001684448	0,049298	-1,36096
ESPN	-1,24007	0,044097	-3,28785	0,001009556	0,037523	-0,91748
ETS1	1,189589	0,00453	3,154006	0,00161046	0,048277	-1,32216
EVI2A	1,210317	0,024004	3,208965	0,001332142	0,044135	-1,15803
F11R	-1,19684	0,025903	-3,17324	0,001507457	0,047117	-1,26503
FA2H	-1,64877	0,046739	-4,37146	1,23E-05	0,004775	2,978019
FAM114A2	1,466833	0,000402	3,889076	0,000100627	0,012171	1,107763
FAM126A	1,288813	-0,01998	3,417084	0,000632959	0,028023	-0,51078
FAM131C	-1,41908	0,043736	-3,76246	0,000168251	0,014698	0,653047
FAM155A	-1,34653	0,039701	-3,5701	0,00035684	0,021488	-0,00896
FAM177A1	-1,47338	0,008266	-3,90644	9,37E-05	0,01189	1,171314
FAM208A	1,182221	-0,03735	3,134472	0,001721643	0,049952	-1,37982
FAM83H	-1,45707	0,041858	-3,8632	0,000111911	0,012454	1,013616
FBXL12	1,560229	-0,03888	4,1367	3,52E-05	0,008178	2,04055
FBXO21	-1,41723	0,022219	-3,75757	0,000171573	0,014908	0,635782
FBXO3	-1,46118	0,037719	-3,87407	0,000107031	0,012241	1,053102
FBXO38	1,485787	0,016914	3,93933	8,17E-05	0,011064	1,292411
FBXO7	1,374136	-0,00317	3,643305	0,000269161	0,019097	0,238873
FBXW8	1,298161	0,045769	3,441868	0,000577713	0,026014	-0,431
FCER1A	1,509019	0,041754	4,000924	6,31E-05	0,009536	1,521962
FCGR2A	1,553237	0,025036	4,118163	3,82E-05	0,008618	1,968727
FCGR2B	1,384219	0,015944	3,670036	0,000242517	0,018243	0,330631
FCRLA	1,347808	0,028844	3,573501	0,000352241	0,021435	0,00243
FCRLB	1,778278	0,030898	4,714824	2,42E-06	0,002329	4,442369
FER	1,316533	0,02937	3,49058	0,000481975	0,024626	-0,2725
FGL1	-1,25358	0,026295	-3,32368	0,000888384	0,035035	-0,8063
FIGNL1	-1,29979	-0,02717	-3,44618	0,000568566	0,025952	-0,41705
FKBP3	-1,37593	-0,01683	-3,64807	0,000264217	0,018829	0,255186
FNBP1L	-1,56717	0,042941	-4,15511	3,25E-05	0,008127	2,11221
FOXA1	-1,48406	0,05192	-3,93475	8,33E-05	0,011092	1,275485
FUT11	1,454277	0,025986	3,855786	0,000115359	0,01249	0,986751
FZR1_ENST	-1,40522	0,071424	-3,72573	0,000194754	0,016063	0,523946
GADD45G	-1,57694	0,077265	-4,181	2,90E-05	0,007485	2,213508
GAL	-1,29922	0,050929	-3,44469	0,000571721	0,025952	-0,42188
GBP4	1,309565	0,036398	3,472105	0,000516396	0,025243	-0,33288
GCLC	-1,45684	0,046791	-3,86258	0,000112197	0,012454	1,011356
GDF11	1,258102	0,046646	3,335659	0,000850977	0,034054	-0,76886
GEMIN7	1,209089	0,013684	3,205709	0,001347305	0,044391	-1,16783
GGA1	1,334477	0,012807	3,538155	0,000402935	0,022546	-0,11555
GIT2	1,550975	0,022446	4,112165	3,92E-05	0,008696	1,945559
GLB1L2	-1,6454	0,06354	-4,36252	1,29E-05	0,004858	2,941364
GMFB	-1,69319	0,008204	-4,48923	7,15E-06	0,003124	3,467794
GNA11	-1,34516	-0,01798	-3,56649	0,000361795	0,021502	-0,02106
GNAI2	1,214489	-0,03922	3,220025	0,001281798	0,043209	-1,12466
GNG4	-1,44428	0,050485	-3,82929	0,000128515	0,01317	0,891173
GNG7	1,562086	0,028638	4,141623	3,45E-05	0,008178	2,059677
GNRH1	1,369289	0,045841	3,630453	0,000282925	0,019372	0,194998
GPD2	-1,38915	-0,0168	-3,68312	0,000230396	0,017575	0,375791
GRAP2	1,191992	0,013633	3,160377	0,001575652	0,047942	-1,30328
GRTP1	-1,54098	0,045521	-4,08567	4,40E-05	0,008963	1,843604
GSTO2	-1,25438	0,047998	-3,3258	0,00088166	0,034855	-0,79969
GYPC	2,053167	0,007523	5,443647	5,22E-08	0,00017	7,917347
H1F0	-1,50626	0,044706	-3,9936	6,51E-05	0,009536	1,494489
HACD3_EN	-1,34122	-0,00326	-3,55602	0,000376513	0,021771	-0,05606
HAPLN3	1,47064	0,016264	3,89917	9,65E-05	0,012101	1,144662
HAUS6	1,563184	-0,02028	4,144536	3,41E-05	0,008178	2,071006
HELZ	1,201387	0,002425	3,185286	0,001446112	0,045802	-1,22909
HLA-DPA1	1,317239	0,020072	3,492449	0,000478614	0,024596	-0,26637
HLA-DRA	1,607009	0,027616	4,260731	2,04E-05	0,006247	2,529401
HLA-E	1,419289	-0,0216	3,76302	0,000167874	0,014698	0,655028
HLA-F	1,241898	0,019391	3,292695	0,000992322	0,037378	-0,90252
HMGB3	-1,32364	0,029257	-3,50941	0,000449097	0,023691	-0,21062
HOOK1	-1,76849	0,05936	-4,68887	2,75E-06	0,002349	4,327823
HOXA11	-1,3672	0,031631	-3,62492	0,000289049	0,019545	0,176166
HPSE	-1,49391	0,058029	-3,96086	7,47E-05	0,010543	1,372239
HYLS1	-1,3391	-0,0257	-3,5504	0,000384649	0,022006	-0,07481

ICA1	-1,45558	0,058824	-3,85923	0,000113746	0,012487	0,999218
IFFO1	1,315188	0,01097	3,487013	0,00048845	0,024667	-0,28418
IFI16	1,799783	-0,00577	4,771839	1,83E-06	0,002289	4,696234
IGFBP1	-1,27985	0,050351	-3,39331	0,000690531	0,02984	-0,58677
IGFBPL1	-1,44286	0,031662	-3,82552	0,000130498	0,01317	0,877629
IKBIP	1,433484	0,003179	3,800656	0,000144314	0,013875	0,788637
IL17RB	-1,34336	0,040227	-3,56171	0,000368451	0,021536	-0,03706
IL1RAP	1,208484	0,010578	3,204104	0,001354837	0,044391	-1,17266
IL21R	1,206058	0,038927	3,197671	0,001385427	0,044931	-1,19199
IL4I1	1,225241	0,01774	3,248532	0,001160023	0,040797	-1,03811
IL7	1,516428	0,052353	4,020568	5,81E-05	0,009536	1,595919
INHBB	-1,43911	0,052353	-3,81558	0,000135864	0,013581	0,841985
IRAK4	1,506533	0,025707	3,994335	6,49E-05	0,009536	1,497234
ISCA2	-1,20458	-0,01753	-3,19375	0,001404404	0,045186	-1,20377
ITGA4	1,231851	0,027389	3,266057	0,001090565	0,039198	-0,98452
ITGAM	1,537444	0,019422	4,07629	4,58E-05	0,008963	1,807678
ITPR1	1,190018	0,023179	3,155143	0,001604196	0,048277	-1,3188
JAG2	-1,31356	0,056904	-3,4827	0,00049639	0,024828	-0,2983
JAK2	1,329148	0,036336	3,524026	0,000425044	0,023253	-0,16238
JPT2_ENST	-1,35517	0,029856	-3,59302	0,000326869	0,020735	0,068084
KCND1	1,412286	0,017802	3,744451	0,000180789	0,015299	0,589598
KCNQ5	1,324289	0,028916	3,511143	0,000446186	0,023691	-0,20492
KCTD3	-1,21187	0,008762	-3,21309	0,001313142	0,043901	-1,14559
KDF1_ENS	-1,23379	0,057327	-3,2712	0,001070932	0,039102	-0,96875
KIAA0319	-1,42872	0,032652	-3,78802	0,000151852	0,014131	0,743639
KLF3	-1,32981	0,005263	-3,52579	0,000422229	0,023177	-0,15656
KLF4	-1,38566	0,051414	-3,67385	0,000238921	0,018056	0,343786
KLF5	-1,20846	0,045697	-3,20404	0,00135513	0,044391	-1,17285
KLRG1	1,195599	0,024469	3,169942	0,001524696	0,047187	-1,27486
KRBOX4	1,286475	-0,00994	3,410884	0,000647528	0,028512	-0,53065
KRT19	-1,69375	0,028514	-4,49072	7,10E-06	0,003124	3,474097
KRT8	-2,11263	0,053664	-5,60131	2,13E-08	0,000125	8,734673
L3MBTL2	1,261651	-0,00189	3,345068	0,000822627	0,033446	-0,73935
LAMP2	1,229395	-0,01536	3,259546	0,001115911	0,039673	-1,00446
LAPTM5	1,210188	0,029226	3,208622	0,001333731	0,044135	-1,15906
LCAT	1,425905	-0,00312	3,780561	0,000156476	0,014203	0,717132
LDLRAD4	1,204577	0,025201	3,193745	0,001404404	0,045186	-1,20377
LENG8	1,319777	0,019443	3,499179	0,000466695	0,024382	-0,24429
LGI2	-1,6361	0,041878	-4,33786	1,44E-05	0,005195	2,840664
LGSN	-1,25883	0,053189	-3,33759	0,000845084	0,033987	-0,76281
LIFR	-1,46537	0,030392	-3,8852	0,000102247	0,012171	1,093609
LILRA5	-1,18799	0,040516	-3,14977	0,001634	0,048679	-1,3347
LIPH	-1,19747	0,047688	-3,17491	0,001498825	0,047013	-1,26006
LLGL2	-1,41328	0,06838	-3,7471	0,000178892	0,015219	0,598909
LPXN	1,477573	0,022477	3,917552	8,95E-05	0,011561	1,212102
LRRC1	-1,36927	0,023973	-3,63041	0,000282976	0,019372	0,194839
LRRC20	-1,19172	0,030258	-3,15965	0,001579617	0,047973	-1,30545
LRRFIP2	1,467362	0,00582	3,890478	0,000100047	0,012171	1,112882
LRRK1	1,382714	0,045686	3,666048	0,000246328	0,018372	0,316899
LRRK2	1,360488	0,028999	3,607118	0,000309618	0,020533	0,115727
LRRN4CL	1,532556	0,015893	4,063329	4,84E-05	0,009064	1,758164
LRTOMT	-1,40908	0,074128	-3,73596	0,000187	0,015743	0,55979
LSM4	-1,40054	-0,00861	-3,71331	0,000204566	0,016293	0,480597
MAGI3	-1,7038	0,02838	-4,51736	6,26E-06	0,003124	3,586722
MALT1	1,5098	0,016615	4,002996	6,25E-05	0,009536	1,529745
MAP1LC3B	1,349183	0,006945	3,577146	0,000347367	0,021379	0,014664
MAP3K3	1,345029	0,028246	3,566132	0,00036229	0,021502	-0,02226
MAP4	1,197556	-0,01057	3,17513	0,001497699	0,047013	-1,25941
MAP4K3	-1,49226	0,003571	-3,95648	7,61E-05	0,010543	1,355972
MAP7D3	1,350447	-0,02842	3,580495	0,000342945	0,021268	0,025915
MARVELD2	-1,3427	0,048844	-3,55996	0,000370907	0,021536	-0,04289
MARVELD3	-1,39635	0,069835	-3,7022	0,000213735	0,016777	0,441939
MBIP	-1,43415	0,011785	-3,80242	0,000143292	0,013875	0,794919
MDGA1	1,218314	0,045067	3,230166	0,001237186	0,042448	-1,09395
MDK	-1,5197	0,035325	-4,02924	5,60E-05	0,009536	1,628702
MFAP3	1,34879	0,027998	3,576102	0,000348756	0,021383	0,011159
MGAT4A	-1,49514	0,048617	-3,96411	7,37E-05	0,010543	1,384349
MGST3	-1,38265	-0,01128	-3,66588	0,000246493	0,018372	0,31631
MLXIPL	-1,32573	0,051434	-3,51497	0,000439797	0,023584	-0,19229
MMAA	1,21158	0,025882	3,212314	0,001316707	0,04393	-1,14794
MOB3A	1,23161	0,005325	3,265418	0,001093027	0,039198	-0,98648
MOSPD2	1,251968	-0,01239	3,319396	0,000902127	0,035168	-0,81966
MR1	1,721498	0,034933	4,56428	5,01E-06	0,003103	3,786721
MROH1	-1,20692	0,049174	-3,19996	0,001374467	0,044844	-1,18511
MRPL49	-1,47036	0,017152	-3,89842	9,68E-05	0,012101	1,141926

MSN	1,301134	-0,02021	3,449751	0,000561106	0,025952	-0,4055
MSR1	1,771845	0,046987	4,697766	2,63E-06	0,002349	4,367011
MSX2	-1,5374	0,040237	-4,07617	4,58E-05	0,008963	1,807202
MT3	-1,21539	0,018596	-3,22241	0,001271182	0,043119	-1,11745
MUC7	1,407156	0,02808	3,730852	0,000190834	0,015901	0,541883
MYO5C	-1,35469	0,062157	-3,59174	0,000328476	0,020735	0,063777
MYO6	-1,28501	0,038803	-3,40701	0,000656801	0,028727	-0,54307
MYO7A	-1,31046	0,055955	-3,47449	0,00051183	0,025201	-0,3251
MYOC	1,427391	0,022848	3,784502	0,000154017	0,014203	0,731126
MYOZ1	-1,30657	0,02257	-3,46418	0,000531861	0,025506	-0,35869
NAA30	-1,30684	0,029102	-3,46488	0,000530476	0,025506	-0,35641
NAP1L1	1,21923	0,003467	3,232596	0,001226711	0,042228	-1,08658
NDUFB9	-1,30024	0,018865	-3,44738	0,000566047	0,025952	-0,41317
NFKB1	1,299606	-0,04165	3,4457	0,000569583	0,025952	-0,41861
NGLY1	1,308678	-0,00334	3,469752	0,00052094	0,025342	-0,34054
NINL	-1,45445	0,033922	-3,85625	0,000115139	0,01249	0,988443
NISCH	1,327709	-0,02533	3,520209	0,000431208	0,023354	-0,175
NLRC5	1,377697	0,016326	3,652745	0,000259453	0,018736	0,2712
NMNAT2	-1,69859	0,047245	-4,50354	6,68E-06	0,003124	3,528219
NOVA1	-1,46533	0,033302	-3,88509	0,000102293	0,012171	1,093211
NR1H3	-1,33564	0,048751	-3,54124	0,000398254	0,02239	-0,1053
NRBF2	1,526569	0,000712	4,047455	5,18E-05	0,009348	1,697735
NUP62CL	-1,25151	0,035769	-3,31818	0,000906061	0,035219	-0,82344
OPLAH	-1,22418	0,032941	-3,24571	0,001171573	0,040977	-1,0467
OR13C4	1,465035	-0,00541	3,884309	0,000102622	0,012171	1,090371
OR2H1	1,208002	0,017389	3,202827	0,00136086	0,044489	-1,1765
OR4K2	1,270335	-0,00262	3,368092	0,000756906	0,031678	-0,66681
OR6T1	1,226645	0,001187	3,252255	0,001144934	0,040353	-1,02675
OR8K3	1,328337	0,00324	3,521876	0,000428506	0,023286	-0,16949
ORAI2	1,746486	0,02742	4,630532	3,65E-06	0,002648	4,072642
OSTM1	1,340241	0,021569	3,553436	0,000380234	0,021831	-0,06469
OTULINL	-1,44841	0,034603	-3,84024	0,000122915	0,012802	0,930594
OVOL2	-1,50731	0,06451	-3,99639	6,43E-05	0,009536	1,504946
OXSR1	1,261293	-0,01794	3,344118	0,00082545	0,033446	-0,74234
P2RX7	1,3001	0,026285	3,447009	0,000566831	0,025952	-0,41438
P2RY11	1,430752	0,02194	3,793412	0,000148592	0,013956	0,762818
P2RY2	-1,24648	0,0271	-3,30485	0,000950288	0,036438	-0,86489
PACRGL	-1,21289	0,011218	-3,21579	0,001300873	0,043671	-1,13745
PACS2	-1,30729	0,035635	-3,46606	0,000528146	0,025506	-0,35256
PAIP1	-1,18588	0,014665	-3,14416	0,001665641	0,049143	-1,35127
PAIP2B	-1,51712	0,041084	-4,02239	5,76E-05	0,009536	1,602799
PAK4	-1,25203	0,046749	-3,31957	0,000901574	0,035168	-0,81912
PAQR5	-1,18447	0,03967	-3,14044	0,001686958	0,049298	-1,36225
PAQR6	1,315106	0,040619	3,486795	0,000488848	0,024667	-0,2849
PARP2	-1,70967	-0,03127	-4,53292	5,82E-06	0,003103	3,652827
PAXX	-1,28487	-0,0004	-3,40663	0,000657701	0,028727	-0,54426
PBX1	-1,26484	0,038607	-3,35353	0,00079789	0,032904	-0,71276
PCDH18	1,296968	0,055088	3,438706	0,000584503	0,026163	-0,44121
PCDH8	-1,19062	0,054076	-3,15675	0,001595396	0,048182	-1,31404
PDPR	1,214912	0,010175	3,221146	0,001276792	0,043205	-1,12126
PEX10	-1,23986	0,022281	-3,28731	0,001011513	0,037523	-0,91916
PGP	-1,29789	-0,00465	-3,44115	0,000579245	0,026014	-0,43331
PIK3CA	1,356827	-0,00169	3,597413	0,000321399	0,020725	0,08291
PIKFYVE	1,229166	0,012828	3,258938	0,001118303	0,039673	-1,00632
PILRA	1,356434	0,03485	3,596369	0,000322691	0,020725	0,079386
PIMREG_El	-1,33105	0,002817	-3,52906	0,000417044	0,023127	-0,14573
PKP4	-1,3287	-0,00876	-3,52283	0,000426973	0,02328	-0,16635
PLA2G15	1,403684	0,021682	3,721646	0,000197929	0,016108	0,50968
PLBD1	-1,21239	0,053932	-3,21446	0,001306888	0,043782	-1,14145
PLCD4	1,350823	0,013137	3,581492	0,000341639	0,021268	0,029267
PLEK	1,204806	0,034417	3,194353	0,001401451	0,045186	-1,20194
PLEK2	-1,59193	0,054623	-4,22076	2,43E-05	0,007064	2,370277
PLEKHA1	-1,25729	0,028782	-3,33349	0,000857629	0,034154	-0,77563
PLEKHA2	1,438854	0,0129	3,814894	0,000136242	0,013581	0,83953
PLEKHA6	-1,31697	0,06227	-3,49175	0,000479871	0,024596	-0,26867
PLS1	-1,25661	0,004056	-3,3317	0,000863168	0,03429	-0,78124
PML	1,614753	0,029123	4,281262	1,86E-05	0,006039	2,611717
PNKP	-1,26706	-0,01254	-3,35941	0,000781079	0,032458	-0,69421
PNLIP	-1,30938	0,020227	-3,47161	0,000517356	0,025243	-0,3345
PNPLA6	1,450388	0,014303	3,845473	0,00012032	0,012695	0,949475
POFUT2	1,299689	0,027131	3,445918	0,000569124	0,025952	-0,4179
POMC	-1,33427	0,030021	-3,53759	0,000403792	0,022546	-0,11741
PPARG	-1,36651	0,055738	-3,62308	0,000291111	0,019545	0,169913
PPHLN1	1,332667	0,026151	3,533357	0,00041032	0,022832	-0,13148
PPP1R16A	-1,369	0,062219	-3,62969	0,000283763	0,019372	0,192397

PPP1R18	1,48423	-0,02711	3,935202	8,31E-05	0,011092	1,277154
PPP3CC	1,246416	0,005325	3,304675	0,00095087	0,036438	-0,86542
PRKCZ	-1,91119	0,035686	-5,06721	4,04E-07	0,001093	6,060258
PRKG2	-1,20649	0,018204	-3,19881	0,001379974	0,044931	-1,18858
PRMT5	-1,68934	-0,00257	-4,47902	7,50E-06	0,003124	3,424844
PRR15	-1,33767	0,042755	-3,54663	0,000390195	0,02209	-0,08737
PSEN1	-1,28697	0,019205	-3,41221	0,000644391	0,028451	-0,52641
PSMA3	-1,64961	-0,01434	-4,37369	1,22E-05	0,004775	2,987162
PSMA8	1,233384	0,02711	3,270123	0,00107501	0,039163	-0,97205
PSMB11	1,231392	-0,02285	3,264842	0,001095253	0,039198	-0,98824
PSMB5	-1,40056	-0,00301	-3,71336	0,000204528	0,016293	0,48076
PSMB8	1,25785	-0,02951	3,334989	0,00085303	0,034054	-0,77095
PSMB9	1,514248	-0,00657	4,014789	5,95E-05	0,009536	1,574122
PSMC3	-1,35643	-0,01965	-3,59635	0,00032271	0,020725	0,079333
PTGES2	-1,57848	0,002683	-4,1851	2,85E-05	0,00747	2,229596
PTK2	-1,29155	0,02645	-3,42434	0,000616289	0,027459	-0,48747
PTPN22	1,270129	0,022559	3,367547	0,000758404	0,031678	-0,66853
PUF60	-1,41598	0,009835	-3,75423	0,000173873	0,014948	0,624029
PWWP2B	-1,29103	0,049814	-3,42297	0,000619405	0,027498	-0,49188
PXK	1,342644	0,017792	3,559808	0,000371127	0,021536	-0,04341
QKI	1,343032	-0,03724	3,560836	0,000369677	0,021536	-0,03998
QPCTL	1,186316	0,00065	3,145329	0,001659003	0,049143	-1,34782
RAB10	-1,31351	0,022312	-3,48257	0,000496621	0,024828	-0,29871
RAB15	-1,75279	0,020433	-4,64725	3,36E-06	0,002603	4,145429
RAB3GAP2	1,272203	-0,00638	3,373046	0,000743418	0,031538	-0,65113
RAB40C	-1,41328	0,042704	-3,74708	0,000178903	0,015219	0,598854
RAB8B	1,190658	-0,01454	3,156841	0,001594885	0,048182	-1,31376
RALGPS1	-1,57509	0,052962	-4,17611	2,97E-05	0,007528	2,194322
RAP1GAP	-1,41979	0,065604	-3,76434	0,000166987	0,014698	0,659706
RAP2C	1,527714	-0,01089	4,050493	5,11E-05	0,009331	1,70928
RB1	1,354236	-0,00308	3,590543	0,000329991	0,020735	0,059733
RBM43	1,306199	0,028308	3,463179	0,000533835	0,025506	-0,36193
RBM47	-1,3161	0,034169	-3,48944	0,00048403	0,024654	-0,27622
RCBTB2	1,535576	0,043282	4,071336	4,67E-05	0,009031	1,788735
RECK	1,460276	0,013746	3,871691	0,000108083	0,012241	1,044437
RECQL	1,282098	-0,02547	3,399279	0,00067564	0,029352	-0,56775
RELB	1,343784	0,00999	3,56283	0,00036688	0,021536	-0,03331
RENBP	1,433954	0,022343	3,801902	0,00014359	0,013875	0,793084
RFTN1	1,753484	0,014995	4,649085	3,33E-06	0,002603	4,153449
RFX5	1,310629	-0,01399	3,474924	0,000510999	0,025201	-0,32368
RHOG	1,318061	-0,02346	3,49463	0,00047472	0,024544	-0,25922
RHOQ	1,285076	-0,02712	3,407176	0,000656388	0,028727	-0,54252
RHOU	-2,09646	0,057626	-5,55842	2,72E-08	0,000125	8,510043
RHPN2	-1,20241	0,027172	-3,18801	0,001432547	0,045729	-1,22094
RILPL2	1,508978	0,00549	4,000815	6,31E-05	0,009536	1,521552
RIMKLA	-1,18655	0,055573	-3,14594	0,001655561	0,049143	-1,34602
RIPPLY3	-1,81247	0,033251	-4,80549	1,54E-06	0,00228	4,847487
RPL22L1	1,27671	0,043633	3,384994	0,000711801	0,030596	-0,61324
RPL3	1,354924	-0,01079	3,592365	0,000327691	0,020735	0,065878
RPP25	-1,34458	0,020144	-3,56495	0,000363929	0,021502	-0,02622
RSU1	1,378566	-0,01474	3,65505	0,000257132	0,018664	0,279108
RTL3	1,58309	0,029587	4,197314	2,70E-05	0,007314	2,277646
RTN3	-1,73195	-0,01198	-4,59199	4,39E-06	0,002853	3,905818
RWDD3	1,196492	0,019701	3,17231	0,001512318	0,047117	-1,26781
S100PBP	1,245593	-0,01979	3,302494	0,000958295	0,03655	-0,87219
SAC3D1	-1,21032	0,017637	-3,20896	0,001332142	0,044135	-1,15803
SACS	1,524436	-0,04017	4,0418	5,30E-05	0,009452	1,676265
SAMD9	1,202591	0,013653	3,18848	0,001430233	0,045729	-1,21954
SAMD9L	1,42611	0,036109	3,781106	0,000156134	0,014203	0,719067
SAPCD2	-1,36666	-0,00528	-3,62349	0,000290656	0,019545	0,17129
SCAF11	1,266616	0,008235	3,358231	0,000784432	0,032514	-0,69794
SCNN1A	-1,39229	0,05323	-3,69142	0,000223002	0,017337	0,40453
SCRIB	-1,71316	0,018607	-4,54217	5,57E-06	0,003103	3,692241
SEC31B	1,324318	0,027286	3,511221	0,000446055	0,023691	-0,20467
SENP8	-1,33813	0,028689	-3,54784	0,000388399	0,022065	-0,08333
SERPINA9	1,376774	0,029009	3,650299	0,000261936	0,018829	0,262817
SFTA2	-1,24477	0,034035	-3,30031	0,000965773	0,036749	-0,87895
SH3BGRL	1,86876	-0,01914	4,954722	7,24E-07	0,001471	5,531135
SH3BGRL2	-1,49397	0,05678	-3,96103	7,46E-05	0,010543	1,372876
SHANK2	-1,37948	0,049845	-3,65748	0,000254707	0,018664	0,287449
SHQ1	1,461081	-0,0098	3,873825	0,000107141	0,012241	1,052196
SHROOM4	1,344918	0,016863	3,565836	0,000362699	0,021502	-0,02325
SIGLEC10	1,22792	0,022239	3,255636	0,001131391	0,039982	-1,01642
SLC12A7	-1,20083	0,023963	-3,18382	0,001453447	0,045945	-1,23347
SLC25A10	-1,33682	0,013622	-3,54437	0,000393553	0,022203	-0,09489

SLC25A17	1,361663	0,003075	3,610233	0,000305923	0,020371	0,126281
SLC25A21	-1,22384	0,061723	-3,24482	0,001175232	0,040977	-1,0494
SLC27A2	-1,40175	0,026171	-3,7165	0,000201998	0,016248	0,491733
SLC2A4RG	-1,33039	0,0358	-3,52731	0,000419802	0,023177	-0,15151
SLC35B1	-1,37036	0,034623	-3,63329	0,000279833	0,019372	0,204664
SLC35D1	1,5801	0,005397	4,189385	2,80E-05	0,00745	2,246434
SLC36A4	-1,22386	-0,00716	-3,24487	0,001175039	0,040977	-1,04926
SLC39A4	-1,48912	0,015851	-3,94818	7,87E-05	0,010752	1,325167
SLC39A5	-1,2058	0,01257	-3,19699	0,001388723	0,044948	-1,19405
SLC40A1	-1,56099	0,050454	-4,13871	3,49E-05	0,008178	2,048355
SLC45A4	-1,4536	0,055769	-3,85399	0,000116207	0,01249	0,980269
SLC9A9	1,371081	0,037162	3,635204	0,000277761	0,019372	0,2112
SLIRP	-1,40352	-0,01025	-3,72121	0,000198272	0,016108	0,508156
SMPDL3A	-1,3626	0,051651	-3,61273	0,000302996	0,02026	0,134731
SMU1	1,509389	0,00258	4,001906	6,28E-05	0,009536	1,525648
SNAP23	1,238578	-0,02775	3,283894	0,001023837	0,037808	-0,92969
SP110	1,329871	0,02032	3,525942	0,000421981	0,023177	-0,15604
SPARC	1,194224	0,025098	3,166297	0,001543934	0,047421	-1,2857
SPINT2	-1,30942	0,035139	-3,47173	0,000517116	0,025243	-0,33409
SPTA1	1,507327	0,031765	3,996438	6,43E-05	0,009536	1,505122
ST3GAL2	1,217568	0,024995	3,228188	0,001245775	0,042613	-1,09995
STAT2	1,517679	0,04388	4,023886	5,72E-05	0,009536	1,608447
STAT5A	1,305922	0,018596	3,462447	0,00053529	0,025506	-0,36431
STAT5B	1,198226	-0,00142	3,176905	0,001488559	0,046963	-1,25412
STK10	1,193749	0,025356	3,165035	0,001550645	0,047538	-1,28945
STK17A	1,475141	-0,01425	3,911103	9,19E-05	0,011754	1,188405
STRN3	-1,47737	0,000279	-3,91701	8,97E-05	0,011561	1,210097
STYK1	-1,69128	0,063633	-4,48416	7,32E-06	0,003124	3,446471
SUFU	1,391334	0,027853	3,688901	0,000225226	0,017384	0,395789
SULT2B1	-1,2158	0,052611	-3,22351	0,001266283	0,043119	-1,1141
SVIP	-1,32373	-0,00232	-3,50965	0,000448703	0,023691	-0,20985
SWAP70	1,22245	-0,01663	3,241133	0,00119056	0,041422	-1,06064
SYBU	-1,31139	0,062549	-3,47693	0,000507184	0,025201	-0,31713
SYT17	-1,31098	0,056151	-3,47586	0,000509221	0,025201	-0,32063
TAB1	1,270564	0,017152	3,368699	0,00075524	0,031678	-0,66489
TAB3	1,298108	0,030093	3,441728	0,000578013	0,026014	-0,43145
TACC2	-1,50947	0,048978	-4,00211	6,28E-05	0,009536	1,526409
TAPBP	1,19631	0,027719	3,171827	0,001514835	0,047117	-1,26925
TATDN1	-1,24071	0,006904	-3,28955	0,001003485	0,037482	-0,91224
TBC1D10C	1,241293	0,028865	3,291091	0,000997999	0,037449	-0,90748
TBC1D8	-1,44924	-0,00162	-3,84244	0,00012182	0,01277	0,938514
TCTE1	1,276798	0,030568	3,385227	0,000711195	0,030596	-0,61249
TESPA1	1,741022	0,011723	4,616045	3,91E-06	0,002648	4,009768
TGFB1	1,351851	0,038813	3,584218	0,00033809	0,021128	0,038435
TGFB2	1,248972	0,011176	3,311451	0,000928137	0,035888	-0,84438
THEM6	-1,30458	0,052466	-3,45888	0,00054243	0,025695	-0,3759
TIGD3	-1,23652	0,053519	-3,27843	0,00104388	0,038373	-0,94653
TIMM9	-1,2707	-0,01018	-3,36906	0,000754258	0,031678	-0,66375
TJP1	-1,27636	0,005996	-3,38407	0,000714188	0,030618	-0,61616
TJP3	-1,24079	0,037874	-3,28977	0,001002707	0,037482	-0,91157
TLE2	-1,35753	0,050124	-3,59928	0,000319098	0,020725	0,089224
TLN1	1,325799	-0,001	3,515146	0,000439513	0,023584	-0,19172
TMC4	-1,19618	0,071765	-3,17148	0,001516623	0,047117	-1,27027
TMC5	-1,24174	0,068514	-3,29227	0,000993807	0,037378	-0,90382
TMEM259	1,259283	0,004107	3,33879	0,000841444	0,033925	-0,75905
TMEM45B	-1,65471	0,065562	-4,38719	1,15E-05	0,004664	3,042698
TONSL	-1,23203	0,037296	-3,26654	0,001088706	0,039198	-0,98304
TOX3	-1,53924	0,047595	-4,08104	4,48E-05	0,008963	1,825869
TOX4	-1,29935	0,017007	-3,44501	0,000571029	0,025952	-0,42083
TP53RK	1,422479	0,011837	3,771479	0,000162283	0,014649	0,684941
TRIM22	1,780899	0,030114	4,721771	2,34E-06	0,002329	4,473141
TRIM38	1,544113	-0,00524	4,093971	4,24E-05	0,008963	1,87548
TRIM5	1,594824	0,008937	4,228423	2,35E-05	0,007064	2,400671
TSC22D4	1,407321	0,037967	3,731288	0,000190504	0,015901	0,543411
TSHR	1,229224	0,035304	3,259094	0,001117689	0,039673	-1,00585
TTC39A	-1,467	0,049051	-3,88951	0,000100446	0,012171	1,109355
TUBB4B	-1,42851	-0,05437	-3,78746	0,000152195	0,014131	0,741645
TYK2	1,607938	-0,01735	4,263192	2,02E-05	0,006247	2,539248
UBA7	1,283649	0,017451	3,403391	0,000665551	0,028992	-0,55462
UBE2L6	1,319289	-0,02751	3,497886	0,000468963	0,024422	-0,24853
UCN	1,406058	0,033313	3,727939	0,000193053	0,016004	0,531685
UGDH	-1,20353	0,016171	-3,19097	0,001417952	0,045442	-1,21208
UNC5C	1,183143	0,041445	3,136917	0,001707346	0,049715	-1,37262
UQCC2_EN	-1,20627	-0,03829	-3,19825	0,001382661	0,044931	-1,19026
USP4	1,310828	-0,04471	3,475454	0,000509991	0,025201	-0,32195

VIM	1,187867	-0,04434	3,149442	0,00163583	0,048679	-1,33567
VSIG10	-1,48093	0,066078	-3,92646	8,62E-05	0,011296	1,244907
WDR11	1,534642	-0,00545	4,068859	4,72E-05	0,009031	1,779271
WDR34	-1,88588	0,014345	-5,00012	5,73E-07	0,00133	5,743231
WDR36	1,37151	-0,02449	3,636341	0,000276539	0,019372	0,215082
WDR41	1,460217	-0,00852	3,871535	0,000108153	0,012241	1,043871
WDR91	1,852474	0,032178	4,91154	9,04E-07	0,00162	5,33117
WDYHV1	-1,24411	0,013488	-3,29857	0,000971795	0,036892	-0,88435
WIPF1	1,359148	0,012879	3,603566	0,000313882	0,020584	0,103707
WIPI1	1,346463	0,004386	3,569933	0,000357073	0,021488	-0,00953
WNK2	-1,71119	0,028163	-4,53694	5,71E-06	0,003103	3,669937
WSB2	-1,19452	0,007245	-3,16708	0,001539805	0,047384	-1,28339
XAF1	1,189389	0,019732	3,153476	0,001613386	0,048277	-1,32373
XIRP1	1,196375	0,011022	3,171998	0,001513941	0,047117	-1,26874
XPO1	-1,18865	-0,01365	-3,15153	0,001624182	0,048511	-1,32949
XRN1	1,216381	0,009835	3,225041	0,00125955	0,042994	-1,10948
YPEL4	1,315699	0,036945	3,488368	0,00048598	0,024667	-0,27974
YTHDC2	1,340323	0,00453	3,553655	0,000379919	0,021831	-0,06396
ZBTB33	1,182591	-0,02517	3,135453	0,001715893	0,049874	-1,37693
ZCCHC24	1,24322	0,026068	3,2962	0,000980024	0,037035	-0,89168
ZCCHC7	1,379806	0,013674	3,658337	0,000253858	0,018664	0,290391
ZCCHC9	1,261645	-0,01825	3,345052	0,000822673	0,033446	-0,7394
ZEB2	1,209201	0,017079	3,206005	0,00134592	0,044391	-1,16694
ZFC3H1	1,563572	0,007162	4,145564	3,39E-05	0,008178	2,075007
ZFP42	-1,20184	0,03193	-3,1865	0,001440051	0,045789	-1,22546
ZMAT3	1,539495	0,029432	4,081726	4,47E-05	0,008963	1,828495
ZNF106	1,248772	-0,02794	3,310921	0,000929896	0,035888	-0,84603
ZNF165	-1,50959	0,06386	-4,00244	6,27E-05	0,009536	1,527638
ZNF219	-1,1836	0,026533	-3,13812	0,001700375	0,049601	-1,36909
ZNF330	1,184518	-0,01635	3,140562	0,001686241	0,049298	-1,36188
ZNF468	-1,36968	0,044572	-3,63148	0,0002818	0,019372	0,198502
ZNF488	-1,41974	0,03065	-3,76422	0,000167071	0,014698	0,659265
ZNF490	1,19312	0,010423	3,163368	0,001559552	0,047721	-1,2944
ZNF654	1,742785	0,005273	4,620718	3,82E-06	0,002648	4,030028
ZNF70	1,402955	0,000547	3,719714	0,000199449	0,016123	0,502933
ZNF805	1,261345	0,019257	3,344258	0,000825032	0,033446	-0,7419
ZNRF3	-1,30649	0,038751	-3,46394	0,000532323	0,025506	-0,35945
ZSWIM3	1,275746	0,005181	3,382439	0,000718454	0,03072	-0,62135

insensitive genes	sensitive genes
ABCC3	ANKRD27
ABHD12	ASAH1
ACTR8	BCAP29
ADCK5	BCL9
ALDH18A1	C5orf22
ANAPC11	COPA
ANKRD40	EPDR1
ASH1L	FMO5
ATAD2	IGFLR1
ATP9A	IMMP2L
ATXN7	KDR
AZIN1	KLHL20
BRAT1	OR13A1
BRIX1	PEX11B
BRMS1L	PKN1
C18orf21	PLOD3
C3orf38	POFUT2
C5orf22	RALGAPB
CATSPER1	RPS6KC1
CBFA2T2	SAMSN1
CCDC66	SLC2A10
CCNH	TMEM248
CCT5	TRIM45
CDC37L1	ZNF248
CDC42EP2	ZNF337
CENPX_ENST00000580435	ZNF33A
CHCHD1	ZNHIT1
COA3	ATP5MPL_ENST00000555030
COX4I1	DDX3Y
CTNNA1	EIF1AY
CTNND1	FKBP3
CUL2	GINS1
DDX3Y	ID1
DERL1	KDM5D
DGAT1	KLHL9
DHX29	RPS29
DNAJB12	RPS4Y1
DNAJC21	RTRAF
DPY19L1	USP9Y
DROSHA	UTY
DUSP12	ZFY
DYNLRB1	
EIF1AX	
EIF1AY	
EIF2S3	
EIF3B	
EMSY_ENST00000533248	
EXOC5	
FAM120AOS	
FAM173B	
FAT3	
FBXO21	
FEZ2	
FGF12	
FMO5	
FNTA	
FOXJ3	
FUNDC1	
FXYD5	
GGPS1	
GLUD1	
GOLPH3	
GPATCH1	
HAUS6	
HIBADH	
HNRNPUL1	
HSF1	
IER3IP1	
IGFBPL1	
INSM1	
IP6K2	
ITGA10	

JAGN1
KDM5C
KDM5D
KDM6A
KDR
KIAA2026
LIFR
LPCAT1
LRP5
LRRC69
LRTOMT
LSM6
MAF1
MANBAL
MAPK9
MBIP
METTL1
MIEN1
MKRN2
MLLT11
MMS19
MRPL45
NAA15
NDUFA4
NDUFB9
NDUFS6
NECAB3
NINL
NRCAM
NSFL1C
NSMCE2
NUFIP1
NUP155
ODR4
OLIG3
OXSR1
P2RY2
PARP2
PDCD6IP
PDRG1
PEX11B
PLEKHF1
POU6F2
PPA1
PQLC2L
PRKAA1
PRKG2
PRPF3
PRUNE1_ENST00000368934
PSMA7
PSMD11
PSMD6
PUF60
PUM3
RAB6A
RANBP6
RBM10
RCL1
REEP1
RNF115
RNF123
RNF139
RPL14
RPL15
RPS4Y1
RPS6
SCRIB
SHARPIN
SHQ1
SLC12A9
SLC2A4RG
SLC45A4
SMAD4
SNAPC3
SNAPIN

SNF8
SPATA17
SRC
STK31
STXBP4
SUPV3L1
TALDO1
TAX1BP1
TCTN3
THOC2
THOC7
TIGD5
TMEM106B
TMEM45B
TNPO2
TOX4
TP53INP2
TRAPPC2
TRMT12
TSFM
TSKU
TXLNG
TXNL1
UBA2
UBA3
UGDH
UHRF2
USP4
USP9Y
UTY
VKORC1L1
VPS28
VPS52
VPS72
VWDE
WASHC5_ENST00000517845
WDR36
WDR70
WDR82
WDYHV1
YME1L1
ZFY
ZNF254
ZNF337
ZNF623
ZNF7
