# Supplementary material for: Multi-omics analysis identifies repurposing bortezomib in the treatment of kidney-, nervous system-, and hematological cancers
Source: Sci Rep. 2024 Aug 10;14:18576. doi: 10.1038/s41598-024-62339-x (PMC11316778; doi:10.1038/s41598-024-62339-x)
Supplement: Supplementary file 3 — Supplementary Table 2. [file 41598_2024_62339_MOESM3_ESM.pdf]

Supplementary Table 2: The 50 most mutated genes identified in insensitive cell line group and comparison across sensitivity groups

1= Mutated gene   X axis represent the 50 most mutated genes in insensitive cell lines (36 cell lines)
0= No mutated gene   Y axis represent all cell lines in Sanger COSMIC dataset (Complete mutation data)

Insensitive cell lines

Sample name	ANKRD36	ANKRD36C	CSMD1	CSMD3	DNAH11	DNAH17	DNAH6	DNAH8	DST	FSIP2	HERC2	HMCN1	HYDIN	LRP1B	LRP2	MGAM	MUC12	MUC16	MUC4	NBPF1	NEB	NRXN1	OBSCN	PCDH15	PCDHA1	PCDHA2	PCDHA3	PCDHA4	PCDHA5	PCDHA6	PCDHGA1	PCDHGA2	PCDHGA3	PDE4DIP	PKHD1	PKHD1L1	PLEKHB2	PTPRN2	RIMS2	RYR1	RYR2	RYR3	SPTA1	SYNE1	TP53	TRRAP	TSPEAR	TTN	USH2A	WDFY4
AsPC-1	1	1	1	0	0	0	0	1	0	0	0	1	1	1	1	0	1	0	1	1	1	1	0	0	1	1	1	1	1	1	0	0	0	0	0	0	0	0	1	1	1	1	0	0	1	0	0	1	1	1
BT-474	0	1	1	1	1	1	0	1	0	1	1	1	1	0	1	1	1	1	1	0	1	1	1	1	1	1	1	1	1	1	1	1	1	1	0	0	0	1	0	1	1	1	1	1	1	1	1	1	1	1
COLO-680N	1	1	0	1	1	0	1	1	1	0	1	1	1	1	0	1	1	1	1	1	1	1	1	0	0	0	0	0	0	0	1	1	1	1	0	1	1	1	0	0	0	1	0	1	1	1	0	1	1	1
COR-L88	1	1	1	1	1	1	1	1	0	0	0	1	1	1	1	0	1	0	1	0	1	0	1	1	0	0	0	0	0	0	0	0	0	1	1	0	1	1	1	0	1	1	1	1	1	1	0	1	1	1
DMS-79	1	1	1	1	0	1	1	1	1	1	0	1	1	1	1	1	1	1	0	1	0	1	1	0	0	0	0	0	0	0	0	0	0	1	1	0	1	1	1	1	1	1	1	1	0	0	0	1	1	0
EW-13	0	1	0	0	0	0	1	0	1	0	0	0	1	0	1	1	1	0	1	1	1	1	0	1	0	0	0	0	0	0	1	1	1	1	1	0	0	1	0	1	1	0	0	0	1	1	1	0	1	0
JHH-6	1	1	0	0	1	0	0	0	1	0	0	0	1	1	0	1	0	0	1	0	0	0	0	0	1	1	1	1	1	1	1	1	1	1	0	1	1	1	0	0	0	0	0	1	1	0	1	0	0	1
LU-165	0	1	0	1	1	1	1	1	1	1	1	1	1	0	1	0	0	1	1	1	1	0	1	0	1	1	1	1	1	1	0	0	0	1	0	1	1	0	0	0	1	1	1	1	1	1	1	1	1	0
LU-99A	1	1	1	1	0	0	1	0	0	1	1	1	1	1	0	0	0	1	1	1	1	0	1	0	1	1	1	1	1	1	1	1	1	1	1	0	1	0	1	0	1	0	0	1	0	1	1	1	1	1
NCI-H1435	0	1	1	1	1	1	1	1	1	1	0	1	1	1	1	1	1	1	1	0	1	1	1	1	1	1	1	1	1	1	1	1	1	0	1	1	1	1	1	1	1	1	1	1	1	0	1	1	1	1
NCI-H1563	1	1	0	1	1	1	1	0	0	1	1	0	1	1	1	1	1	1	1	0	1	0	1	1	1	1	1	1	1	1	1	1	0	1	1	1	1	0	1	1	1	1	1	1	0	1	1	1	1	1
NCI-H1651	1	1	1	1	0	1	1	1	1	0	1	1	1	0	1	1	0	1	1	1	1	1	1	1	1	1	1	1	1	1	1	1	1	1	0	1	1	1	1	1	1	1	1	1	1	0	1	1	1	0
NCI-H1770	1	1	1	1	1	0	1	1	1	1	1	1	1	1	1	1	1	1	1	1	1	1	1	1	1	1	1	1	1	1	0	0	0	1	1	1	1	1	1	1	1	1	1	1	1	0	0	1	1	1
NCI-H1793	1	1	1	1	0	1	1	0	0	1	1	1	1	1	1	1	1	1	0	0	1	1	1	1	1	1	1	1	1	1	1	1	1	1	1	1	0	1	1	1	1	1	1	1	1	1	0	1	1	1
NCI-H1838	0	1	0	1	0	0	0	1	1	1	1	1	1	1	1	1	1	1	0	1	1	1	1	1	1	1	1	1	1	1	0	0	0	1	1	1	0	1	1	0	1	1	1	1	1	0	1	1	1	1
NCI-H187	0	1	1	1	0	1	1	1	0	0	1	1	1	0	0	1	1	1	1	1	0	1	1	0	1	1	1	1	0	0	1	1	1	1	0	1	0	1	1	1	0	1	1	1	1	1	0	1	0	1
NCI-H1944	0	0	1	1	1	0	0	0	0	0	0	0	1	1	1	1	1	1	1	0	1	1	1	0	1	1	1	1	1	1	1	1	1	1	1	1	1	1	0	0	1	1	0	0	0	0	0	1	0	0
NCI-H2066	1	0	1	1	1	1	1	1	0	0	1	1	1	1	1	0	0	1	1	0	1	0	1	1	1	1	1	0	0	0	0	0	0	0	0	0	1	1	1	1	1	1	1	1	1	1	0	1	1	0
NCI-H211	1	1	1	0	1	1	1	1	1	1	1	1	1	1	1	1	1	1	1	1	1	0	1	1	1	1	1	1	1	1	1	1	1	1	1	0	1	0	1	1	1	1	0	1	1	1	1	1	1	0
NCI-H2342	0	1	1	1	1	1	1	1	1	1	1	1	1	1	1	1	1	1	1	0	1	1	1	1	1	1	1	1	1	1	1	1	1	1	1	1	1	1	1	1	1	1	1	1	1	1	1	1	1	1
NCI-H441	0	1	1	1	1	1	1	1	0	0	1	0	0	1	0	1	1	1	1	1	1	0	1	0	1	1	1	1	1	1	1	1	1	0	1	1	0	1	1	1	1	1	1	1	1	1	0	1	0	0
NCI-H727	1	0	1	1	1	1	1	0	0	1	1	1	1	1	1	1	1	1	1	1	0	1	1	1	1	1	1	1	1	1	1	1	1	0	0	0	1	1	1	1	1	1	1	0	1	1	1	1	0	1
NCI-H835	1	1	0	1	1	0	0	0	1	1	1	0	1	1	1	1	1	1	1	1	1	1	1	1	1	1	1	1	1	1	0	0	0	1	0	1	1	0	1	1	1	1	1	1	1	0	1	1	1	1
OCUM-1	1	1	0	0	1	0	0	1	1	1	0	1	1	1	0	0	1	1	1	1	1	1	0	1	1	1	0	0	0	0	0	0	0	0	0	0	1	1	0	1	1	1	0	1	1	1	1	1	0	1
PANC-03-27	0	0	1	0	1	1	1	1	1	0	0	1	0	0	0	1	1	0	1	1	0	0	1	1	0	0	0	0	0	0	0	0	0	1	1	1	1	0	0	1	1	0	1	0	1	0	1	0	0	0
RKN	1	1	1	0	1	0	0	0	1	1	1	0	1	1	0	0	1	1	1	1	0	1	0	1	1	1	1	1	1	1	0	0	0	1	0	1	0	0	0	1	0	1	0	0	1	1	1	1	0	1
SCH	1	0	1	0	1	1	1	1	0	0	1	1	0	0	0	0	0	1	0	0	1	0	1	1	0	0	0	0	0	0	1	1	1	1	1	0	1	0	1	1	0	1	1	1	1	0	1	1	1	1
SK-MEL-24	1	1	1	1	0	1	1	1	1	1	1	1	0	1	0	1	0	1	1	0	1	1	1	1	1	1	1	1	1	1	1	1	1	0	1	0	1	1	1	0	0	1	1	1	0	0	1	1	0	0
SK-MES-1	0	0	0	1	1	0	0	0	1	1	0	1	1	1	1	1	1	0	1	0	1	1	1	1	0	0	0	0	0	0	1	1	0	0	0	1	1	0	1	1	1	1	1	1	1	1	1	1	0	1
SKM-1	1	1	1	0	0	0	1	0	1	0	0	1	1	0	1	1	1	0	1	0	1	1	1	0	1	1	1	1	1	1	1	1	0	0	1	1	0	0	0	1	1	0	1	1	1	0	0	1	1	1
SN12C	1	0	1	1	1	0	1	0	0	1	1	1	1	1	1	0	1	1	0	1	1	1	1	1	0	0	0	0	0	0	1	1	1	1	0	1	1	1	0	1	1	1	1	1	1	1	1	1	1	0
Saos-2	1	1	1	1	1	1	0	0	1	1	0	0	1	0	0	1	1	1	1	1	0	0	0	1	1	1	1	1	1	1	0	0	0	1	1	1	0	0	0	1	0	0	1	0	0	0	1	1	1	0
TGBC24TKB	1	0	1	1	0	1	0	1	1	1	1	1	1	1	0	1	0	1	1	0	0	1	0	1	1	1	1	1	1	1	1	1	1	0	0	1	1	0	0	1	1	1	0	1	1	1	0	1	0	1
THP-1	1	1	1	0	0	1	0	1	1	1	1	0	1	1	0	1	1	0	1	1	0	0	0	0	0	0	0	0	0	0	1	1	1	0	1	1	0	1	0	0	0	1	1	1	1	0	1	1	1	0
VMRC-LCD	0	1	1	1	1	1	0	1	1	1	1	1	1	1	1	1	1	1	0	1	1	1	1	1	1	1	1	1	1	1	1	1	1	1	1	1	1	0	1	1	1	1	1	1	1	1	0	1	1	0
ZR-75-30	1	1	1	1	1	1	0	1	1	0	0	1	1	0	0	1	1	1	1	1	1	0	0	0	0	0	0	0	0	0	1	1	1	0	1	0	0	0	1	0	1	1	0	0	0	1	1	1	1	0

Sensitive cell lines

Sample name	ANKRD36	ANKRD36C	CSMD1	CSMD3	DNAH11	DNAH17	DNAH6	DNAH8	DST	FSIP2	HERC2	HMCN1	HYDIN	LRP1B	LRP2	MGAM	MUC12	MUC16	MUC4	NBPF1	NEB	NRXN1	OBSCN	PCDH15	PCDHA1	PCDHA2	PCDHA3	PCDHA4	PCDHA5	PCDHA6	PCDHGA1	PCDHGA2	PCDHGA3	PDE4DIP	PKHD1	PKHD1L1	PLEKHB2	PTPRN2	RIMS2	RYR1	RYR2	RYR3	SPTA1	SYNE1	TP53	TRRAP	TSPEAR	TTN	USH2A	WDFY4
A101D	1	0	0	1	0	1	1	1	0	0	0	0	1	1	0	0	0	1	0	1	0	0	1	0	0	0	0	0	0	0	0	0	0	0	0	1	1	0	1	0	1	1	0	0	0	0	1	1	0	0
A388	0	1	0	1	1	1	0	1	1	0	0	1	0	1	0	1	0	0	0	0	0	0	0	1	0	0	0	0	0	0	1	1	1	0	0	1	1	1	0	0	1	0	0	1	1	1	0	1	0	0
A704	1	1	1	1	0	0	1	1	1	0	1	0	1	1	1	1	0	0	1	0	1	0	0	0	0	0	0	0	0	0	1	1	1	1	1	0	1	0	0	1	1	0	1	0	1	0	0	1	0	0
AMO-1	1	0	1	1	1	1	0	0	1	1	0	1	1	1	1	1	1	1	1	1	0	1	0	0	1	1	1	1	1	1	1	1	1	1	1	1	1	1	0	1	1	1	0	1	0	0	1	1	0	0
BB30-HNC	0	0	0	0	0	0	0	0	0	0	1	0	0	0	0	0	0	0	1	0	0	0	0	0	1	1	1	1	1	1	1	1	1	0	0	0	0	0	0	0	0	0	0	0	1	0	0	0	0	1
BB65-RCC	0	0	0	0	0	0	0	0	1	0	0	0	0	0	0	0	0	0	1	0	0	0	1	0	0	0	0	0	0	0	0	0	0	0	0	1	0	0	0	0	0	0	0	0	1	1	0	1	0	0
BE-13	1	0	1	1	1	0	1	1	0	0	1	1	1	1	1	0	1	0	1	1	1	0	1	0	1	1	1	1	1	1	1	1	1	0	1	1	1	0	0	1	0	0	1	0	1	0	1	1	1	0
CMK	1	0	1	0	1	1	0	1	0	0	0	1	1	1	1	1	0	1	1	1	0	1	1	0	1	1	1	0	0	0	1	1	1	1	0	1	1	0	0	0	0	1	0	1	1	0	1	1	1	0
CP66-MEL	0	1	1	0	1	1	1	1	1	1	1	1	1	1	1	1	1	1	0	0	1	1	1	0	1	1	1	1	1	1	1	1	1	1	1	0	1	0	1	1	1	1	0	1	0	1	1	1	1	0
D-247MG	0	0	1	0	0	0	1	0	0	0	0	1	1	0	1	1	0	1	1	1	0	1	0	0	1	1	0	0	0	0	0	0	0	0	0	1	1	1	0	0	1	0	0	1	0	0	1	1	0	0
D-336MG	0	1	0	1	1	1	0	0	0	1	0	1	1	0	1	0	1	0	1	1	1	0	0	0	1	1	1	1	1	1	0	0	0	0	1	0	1	1	0	0	1	0	1	1	1	0	1	1	0	0
D-542MG	0	0	0	0	0	0	0	0	0	1	0	1	0	0	0	0	1	0	0	0	1	1	0	0	1	1	1	1	1	0	1	1	1	0	0	0	0	0	0	0	0	0	1	0	1	0	0	1	1	0
DB	0	1	1	1	1	0	0	0	1	1	0	1	1	0	0	1	0	1	1	1	1	0	1	1	1	1	1	1	1	1	1	1	1	0	0	1	1	0	0	0	0	1	0	1	1	0	1	1	1	1
DEL	1	1	1	0	1	0	0	1	1	0	0	0	0	1	1	1	0	0	1	1	0	0	1	0	1	1	1	1	1	1	0	0	0	0	0	1	0	0	0	0	1	0	0	1	1	0	1	1	0	0
ECC12	0	1	0	1	1	0	1	0	1	1	0	0	0	0	0	0	1	0	1	1	0	1	1	1	1	0	0	0	0	0	0	0	0	1	1	1	1	1	1	1	1	1	0	1	1	1	1	1	1	1
EHEB	1	0	1	0	1	0	0	1	0	0	0	0	1	0	1	1	1	1	1	1	0	0	0	0	0	0	0	0	0	0	1	1	1	0	0	1	1	0	0	0	1	1	0	0	0	0	1	1	0	0
EKVX	1	0	1	0	0	0	1	1	1	1	0	0	1	1	1	1	0	0	1	0	1	1	1	1	1	1	1	1	1	1	0	0	0	1	1	1	0	0	1	0	1	1	1	1	1	1	1	1	1	0
Farage	1	1	1	1	0	1	0	1	1	1	1	0	1	1	1	0	1	1	1	1	1	1	1	1	1	1	1	1	1	1	1	1	1	1	1	1	1	1	0	0	1	1	0	1	1	1	1	1	1	1
HA7-RCC	0	0	0	0	0	0	0	0	0	0	0	1	0	0	0	0	0	1	0	0	0	0	1	0	0	0	0	0	0	0	0	0	0	0	1	0	0	0	0	0	0	1	0	1	0	0	0	1	0	0
HEL	0	0	1	1	0	1	0	0	0	0	0	0	1	0	1	0	0	0	1	1	0	1	0	0	0	0	0	0	0	0	0	0	0	0	0	0	0	1	0	0	1	0	0	1	1	0	0	1	1	0
HT	0	1	1	1	1	0	1	1	1	1	1	1	1	1	1	1	1	1	1	1	1	1	1	1	1	1	1	1	1	1	1	1	1	0	1	1	1	1	1	1	1	1	0	1	1	1	1	1	1	1
HUTU-80	1	1	1	1	1	1	0	1	0	0	0	1	1	1	1	1	1	0	1	1	1	1	0	0	1	1	1	1	1	1	0	0	0	1	1	0	1	1	0	0	0	0	1	1	0	1	1	1	1	1
JM1	1	0	0	0	1	0	0	0	1	0	0	1	1	1	1	0	1	0	1	0	0	0	1	1	0	0	0	0	0	0	1	1	1	0	0	0	1	1	0	0	1	1	0	1	0	1	0	0	1	0
KNS-42	0	0	0	0	0	0	0	1	1	0	1	0	0	1	0	1	0	1	1	1	1	0	1	0	0	0	0	0	0	0	0	0	0	1	1	0	0	0	0	0	0	1	0	1	1	1	0	1	1	1
KNS-81-FD	1	1	0	0	1	0	0	0	0	1	0	1	1	0	1	0	1	0	1	0	1	1	0	0	1	1	1	1	1	0	1	1	1	1	1	1	0	1	0	0	1	1	1	1	0	0	0	1	1	0
LAN-6	1	1	0	0	1	1	1	0	0	0	1	1	1	0	0	1	0	1	0	0	1	0	0	0	0	0	0	0	0	0	0	0	0	0	0	1	1	1	0	1	0	1	0	0	1	0	1	1	0	1
LB1047-RCC	1	0	1	1	0	0	1	0	1	0	0	0	0	0	0	0	0	0	0	0	0	0	0	0	0	0	0	0	0	0	0	0	0	0	0	0	0	0	0	0	0	0	0	0	0	0	0	0	0	0
LB2241-RCC	0	0	0	0	0	0	0	0	0	1	0	0	0	0	0	0	1	1	0	0	0	0	0	0	0	0	0	0	0	0	1	1	1	0	0	0	0	0	0	0	0	0	0	0	0	0	0	1	0	0
LB2518-MEL	0	0	1	0	1	1	0	1	1	1	0	0	1	1	0	1	0	1	1	0	0	1	1	0	0	0	0	0	0	0	1	1	0	0	1	1	1	0	0	1	0	0	1	1	0	0	1	1	1	1
LB373-MEL-D	1	0	0	0	0	0	0	0	0	0	0	1	1	0	1	0	0	0	0	0	0	0	1	0	1	1	1	1	1	0	0	0	0	0	0	0	1	0	0	0	0	1	0	0	0	0	0	1	0	0
LC-2-ad	0	0	1	1	1	1	0	1	0	0	0	1	1	0	0	1	0	1	1	1	1	0	1	1	0	0	0	0	0	0	1	1	1	1	0	1	1	0	1	0	1	1	1	1	1	1	1	1	1	1
MMAC-SF	1	1	1	1	1	1	1	0	0	0	1	0	1	0	0	0	0	1	0	0	0	1	1	0	0	0	0	0	0	0	1	1	0	1	0	0	1	0	0	1	0	1	1	1	0	1	1	1	1	0
MY-M12	1	0	1	1	1	1	0	0	1	0	1	0	1	0	0	0	0	1	1	1	1	0	0	0	1	1	1	1	1	1	1	1	1	0	0	1	0	0	0	1	0	1	0	1	0	1	1	1	0	1
MZ2-MEL	0	1	0	1	0	1	1	0	0	1	1	0	1	1	1	1	1	1	1	1	1	1	1	1	1	1	1	1	1	1	1	1	1	0	0	1	1	1	0	0	0	1	0	0	0	1	1	1	0	0
MZ7-mel	1	1	1	1	1	1	1	1	1	1	1	1	1	1	1	0	1	1	1	0	1	1	1	1	1	1	1	1	1	1	1	1	1	1	1	1	1	1	1	1	1	1	1	1	0	1	1	1	1	1
NK-92MI	1	1	0	1	0	1	1	1	1	0	1	0	0	1	1	1	0	1	1	1	1	1	1	1	1	1	1	1	1	1	1	1	1	0	1	1	1	1	0	1	0	0	0	1	1	0	0	1	1	0
NU-DUL-1	0	0	1	1	1	1	0	0	1	1	1	0	0	1	0	1	1	0	1	1	1	1	1	1	1	1	1	1	1	1	1	1	1	1	1	1	0	0	1	1	1	0	0	1	1	0	0	1	1	1
OCI-AML5	0	1	1	1	0	0	1	0	1	0	0	0	0	1	0	0	0	0	1	1	1	1	0	1	1	1	1	1	1	1	1	1	1	0	1	1	1	0	1	1	1	1	0	0	0	0	0	1	0	0
OCI-LY-19	1	1	0	1	1	0	0	0	0	0	0	0	1	0	1	0	1	0	1	0	1	0	0	0	0	0	0	0	0	0	1	1	1	0	0	0	0	0	0	0	0	0	0	0	0	0	0	1	0	1
OCI-M1	0	1	1	1	0	1	0	0	1	0	0	1	1	1	1	0	0	0	1	0	0	1	1	0	1	1	1	1	0	0	0	0	0	0	1	0	1	0	0	0	0	1	0	1	1	0	0	1	0	1
QIMR-WIL	0	0	0	0	1	1	0	1	1	0	0	1	1	1	0	1	0	0	1	0	0	0	0	0	1	1	1	1	1	1	1	1	1	1	1	0	0	0	0	0	1	1	0	1	0	0	1	0	0	0
RXF393	1	1	1	1	1	0	1	0	0	0	0	0	1	0	0	0	0	1	0	1	1	0	1	0	0	0	0	0	0	0	1	1	1	0	1	0	1	1	0	0	1	0	0	0	1	0	1	1	0	0
SF126	0	1	1	0	0	0	0	0	0	0	0	0	1	1	0	1	0	0	1	1	1	1	0	0	1	1	1	1	1	1	0	0	0	0	0	0	1	1	1	1	0	1	0	0	1	0	1	1	0	0
SNB75	1	1	1	1	1	0	0	0	0	1	0	0	1	0	1	1	1	1	1	1	1	0	1	0	0	0	0	0	0	0	1	1	1	1	1	0	0	0	0	0	0	0	0	0	1	1	0	1	0	0
SU-DHL-16	0	0	1	1	1	0	1	0	1	0	0	1	0	0	0	0	1	0	1	0	1	0	0	1	1	1	0	0	0	0	0	0	0	0	1	0	1	0	0	0	0	1	0	1	1	0	1	0	0	0
SU-DHL-4	1	1	1	1	1	0	1	1	0	0	1	1	1	1	1	0	1	1	1	0	1	0	1	1	1	1	1	1	1	1	1	1	1	1	1	0	1	1	1	0	0	0	0	0	1	1	0	1	0	0
TE-10	1	0	0	0	0	1	1	1	0	1	1	1	1	1	1	0	0	0	1	1	0	0	1	0	0	0	0	0	0	0	0	0	0	1	0	1	0	0	1	0	0	1	0	1	1	1	1	1	0	1
no-10	0	1	0	1	0	0	0	1	1	0	1	1	1	1	0	1	0	0	1	0	1	0	0	0	1	1	1	1	1	1	1	1	1	0	1	1	0	0	0	0	0	0	1	1	1	0	1	0	1	0
no-11	1	1	0	0	1	0	1	0	1	1	0	1	1	1	0	0	0	1	1	0	1	0	0	0	1	1	1	0	0	0	0	0	0	1	1	0	1	0	1	1	0	0	0	0	1	0	1	1	1	1

Average mutated genes per cell line in each cell line group

	ANKRD36	ANKRD36C	CSMD1	CSMD3	DNAH11	DNAH17	DNAH6	DNAH8	DST	FSIP2	HERC2	HMCN1	HYDIN	LRP1B	LRP2	MGAM	MUC12	MUC16	MUC4	NBPF1	NEB	NRXN1	OBSCN	PCDH15	PCDHA1	PCDHA2	PCDHA3	PCDHA4	PCDHA5	PCDHA6	PCDHGA1	PCDHGA2	PCDHGA3	PDE4DIP	PKHD1	PKHD1L1	PLEKHB2	PTPRN2	RIMS2	RYR1	RYR2	RYR3	SPTA1	SYNE1	TP53	TRRAP	TSPEAR	TTN	USH2A	WDFY4
Average insensitive	0,66667	0,7777778	0,75	0,72222	0,66667	0,61111	0,61111	0,63889	0,63889	0,61111	0,63889	0,75	0,88889	0,72222	0,61111	0,75	0,77778	0,77778	0,83333	0,61111	0,75	0,61111	0,75	0,66667	0,72222	0,72222	0,69444	0,66667	0,63889	0,63889	0,66667	0,66667	0,58333	0,66667	0,58333	0,63889	0,66667	0,58333	0,61111	0,72222	0,77778	0,83333	0,69444	0,77778	0,80556	0,58333	0,63889	0,91667	0,69444	0,58333
Average sensitive	0,5102	0,5102041	0,57143	0,57143	0,57143	0,42857	0,40816	0,42857	0,5102	0,36735	0,34694	0,5102	0,71429	0,55102	0,5102	0,46939	0,40816	0,4898	0,77551	0,5102	0,57143	0,42857	0,55102	0,30612	0,61224	0,59184	0,55102	0,5102	0,4898	0,42857	0,61224	0,61224	0,57143	0,38776	0,53061	0,53061	0,63265	0,36735	0,26531	0,32653	0,44898	0,55102	0,2449	0,61224	0,59184	0,36735	0,59184	0,87755	0,44898	0,34694

Mutation frequence between insensitive (36) and sensitive (49) cell lines. The intermediate was excluded in this analysis (922)

p-value	0,15272	0,0115114	0,09071	0,1572	0,37933	0,09849	0,06568	0,05614	0,24211	0,02609	0,0073	0,02496	0,05238	0,11	0,36115	0,009	0,00052	0,0067	0,51594	0,36115	0,09071	0,09849	0,06078	0,00078	0,29623	0,21864	0,18421	0,15272	0,17595	0,05614	0,61158	0,61158	0,91389	0,01067	0,63391	0,32394	0,74932	0,04904	0,00113	0,00022	0,00203	0,00581	1,7E-05	0,10777	0,03684	0,04904	0,66461	0,56791	0,02446	0,03044
Genes	ANKRD36	ANKRD36C	CSMD1	CSMD3	DNAH11	DNAH17	DNAH6	DNAH8	DST	FSIP2	HERC2	HMCN1	HYDIN	LRP1B	LRP2	MGAM	MUC12	MUC16	MUC4	NBPF1	NEB	NRXN1	OBSCN	PCDH15	PCDHA1	PCDHA2	PCDHA3	PCDHA4	PCDHA5	PCDHA6	PCDHGA1	PCDHGA2	PCDHGA3	PDE4DIP	PKHD1	PKHD1L1	PLEKHB2	PTPRN2	RIMS2	RYR1	RYR2	RYR3	SPTA1	SYNE1	TP53	TRRAP	TSPEAR	TTN	USH2A	WDFY4
